# Revealing of Intracellular Antioxidants in *Dendrobium nobile* by High Performance Liquid Chromatography-Tandem Mass Spectrometry

**DOI:** 10.3390/metabo13060702

**Published:** 2023-05-28

**Authors:** Dan Rao, Ruoxi Zhao, Yadong Hu, Hongjie Li, Ze Chun, Shigang Zheng

**Affiliations:** 1 CAS Key Laboratory of Mountain Ecological Restoration and Bioresource Utilization & Ecological Restoration, Biodiversity Conservation Key Laboratory of Sichuan Province, Chengdu Institute of Biology, Chinese Academy of Sciences, Chengdu 610041, China; 2 College of Life Sciences, University of Chinese Academy of Sciences, Beijing 100041, China; 3 Innovation Academy for Seed Design, Chinese Academy of Sciences, Beijing 100101, China; 4 Xiong’an Institute of Innovation, Chinese Academy of Sciences, Baoding 071000, China

**Keywords:** medicinal plant, oxidative damage, H293T, ROS, CAT, SOD, saccharides, phenols, molecular weight, polarity

## Abstract

The medicinal plant *Dendrobium nobile* is an important natural antioxidant resource. To reveal the antioxidants of *D. nobile*, high performance liquid chromatography-tandem mass spectrometry (HPLC-MS/MS) was employed for metabolic analysis. The H_2_O_2_-induced oxidative damage was used in human embryonic kidney 293T (H293T) cells to assess intracellular antioxidant activities. Cells incubated with flower and fruit extracts showed better cell survival, lower levels of reactive oxygen species (ROS), and higher catalase and superoxide dismutase activities than those incubated with root, stem, and leaf extracts (*p* < 0.01). A total of 13 compounds were newly identified as intracellular antioxidants by association analysis, including coniferin, galactinol, trehalose, beta-D-lactose, trigonelline, nicotinamide-N-oxide, shikimic acid, 5′-deoxy-5′-(methylthio)adenosine, salicylic acid, isorhamnetin-3-O-neohespeidoside, methylhesperidin, 4-hydroxybenzoic acid, and cis-aconitic acid (R^2^ > 0.8, Log_2_FC > 1, distribution > 0.1%, and *p* < 0.01). They showed lower molecular weight and higher polarity, compared to previously identified in vitro antioxidants in *D. nobile* (*p* < 0.01). The credibility of HPLC-MS/MS relative quantification was verified by common methods. In conclusion, some saccharides and phenols with low molecular weight and high polarity helped protect H293T cells from oxidative damage by increasing the activities of intracellular antioxidant enzymes and reducing intracellular ROS levels. The results enriched the database of safe and effective intracellular antioxidants in medicinal plants.

## 1. Introduction

Oxidative stress is closely related to human health and many types of disease, such as ageing, cardiovascular disease, chronic obstructive pulmonary disease, Alzheimer’s disease, and cancer [1]. Therefore, safe and effective antioxidants are abundantly needed for health care products and pharmaceuticals [2,3].

Medicinal plants, as important natural antioxidant resources, have attracted more and more attention in recent years [3,4]. The antioxidant capacity was reported in extracts from many medicinal plants around the world, such as *Crataegus oxyacantha*, *Hamamelis virginiana*, *Hydrastis canadensis*, *Salvia nubicola*, *Acer oblongifolium*, *Hedera nepalensis*, *Curcuma longa*, *Zingiber officinale*, *Piper nigrum*, and *Piper longum* [5,6,7]. Some species of *Dendrobium*, a widely used medicinal plant in Southeast Asia, also showed antioxidant effects in vitro and in vivo. Extracts from *D. Officinale*, *D. catenatum*, *D. huoshanense*, *D. candidum*, *D. crepidatum*, *D. moniliforme*, *D. chrysotoxum*, and *D. tosaense* showed protective effects on oxidative damages in PC12 cells, U251 cells, HFF-1 cells, Jurkat cells, H9c2 cells, and B16/F10 cells [8,9,10,11,12]. *D. nobile* extracts also showed beneficial effects in protecting cells from oxidative damage [13,14,15,16].

For better application in the health products industry and pharmaceutical industry, the core chemical compounds contributing to antioxidant activities need to be identified in *Dendrobium* [3,17]. To date, only some of the polysaccharides, flavonoids, and bibenzyls have been reported to be related to antioxidant activities in *D. nobile*, *D. officinale*, *D. huoshanense*, *D. candidum*, *D. loddigesii*, and *D. pachyglossum* [8,11,12,15,18]. Comprehensive and systematic analyses are still required for the antioxidant basis of *Dendrobium*. Along with the development of high performance liquid chromatography-mass spectrometry (HPLC-MS) technology, it has been used for metabolic analysis, chemical differentiation, quality control, and pharmaceutical identification in medicinal plants [4,14,19]. The recently established quasi-targeted metabolomics based on multiple reaction monitoring (MRM) enables HPLC-MS/MS to quickly, extensively, and accurately identify metabolites [14,20,21]. It will be helpful for functional-metabolic co-analysis in *Dendrobium* species [14,22].

HPLC-MS/MS is used in this article for quantitative analysis of secondary metabolites among different tissues of *D. nobile*. Intracellular antioxidant activities are evaluated by H_2_O_2_-induced oxidative damage in human embryonic kidney 293T cells (H293T). The key chemical basis is then revealed by a co-analysis of antioxidant activities and secondary metabolites.

## 2. Materials and Methods

### 2.1. Plant Materials

Fresh roots, stems, leaves, flowers, and fruits of *D. nobile* were obtained from Hejiang, Sichuan Province (28°49′ N, 105°50′ E). Roots, stems, leaves, and flowers were collected in May 2019 and May 2020, and fruits were collected in November 2019 and November 2020. The tissue samples were obtained from more than 30 individual plants for each collection. The tissue samples were washed with pure water, dried at 40 °C for a week, ground in powder, and screened using a 50 mesh sieve for the extraction of metabolites.

### 2.2. Cell Lines and Chemical Reagents

H293T cell lines were purchased from the Shanghai Institute of Cell Biology (Shanghai, China). Dulbecco’s modified eagle’s medium-high glucose (DMEM), penicillin/streptomycin, trypsin, and phosphate buffered saline (PBS) were purchased from Wisent Biotechnology Co., Ltd. (Nanjing, China). Fetal bovine serum (FBS) was purchased from Gibco Inc. (Canyon, OR, USA). The bovine serum albumin (BSA) and bicinchoninic acid (BCA) protein assay kit were purchased from Solarbio Biotechnology Co., Ltd. (Beijing, China). Dimethyl sulfoxide (DMSO), radio immuno-precipitation assay (RIPA) buffer, phenylmethylsulfonyl fluoride (PMSF), total superoxide dismutase (SOD) assay kit with WST-8, catalase (CAT) assay kit, and reactive oxygen species (ROS) assay kit were purchased from Beyotime Biotechnology Co., Ltd. (Shanghai, China). Cell counting kit-8 (CCK-8) was purchased from Tongren Chemical Research Institute (Kumamoto, Japan).

### 2.3. Metabolites Extraction for HPLC-MS/MS

Each 100 mg fine powdered sample was suspended with a 500 μL prechilled solution (80% methanol contained 0.1% formic acid) by well vortexing. The sample was incubated for 5 min and then centrifuged at 15,000 rpm for 10 min. The supernatant was diluted to a final concentration of 53% methanol by pure water. The sample was then transferred to a new tube and then centrifuged at 15,000 rpm for 20 min. The supernatant was used for chromatography [14].

### 2.4. Metabolites Extraction for Bioactivity Analysis

Each 5 g fine powdered sample was immersed in a 200 mL solution (80% methanol contained 0.1% formic acid) at room temperature for 24 h and then filtered to remove the residues. The filtrates were subsequently condensed in a rotary evaporator at 40 °C for 2 h and then were evaporated under vacuum for final drying. The dry extracts were dissolved with DMSO at a concentration of 100 mg/mL and diluted with DMEM medium to a final concentration of 50 μg/mL and 100 μg/mL for intracellular analysis [19].

### 2.5. HPLC-MS/MS Analysis

HPLC-MS/MS analyses were performed using an ExionLC™ AD system coupled with a QTRAP^®^ 6500+ mass spectrometer (AB Sciex Pte. Ltd., Framingham, MA, USA) in Novogene Co., Ltd. (Beijing, China). Positive ion mode: Sample was injected onto a BEH C8 column (100 × 2.1 mm, 1.9 μm) using a 30 min linear gradient at a flow rate of 0.35 mL/min in the positive polarity mode. The eluents were eluent A (0.1% formic acid-water) and eluent B (0.1% formic acid-acetonitrile). The solvent gradient was established as follows: 5% B, 1 min; 5–100% B, 24.0 min; 100% B, 28.0 min; 100–5% B, 28.1 min; 5% B, 30 min. QTRAP^®^ 6500+ mass spectrometer was operated in positive polarity mode with curtain gas of 35 psi, collision gas of medium, ionspray voltage of 5500 V, temperature of 500 °C, ion source gas of 1:55, and ion source gas of 2:55. Negative ion mode: Sample was injected onto a HSS T3 column (100 mm × 2.1 mm) using a 25 min linear gradient at a flow rate of 0.35 mL/min in the negative polarity mode. The eluents were eluent A (0.1% formic acid-water) and eluent B (0.1% formic acid-acetonitrile). The solvent gradient was set as follows: 2% B, 1 min; 2–100% B, 18.0 min; 100% B, 22.0 min; 100–5% B, 22.1 min; 5% B, 25 min. QTRAP^®^ 6500+ mass spectrometer was operated in positive polarity mode with curtain gas of 35 psi, collision gas of medium, ionspray voltage of −4500 V, temperature of 500 °C, ion source gas of 1:55, and ion source gas of 2:55 [14].

### 2.6. Standards Database of novoDB

Chemical standards were used for key parameter collection under the chromatographic and mass spectrometry conditions above. Finally, a total of six parameters, including parent ion (Q1), daughter ion (Q3), declustering potential (DP), collision energy (CE), molecular weight (MW), and retention time (RT), were stored for each specific compound in novoDB database. Then, it was used for quasi-targeted metabolic analysis under a certain LC-MS/MS method [20,22]. Currently, more than 3250 plant compounds can be employed in the novoDB database (https://cn.novogene.com/, accessed on 25 May 2023).

### 2.7. Metabolites Identification by Multiple Reaction Monitoring

To quickly, accurately, and extensively identify metabolites in the extracts of *D. nobile*, MRM was used for scanning mainly based on the above six key parameters [21]. For the Q1/Q3 scan, ±0.7 was set, and 0–300 was set for the DP scan; ±150 was set for the CE scan, and ±0.01 was set for the RT scan. If a compound matches a standard within the set scanning channel, this compound is detected as the standard. The MS parameters and chromatographic signals of all matched compounds were exported as a raw data file for further analysis.

### 2.8. Metabolites Quantification

The data files generated by HPLC-MS/MS were processed using SCIEX OS Version 1.4 (AB Sciex Pte. Ltd., Framingham, MA, USA) to integrate and correct the peak. The main parameters were established as a minimum peak height of 500, a signal/noise ratio of 5, and a Gaussian smooth width of 1. The screened signal peaks were used for peak area integration. The peak area of Q3 was used for relative quantification of the corresponding metabolite [22].

### 2.9. Metabolites Annotation

These metabolites were further annotated using the KEGG database (http://www.genome.jp/kegg/, accessed on 25 May 2023), the HMDB database (http://www.hmdb.ca/, accessed on 25 May 2023), and the Lipidmaps database (http://www.lipidmaps.org/, accessed on 25 May 2023) [14]. These annotations were used for a final classification of each compound.

### 2.10. Cell Survival Assay under H_2_O_2_

H293T cells were cultured in DMEM with 10% fetal bovine serum and 1% penicillin/streptomycin in a humidified atmosphere with 5% CO_2_ at 37 °C. Each 100 μL of cell culture included 4 × 10^4^ cells and was transferred to a new 96-well plate. The cells were then incubated with extracts (50 µg/mL, 100 µg/mL) or the same amount of DMSO for 24 h and then exposed to H_2_O_2_ for 4 h. A half-maximum inhibitory concentration (IC50) of 200 µM was used for a further H_2_O_2_-inducing assay (Figure S1). Then, the CCK-8 reagent was added to each well and incubated for 3 h at 37 °C. The absorbance was measured at 450 nm with a microplate reader. Cell survival rate = (As − Am)/(Ac − Am) × 100% (As: absorbance of sample; Am: absorbance of medium; Ac: absorbance of control) [13,23].

### 2.11. Detection of ROS Levels

After induction by H_2_O_2_, H293T cells were collected for detection using a reactive oxygen species assay kit. The fluorescence probe was diluted to 10 μM by DMEM without fetal bovine serum. Then, it was added to cover the collected cells without culture medium. After co-culturing at 37 °C for 20 min, the cells were washed with DMEM without fetal bovine serum three times. Finally, cells were detected by the Thermo scientific Varioskan Flash (Thermo Fisher, Waltham, Massachusetts, USA) at an excitation wavelength of 488 nm and an emission wavelength of 525 nm. Rosup was used as a positive control [13].

### 2.12. Cell Lysis

After induction by H_2_O_2_, H293T cells were collected and washed with 500 µL of 1× PBS (pH 7.4, without calcium and magnesium) three times. The cells were then resuspended by 1 mL of 1× PBS and transferred to a 1.5 mL centrifuge tube for centrifugation at 1500 g for 5 min under 4 °C. After removing the supernatant, 200 µL of RIPA buffer (pH 7.4, 50 mM Tris, 150 mM NaCl, 1% Triton X-100, 1% sodium deoxycholate, 0.1% SDS) containing 1 mM PMSF was added to the lysis cells for 20 min at 4 °C. The lysate was centrifuged at 12,000× *g* for 10 min under 4 °C. Each 20 µL of the supernatant was used for detection of total protein, SOD activity, and CAT activity.

### 2.13. Detection of Total Proteins

Each 20 μL of cell lysis solution was added with 200 μL of BCA working solution (50:1 of bicinchoninic acid and Cu reagent). After mixing well, they were placed at 37 °C for 30 min. The absorbance at 562 nm was used for calculation of total proteins with BSA standard [14,19]. The standard curve of BSA is shown in Figure S2A.

### 2.14. Detection of SOD Enzyme Activities

Each 20 μL of cell lysis solution was added with 151 μL SOD detection solution, 8 μL WST-8, 1 μL enzyme buffer, and 20 μL reaction start solution. After mixing well, they were placed at 37 °C for 30 min. Then, it was detected at an absorbance of A450 by the Thermo scientific Varioskan Flash (Thermo Fisher, Waltham, MA, USA). When the inhibition rate of the xanthine oxidase coupling reaction system is 50%, the SOD activity in the reaction system is defined as one unit of enzyme activity (1 unit). SOD activity = ((A_450 control_ − A_450sample_)/A_450 control_ × 100%/(1 − ((A_450 control_ − A_450sample_)/A_450 control_ × 100%))/(20 × protein concentration) [13].

### 2.15. Detection of CAT Enzyme Activities

Each 20 μL of cell lysis solution was added with 20 μL CAT buffer and 10 μL hydrogen peroxide solution (250 mM). After mixing well, they were placed at 25 °C for 5 min. Then, a 450 μL stopping solution was added to stop the reaction. Each 10 μL of the reaction solution was added with 40 μL CAT buffer. Each 10 μL of the mixed solution was added with a 200 μL chromogenic solution. After placing at 25 °C for 20 min, it was detected at an absorbance of A_520_ by the Thermo scientific Varioskan Flash (Thermo Fisher, Waltham, MA, USA). One unit of enzyme activity (1 unit) of CAT means that it can catalyze the decomposition of 1 micromole hydrogen peroxide within 1 min at 25 °C and pH 7.0 [13]. CAT activity = ((A_520 control_ − b)/k − (A_520 sample_ − b)/k) × 250/(20 × 20 × protein concentration). The b and k are the constant of the standard curve of hydrogen peroxide concentration (Figure S2D).

### 2.16. Detection of Total Soluble Saccharides

Each 0.25 g fine powdered sample was used for extraction with 100 mL of 80% ethanol solution. The filtered residue was used for extraction with 100 mL of pure water once more. Reflux extraction was performed at 40 °C for 1 h. All filtrates were collected and added to a final volume of 500 mL. Then, 2 mL of extracts, 1 mL of 5% phenol solution, and 5 mL of H_2_SO_4_ were mixed and kept in a boiling water bath for 20 min. After cooling, it was detected at an absorbance of A_490_ by Thermo scientific Varioskan Flash (Thermo Fisher, Waltham, MA, USA). Glucose was used for the calculation of the saccharide content [19]. The standard curve of glucose is shown in Figure S2B.

### 2.17. Detection of Total Phenols

Each 0.2 g fine powdered sample was used for extraction with 50 mL of pure water at 100 °C for 30 min. Each 2.5 mL filtrate of the extracts was added to 30 mL of 60% ethanol. Ultrasound extraction was performed at room temperature for 10 min. After filtration, the final volume was constant to 40 mL by 60% ethanol. Each 1 mL final extraction was added with 2.5 mL Folin–Ciocalteu reagent and 2.5 mL 15% sodium carbonate solution. The final volume was constant to 10 mL by pure water. After well mixing, they were placed at 40 °C for 1 h and room temperature for 20 min. The supernatant was used for detection at an absorbance of A_778_. Gallic acid monohydrate was used for the calculation of the content of total phenols [18,24]. The standard curve of gallic acid is shown in Figure S2C.

### 2.18. Statistics Analysis

The entire experimental procedure is shown in Figure S3. All measurements and experiments were repeated three times, and the data were expressed as the mean ± standard deviation (SD). Log_2_(fold change) (Log_2_(FC)) was used for the comparison of metabolic data. Correlation analysis was performed using PASW statistics 18.0 (International Business Machines Corporation, New York, USA). Pearson’s correlation coefficients and *p*-value were used to evaluate the correlations. Student’s *t*-test was used for comparison between two groups. One-way analysis of variance was used for comparison among three and more groups.

## 3. Results

### 3.1. Fine Capacity to H_2_O_2_ Induction in Flower and Fruit Extracts of D. nobile

**Figure 1:**
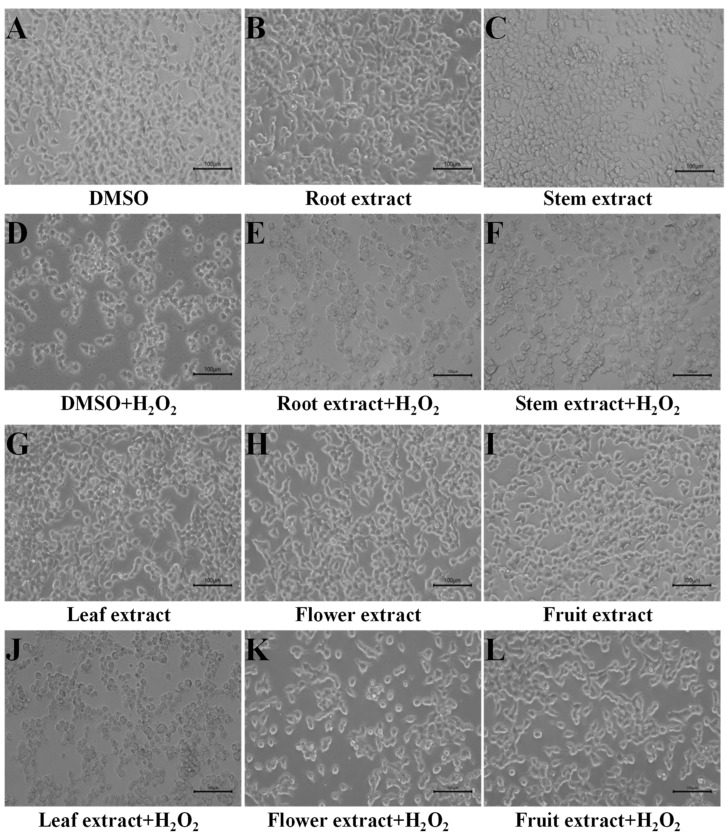
Response of H293T cells to H_2_O_2_-induced damage supplied with extracts from different parts of *D. nobile*. (**A**–**C**,**G**–**I**) H293T cells under DMSO or 50 µg/mL of *D. nobile* extracts without H_2_O_2_. (**D**–**F**,**J**–**L**) H293T cells under DMSO or 50 µg/mL of *D. nobile* extracts with 200 µM H_2_O_2_.

**Figure 2:**
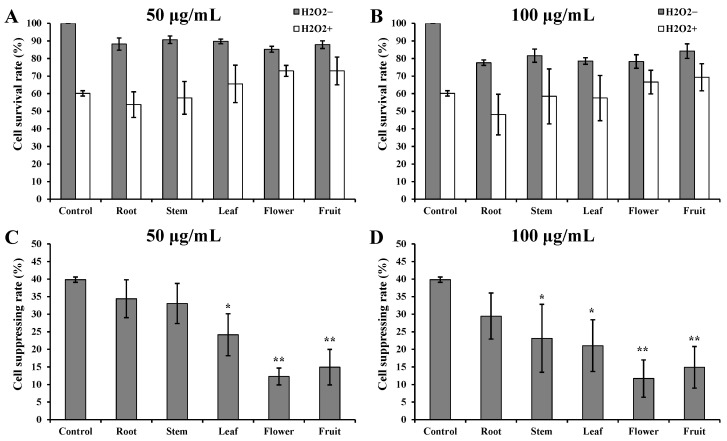
Comparison of survival rates and suppressing rates in H293T cells with H_2_O_2_-induced damage. (**A**) Survival rates under 50 µg/mL of extracts from different parts of *D. nobile*. (**B**) Survival rates under 100 µg/mL of extracts from different parts of *D. nobile*. (**C**) Suppressing rates under 50 µg/mL of extracts from different parts of *D. nobile*. (**D**) Suppressing rates under 100 µg/mL of extracts from different parts of *D. nobile*. * *p* < 0.05, ** *p* < 0.01 compared to control group.

H_2_O_2_ induction caused severe damage to cell morphology and cell viability (Figure 1). However, H293T cells incubated with *D. nobile* extracts showed better cell states than those incubated with DMSO only. The cells incubated with flower or fruit extracts showed an almost normal cell state to the cells without H_2_O_2_ induction. At a concentration of 50 µg/mL, the cell survival rates of root, stem, and leaf groups were approximately 60%. The cell survival rates of flower and fruit groups were more than 70% (Figure 2A). At a concentration of 100 µg/mL, the cell survival rates of root, stem, and leaf groups were less than 60%. The cell survival rates of flower and fruit groups were more than 65% (Figure 2B). Under both concentrations, the relative cell suppressing rates in root and stem groups showed no significant variances to the control group (Figure 2C,D). The relative cell suppressing rates in the leaf group were significantly lower than those in control group (*p* < 0.05). The relative cell suppressing rates in the flower and fruit groups were extremely significantly lower than those in control group (*p* < 0.01). Better cell states, higher survival rates, and lower suppression rates indicate that flower and fruit extracts of *D. nobile* possess a fine capacity in response to induction of H_2_O_2_.

### 3.2. Good Intracellular ROS Scavenging Effects of Flower and Fruit Extracts

**Figure 3:**
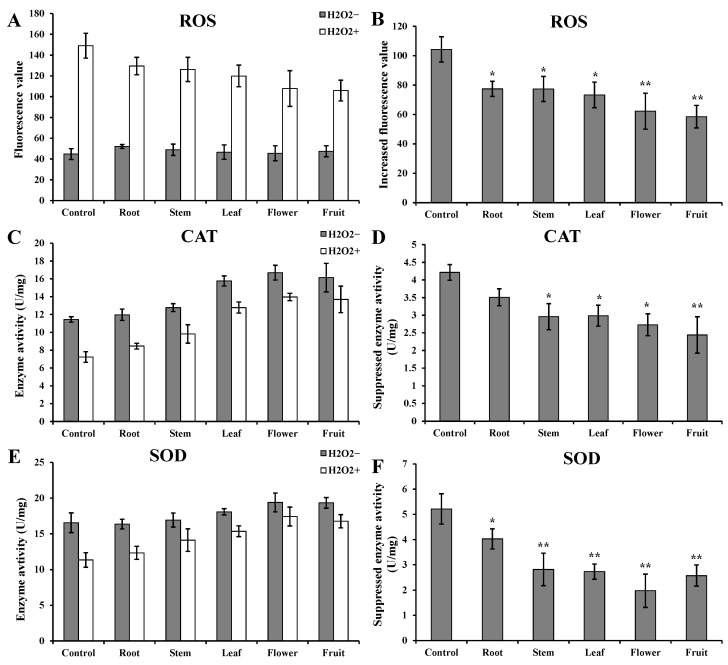
Comparison of ROS levels, CAT activities, and SOD activities in response to H_2_O_2_ induction. (**A**) ROS levels detected in H293T cells. (**B**) Comparison of increased ROS levels. (**C**) CAT activities detected in H293T cells. (**D**) Comparison of suppressed CAT activities. (**E**) SOD activities detected in H293T cells. (**F**) Comparison of suppressed SOD activities. Cells were incubated with 50 µg/mL of *D. nobile* extracts and induced by 200 µM H_2_O_2_. * *p* < 0.05, ** *p* < 0.01 compared to control group.

ROS levels in H293T cells increased substantially after H_2_O_2_ induction (Figure 3A). ROS levels showed no significant increase after incubating with *D. nobile* extracts without H_2_O_2_ induction. The increased ROS levels in the root, stem, and leaf groups were significantly lower than those in the control group (Figure 3B, *p* < 0.05). The increased ROS levels in flower and fruit groups were extremely significantly lower than those in the control group (*p* < 0.01). These results clearly show the good intracellular ROS scavenging effects of *D. nobile* extracts, especially from flowers and fruits.

### 3.3. Improved CAT and SOD Activities by Flower and Fruit Extracts

CAT and SOD activities were substantially suppressed by H_2_O_2_ induction (Figure 3C,E). However, CAT and SOD activities were improved by incubating with *D. nobile* extracts in all groups with or without H_2_O_2_ induction. The relatively suppressed CAT activity in root group showed no significant variance to that in control group (Figure 3D). The relatively suppressed CAT activities in the stem, leaf, and flower groups were significantly lower than those in the control group (*p* < 0.05). The relatively suppressed CAT activities in the fruit group were extremely significantly lower than those in the control group (*p* < 0.01). The relatively suppressed SOD activities in the root group were significantly lower than those in the control group (Figure 3F, *p* < 0.05). The relatively suppressed SOD activities in the stem, leaf, flower, and fruit group were extremely significantly lower than those in the control group (*p* < 0.01). These results suggest that pretreatment with flower and fruit extracts can significantly help to improve CAT and SOD enzyme activities in H293T cells, which is beneficial for reducing oxidative damage.

### 3.4. Evaluation of the Stability and Reliability in HPLC-MS/MS

**Figure 4:**
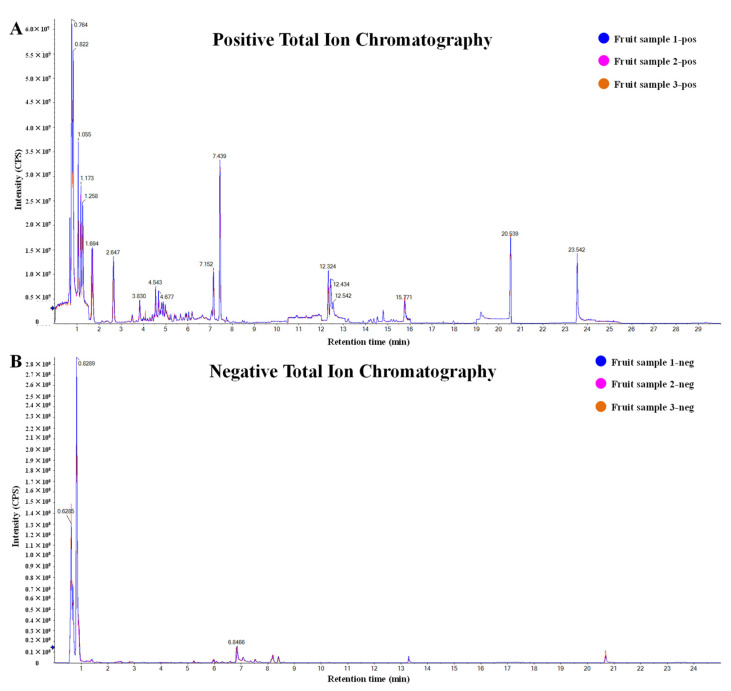
HPLC-MS/MS total ion chromatograms of extracts from *D. nobile* fruits. (**A**) Positive ion mode. (**B**) Negative ion mode.

The entire procedure of HPLC-MS/MS is shown in Figure S3. To identify some more metabolites in *D. nobile*, a positive mode with a BEH C8 chromatographic column and a negative mode with an HSS T3 chromatographic column were used for HPLC-MS/MS analysis for each sample. A total of 712 metabolites were finally identified in the methanol extracts of *D. nobile* by HPLC-MS/MS (Figure 4 and Figure S4). The detailed identification information of 55 metabolites is shown in Table 1. The detailed identification information of the remaining 657 metabolites is shown in Table S1. The partial extracted ion chromatograms screened by MRM are shown in Figure S5. Furthermore, quality control (QC) samples were used to evaluate the stability and reliability of HPLC-MS/MS (Figure S4). They were mixed samples of roots, stems, leaves, flowers, and fruits of *D. nobile* in this study. First, a good coincidence was obviously observed in the chromatography peaks among three repeated samples in both modes (Figure 4 and Figure S4). Then, Pearson correlation analysis further indicated the good consistence in repeated samples (coefficient > 0.98, Table 2). However, the coefficients between different types of samples were less than 0.8. These results suggested the good stability and reliability of HPLC-MS/MS, which is suitable for quantitative and comparative analysis.

### 3.5. Distribution of Metabolites in D. nobile Fruits by HPLC-MS/MS

**Figure 5:**
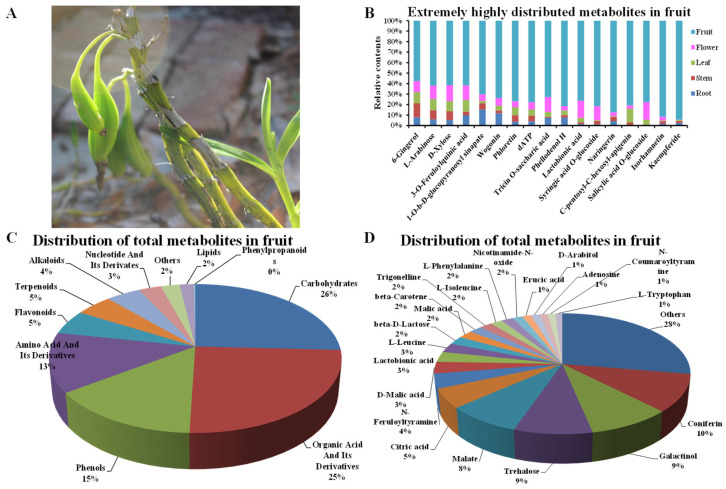
Distribution of the detected metabolites in *D. nobile* fruits. (**A**) Fruits of *D. nobile*. (**B**) Significantly highly accumulated metabolites in fruits compared to roots, stems, leaves, and flowers. Each of Log_2_(Fruit/Root), Log_2_(Fruit/Stem), Log_2_(Fruit/Leaf), Log_2_(Fruit/Flower) of the 17 components were more than 2 (*p* < 0.01). (**C**) The detected metabolites were classified into 11 kinds of chemical compounds (*n* = 712). (**D**) The proportion of each individual metabolite in the fruit.

As shown in Figure 5, the 712 metabolites were classified into 11 classes, including amino acids and their derivatives, flavonoids, organic acids and their derivatives, phenols, nucleotide and its derivatives, carbohydrates, lipids, terpenoids, alkaloids, phenylpropanoids, and others. The top four distributed classes were carbohydrates (25.76%), organic acids and their derivatives (24.79%), phenols (14.57%), and amino acid and its derivatives (12.96%) in fruits of *D. nobile* (Figure 5C). In detail, 21 metabolites showed more than 1% relative content in *D. nobile* fruits, such as coniferin, galactinol, trehalose, malate, and citric acid (Figure 5D). Moreover, there were 17 metabolites that showed a significant enrichment in fruits of *D. nobile* compared to root, stem, leaf, and flower (Log_2_(FC) > 2, *p* < 0.01), such as isorhamnetin, kaempferide, naringerin, L-arabinose, and D-xylose (Figure 5B). These results clearly display the distribution of main metabolites in *D. nobile* fruits by the identification and relative quantification based on HPLC-MS/MS.

### 3.6. Intracellular Antioxidant Activities Associated Metabolites

**Figure 6:**
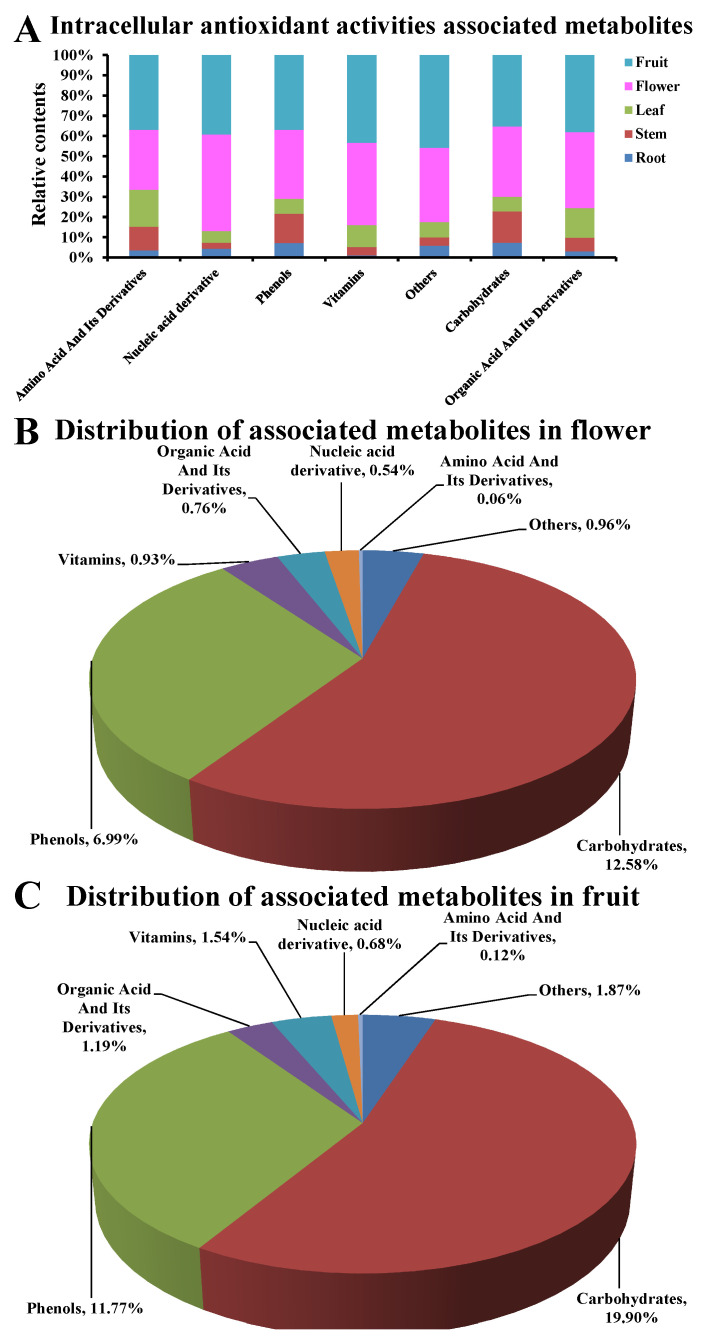
Distribution of metabolites associated with antioxidant activities in *D. nobile* flowers and fruits. (**A**) Proportion in flowers and fruits compared to roots, stems, and leaves. (**B**) Distribution of metabolites associated with antioxidant activities in flowers. (**C**) Distribution of metabolites associated with antioxidant activities in fruits. Metabolites associated with antioxidant activities were classified into seven types of chemical compounds (*n* = 55).

After correlation analysis, there were 55 metabolites that showed significant association (coefficient > 0.8, *p* < 0.05) with cell survival/suppressing rates, ROS levels, CAT activities, and SOD activities (Table 3). As shown in Figure 6, the 55 metabolites were mainly belonging to seven classes of phenols (*n* = 20), carbohydrates (*n* = 8), organic acid and its derivatives (*n* = 8), nucleic acid derivative (*n* = 5), amino acid and its derivatives (*n* = 4), vitamins (*n* = 4), and others (*n* = 6). All showed a higher proportion in flowers or fruits compared to that in roots, stems, and leaves (Figure 6A). However, carbohydrates (12.58% in flowers, 19.90% in fruits) and phenols (6.99% in flowers, 11.77% in fruits) showed a substantially higher distribution in flower/fruit compared to other classes of metabolites (<2% in flowers and fruits). These results indicate that some carbohydrates and phenol metabolites are significantly associated with intracellular antioxidant activities in *D. nobile* flowers and fruits.

### 3.7. The Main Intracellular Antioxidant Basis in Flowers and Fruits of D. nobile

**Figure 7:**
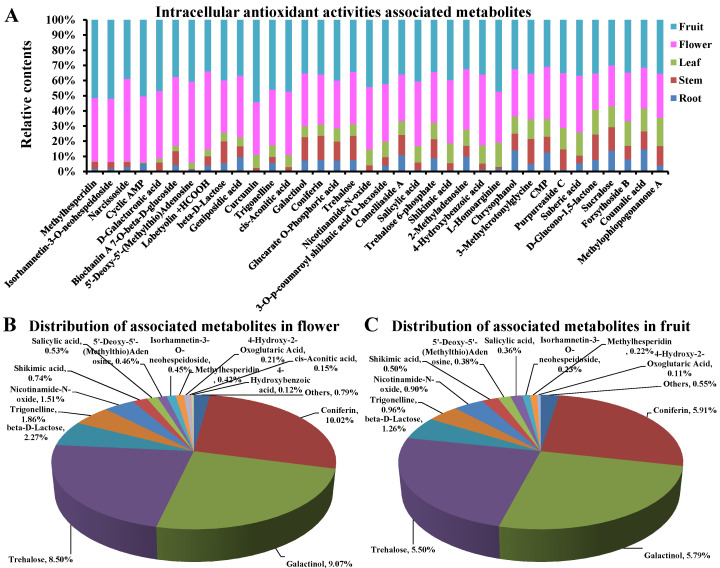
Distribution of each single metabolite associated to antioxidant activities in *D. nobile* flowers and fruits. (**A**) Proportion in flowers and fruits compared to roots, stems, and leaves. (**B**) Distribution of each metabolite associated with antioxidant activities in flowers. (**C**) Distribution of each single metabolite associated to antioxidant activities in fruits.

**Figure 8:**
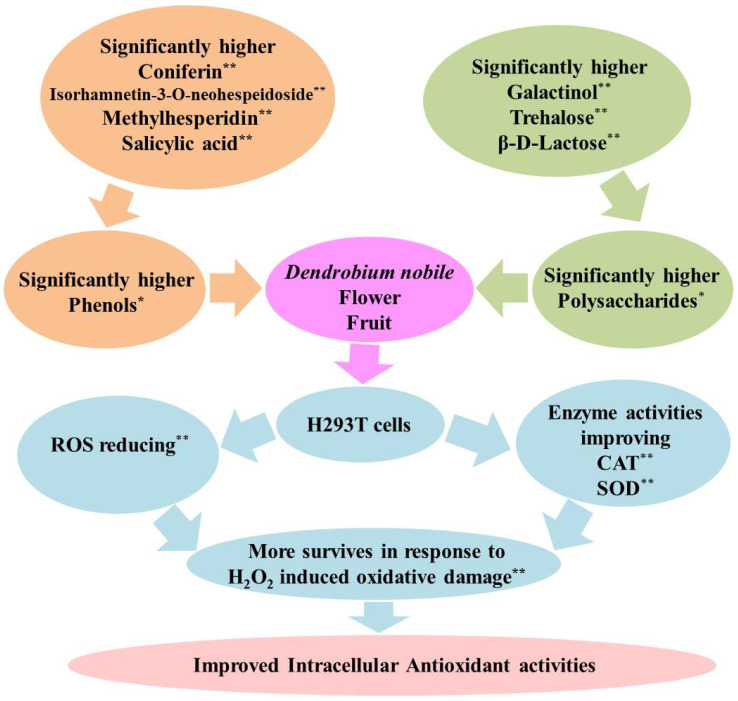
Diagram for the antioxidant basis in flowers and fruits of *D. nobile*. * *p* < 0.05, ** *p* < 0.01.

In detail, 36 of the above 55 metabolites showed a higher proportion in the flower/fruit compared to the root, stem, and leaf, such as methylhesperidin, isorhamnetin-3-O-neohespeidoside, narcissoside, galactinol, coniferin, and trehalose (Log_2_(FC) > 1, Figure 7A). Furthermore, only 13 of them showed a relatively high distribution in flowers or fruits, simultaneously (Log_2_(FC) > 1, distribution > 0.1%, Figure 7B,C). They are coniferin, galactinol, trehalose, beta-D-lactose, trigonelline, nicotinamide-N-oxide, shikimic acid, 5′-deoxy-5′-(methylthio)adenosine, salicylic acid, isorhamnetin-3-O-neohespeidoside, methylhesperidin, 4-hydroxybenzoic acid, and cis-aconitic acid. Among them, three carbohydrates, including galactinol, trehalose, and beta-D-lactose, showed proportions of 54.65% and 56.26% to the contents of all 13 metabolites in fruits and flowers, respectively. Five phenols, including coniferin, salicylic acid, isorhamnetin-3-O-neohespeidoside, methylhesperidin, and 4-hydroxybenzoic acid, showed proportions of 31.76% and 30.55% to the contents of the 13 metabolites in fruits and flowers, respectively. In particular, each of coniferin, galactinol, and trehalose counted for more than 5% of all the 712 compounds detected in fruits and flowers. Substantially, the accumulation of carbohydrates and phenols in flowers and fruits resulted in an improvement in CAT and SOD activities, a reduction in ROS levels, and an increase in survival in response to H_2_O_2_ stimulation (Figure 8). These results clearly display the main intracellular antioxidant basis in flowers and fruits of *D. nobile*.

### 3.8. Differences between In Vitro and Intracellular Antioxidants

**Figure 9:**
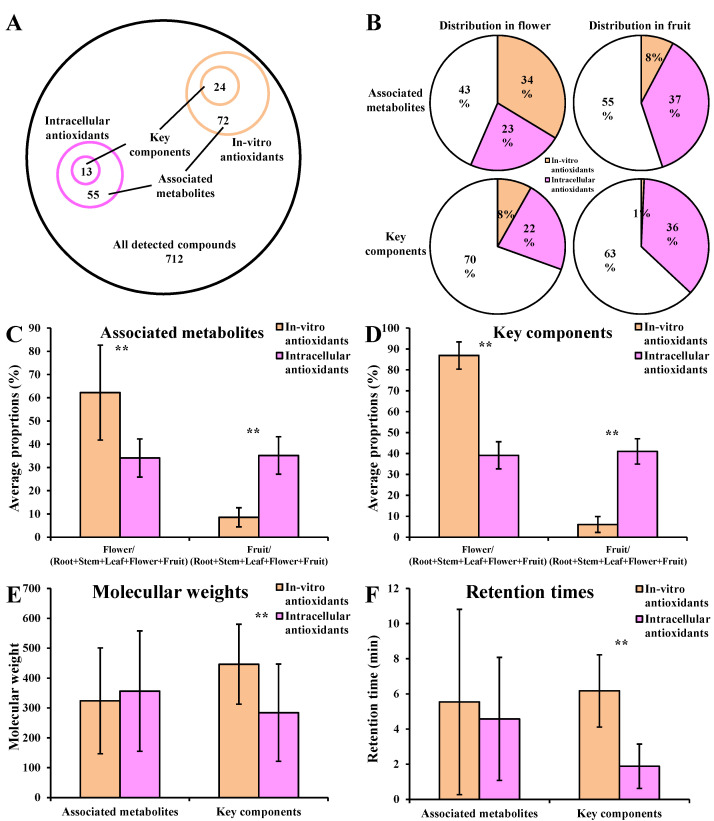
Comparison of in vitro and intracellular antioxidants in *D. nobile*. (**A**) Overview of in vitro and intracellular antioxidants. (**B**) Distribution of in vitro and intracellular antioxidants in flowers and fruits. (**C**) Proportion of in vitro and intracellular antioxidant activities associated metabolites in flowers or fruits to the sum of roots, stems, leaves, flowers, and fruits. (**D**) Proportion of key components for in vitro and intracellular antioxidant activities in flowers or fruits to the sum of roots, stems, leaves, flowers, and fruits. (**E**) Comparison of molecular weights. (**F**) Comparison of retention times. The in vitro antioxidants in *D. nobile* were reported previously [14]. ** *p* < 0.01.

There was no overlap between the in vitro antioxidants and intracellular antioxidants in *D. nobile* (Figure 9A). The in vitro and intracellular antioxidant activities associated metabolites (coefficient > 0.8, *p* < 0.05) showed about 50% of the relative concentration in flowers and fruits. The relative concentration of key components (Log_2_(FC) > 1, distribution > 0.1%) for intracellular antioxidant activities was obviously higher than those for in vitro antioxidant activities in both of flowers and fruits (Figure 9B). In vitro antioxidants were only significantly accumulated in *D. nobile* flowers. However, intracellular antioxidants were significantly accumulated in flowers and fruits of *D. nobile* (Figure 9C,D). Moreover, the average molecular weights of intracellular antioxidants were significantly lower than those of in vitro antioxidants, when compared as key components (*p* < 0.01, Figure 9E). The average retention times of intracellular antioxidants were also significantly lower than those of in vitro antioxidants, when compared as key components (*p* < 0.01, Figure 9F). These results suggest that the in vitro and intracellular antioxidants were completely different types of compounds with different characteristics.

### 3.9. Verification of the HPLC-MS/MS Results

**Figure 10:**
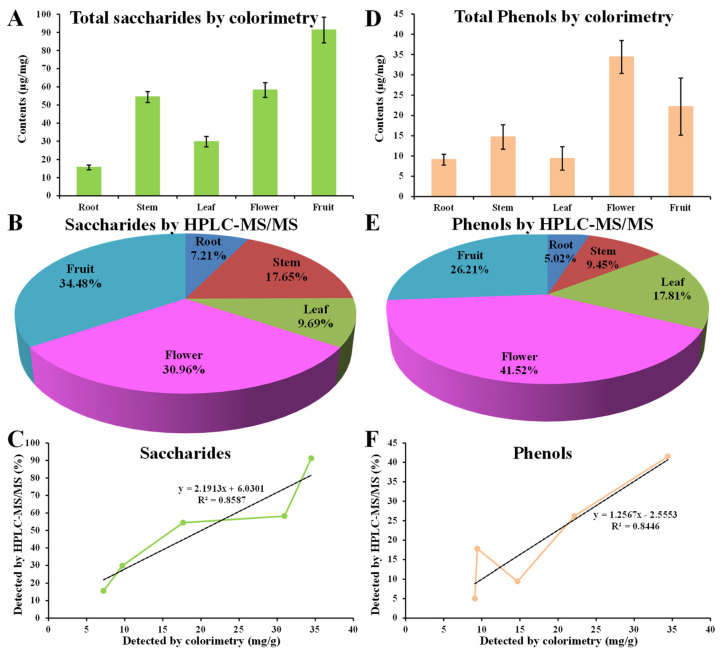
Comparison of the results detected by HPLC-MS/MS and the common determination method in *D. nobile*. (**A**) Total saccharides detected by colorimetry. (**B**) Sum of saccharides detected by HPLC-MS/MS (*n* = 62). (**C**) Linear analysis of saccharides contents by two methods. (**D**) Total phenols detected by colorimetry. (**E**) Sum of phenols detected by HPLC-MS/MS (*n* = 173). (**F**) Linear analysis of phenols contents by two methods.

The colorimetric method showed that the total saccharides contents in roots, stems, leaves, flowers, and fruits were 15.59 mg/g, 54.41 mg/g, 29.78 mg/g, 58.22 mg/g, and 91.28 mg/g, respectively (Figure 10A). Relative quantification by HPLC-MS/MS showed that the distributions of carbohydrates in roots, stems, leaves, flowers, and fruits were 7.21%, 17.65%, 9.69%, 30.96%, and 34.48%, respectively (Figure 10B). Linear analysis showed a good consistency in saccharide contents detected by the two methods (R^2^ > 0.8, Figure 10C). The colorimetric method showed that the total phenol contents in the roots, stems, leaves, flowers, and fruits were 9.09 mg/g, 14.68 mg/g, 9.41 mg/g, 34.40 mg/g, and 22.16 mg/g, respectively (Figure 10D). Relative quantification by HPLC-MS/MS showed that the distributions of total phenols in roots, stems, leaves, flowers, and fruits were 13.81%, 10.54%, 21.56%, 25.02%, and 29.06%, respectively (Figure 10E). Linear analysis also showed a good consistency in phenol contents detected by the two methods (R^2^ > 0.8, Figure 10F). Together with the previously observed consistence in amino acids and their derivatives, organic acid and its derivatives, and flavonoids [14], these results suggest the credibility of HPLC-MS/MS in the identification and relative quantification for metabolic analysis in *D. nobile*.

## 4. Discussions

### 4.1. Accumulation of Saccharides and Phenols Resulted in Fine Intracellular Antioxidant Activities in D. nobile Flowers and Fruits

The H_2_O_2_ induced oxidative model has been widely used for the evaluation of intracellular antioxidant activities in many types of cells [8,12,13]. In this model, the methanolic extracts from the flowers and fruits of *D. nobile* showed a good antioxidant capacity in reducing ROS, improving CAT and SOD, and surviving (Figure 1, Figure 2 and Figure 3). The metabolic analysis of HPLC-MS/MS clearly indicated the enrichment of amino acid and its derivatives, organic acids and their derivatives, carbohydrates, and phenols in flowers and fruits of *D. nobile* (Figure 5) [14]. This firstly gives a comprehensive and systematic insight into the secondary metabolism of *D. nobile* [14,16]. Then, the co-analysis of antioxidant activities and secondary metabolites revealed that some saccharide and phenol compounds played a key role in protecting cells from oxidative damage (Table 3, Figure 6 and Figure 7). The monosaccharides, oligosaccharides, and polysaccharides from medicinal plants had been widely reported to confer antioxidant activities by reversing ROS levels and restoring antioxidant enzyme activities [25,26]. Polysaccharides from *D. officinale* attenuated H_2_O_2_-induced oxidative stress in H9c2 cells by increasing SOD activities and inhibiting intracellular ROS [8]. Polysaccharides from *D. nobile* attenuated UVB-induced damage by regulating SOD and CAT activities and decreasing malondialdehyde level in the mice model [16]. Some phenolic compounds in the extracts of *D. catenatum*, *D. loddigesii*, *D. officinale*, and *D. nobile* had been reported in in vitro antioxidant actions, such as ABTS and DPPH scavenging [10,11,14]. Rich-polyphenols extract of *D. loddigesii* also showed the antioxidant abilities to reduce malondialdehyde level and increase SOD and CAT contents in mice [18]. These reports further confirmed the feasibility and accuracy of the metabolism-activity co-analysis method in this study. In a word, the accumulated saccharides and phenols in the flowers and fruits of *D. nobile* helped them in protecting H293T cells from H_2_O_2_-induced oxidative damage by increasing intracellular SOD and CAT activities and reducing intracellular ROS levels. Moreover, the accuracy of quasi-targeted metabolomics based on HPLC-MS/MS was verified on flavonoid content with rutin standard, total protein content with BSA standard, total organic acid content with citric acid standard, total phenol content with gallic acid monohydrate standard, and saccharide content with glucose standard [14].

### 4.2. Intracellular Antioxidants Showed Different Characteristics from In Vitro Antioxidants in D. nobile

Previously, some flavonoids, organic acids and their derivatives, and amino acids and their derivatives were identified as key compounds involved in the in vitro antioxidant activities of ferric-reducing and ABTS- and DPPH-scavenging in *D. nobile*, such as rutin, astragalin, isomucronulatol-7-O-glucoside, quercetin 4′-O-glucoside, methylquercetin O-hexoside, caffeic acid, caffeic acid O-glucoside, and p-coumaric acid [14]. Here, some of the saccharides and phenols were identified as the key compounds involved in intracellular antioxidant activities in *D. nobile*, such as coniferin, salicylic acid, isorhamnetin-3-O-neohespeidoside, methylhesperidin, 4-hydroxybenzoic acid, galactinol, trehalose, and beta-D-lactose (Figure 8). Firstly, they were completely different compounds, and they showed different distributions in roots, stems, leaves, flowers, and fruits of *D. nobile*. Second, intracellular antioxidants showed lower molecular weights than in vitro antioxidants. This is consistent with the fact that small-molecule plant secondary metabolites have been reported to show superiority in intracellular activities. For example, small-molecule procyanidin B1 significantly reduced ROS levels in response to H_2_O_2_ accumulation in mouse somatic cell nuclear transfer embryos [27]. Small-molecule flavonoids from *Sorbus pohuashanensis* were related to antitumor activity [28]. Thirdly, the intracellular antioxidants showed lower retention times than the in vitro antioxidants. Under the chromatographic conditions in this study, lower retention time means larger polarity for a component. The polarity was important for functional performing of plant extracts. For example, the polar character of the phenolic components significantly influenced their antioxidant capacity and biological activities [29]. The polarity of the metabolites in *D. nobile* was also reported to be related to intracellular activities. Less-polar components in ethanol extracts of *D. nobile* showed strong suppressing efficacy to A549 lung cancer cells [19]. Weak polar compounds of the ether extract exhibited a strong anticancer effect in HepG2 liver cancer cells [23]. In conclusion, the newly identified intracellular antioxidants showed significantly lower molecular weight and larger polarity than previously identified in vitro antioxidants in *D. nobile*. The different characteristics of intracellular antioxidants could provide new information about the development of antioxidant products from medicinal plants.

### 4.3. The Newly Identified Intracellular Antioxidants Will Further Enrich the Prospects of Dendrobium on Pharmaceuticals and Health-Care Products

The extracts of *Dendrobium* species have been reported to show good antioxidant ability in many assays [8,9,10,11,12,13,14,15,16]. However, effective antioxidants were poorly identified in *Dendrobium*, especially for intracellular antioxidant activities [14,17,22]. In this paper, five phenolic compounds, three oligosaccharides, and five other metabolites were newly identified as key intracellular antioxidants in protecting H293T cells from oxidative damage. For the five phenolic compounds, only 4-hydroxybenzoic acid had been reported to associate with recovering of antioxidant enzymes and decreasing of oxidative stress in porcine kidney cells PK15 in the extracts of *D. nobile* [30]. The rest of them were poorly reported as intracellular antioxidants in *Dendrobium* and some other medicinal plants. Coniferin and its derivative from *Linum usitatissimum* have recently been reported to be involved in antioxidant activity in the β-carotene-linoleic acid emulsion system [24]. For the three oligosaccharides, galactinol, trehalose, and beta-D-lactose had not been reported as intracellular antioxidants in *Dendrobium* in previous studies. However, beta-D-lactose has been reported as a bioactive constituent in the extract of a standardized herbal cocktail that modulates oxidative stress in mouse models of major depression and post-traumatic stress disorder [25]. Trehalose has also been reported to alleviate oxidative stress in peripheral blood mononuclear cells stimulated by lipopolysaccharides [26]. This means that some oligosaccharides were still important for the antioxidant activities of *Dendrobium* extracts but not only polysaccharides. The five other metabolites, trigonelline, nicotinamide-N-oxide, shikimic acid, 5′-deoxy-5′-(methylthio)adenosine, and cis-aconitic acid were also poorly reported in *Dendrobium* for antioxidant activities. Trigonelline significantly alleviated UV-B-induced cell death effects in primary human dermal fibroblasts [31]. Shikimic acid from *Artemisia absinthium* enhanced antioxidant activity in diabetic rats [32]. cis-Aconitic acid was an important antioxidant constituent of *Echinodorus grandiflorus* for inhibiting antigen-induced arthritis and monosodium urate-induced arthritis in mice [33]. Substantially, almost all of the 13 compounds had not been reported for antioxidant activities in *Dendrobium* before this study. They enriched the library of safe and effective antioxidants, which was helpful for the utilization of *Dendrobium* in pharmaceuticals and health-care products. The following studies will focus on the targeted identification, isolation, bioactivities, and biosynthesis of these key metabolites.

## Figures and Tables

**Table 1:** Detailed information of 55 metabolites identified by HPLC-MS/MS in *D. nobile* ^1^.

Compound ID	Name	Formula	MW (Da)	RT (min)	Q1 (*m*/*z*)	Class	Peak Areas								
							Flower1	Flower2	Flower3	Fruit1	Fruit2	Fruit3	QC1	QC2	QC3
Com_405_pos	(−)-trans-Carveol	C_10_H_16_O	152.233	1.09	153.13	Lipids	124,600	107,900	92,890	103,500	121,200	90,420	89,580	82,780	101,400
Com_245_neg	(+)-Dihydrojasmonic acid	C_12_H_20_O_3_	212.285	13.03	211.10	Organic Acid And Its Derivatives	36,260	26,710	22,320	27,540	36,160	30,830	28,420	20,820	17,560
Com_131_neg	13-HOTrE	C_18_H_30_O_3_	294.000	12.70	293.00	Lipids	24,070	22,970	32,880	26,400	29,030	28,130	18,910	20,200	25,650
Com_558_pos	2-Methyladenosine	C_11_H_15_N_5_O_4_	281.268	0.77	282.10	Nucleotide And Its Derivates	4,340,000	1,754,000	1,430,000	2,165,000	1,921,000	2,051,000	1,351,000	1,199,000	1,256,000
Com_437_pos	3-Hydroxy-3-methylpentane-1,5-dioic acid	C_6_H_10_O_5_	162.141	4.23	163.00	Amino Acid And Its Derivatives	1,626,000	683,500	1,504,000	1,670,000	1,635,000	1,518,000	508,700	453,400	506,600
Com_375_pos	3-Methylcrotonylglycine	C_7_H_11_NO_3_	157.167	0.61	158.10	Organic Acid And Its Derivatives	154,600	131,500	110,400	154,400	150,700	159,300	120,400	94,650	67,130
Com_271_neg	3-O-p-Coumaroyl shikimic acid O-hexoside	C_22_H_26_O_12_	482.100	5.30	481.10	Organic Acid And Its Derivatives	244,000	33,630	7233	110,400	104,900	104,000	44,740	43,480	43,210
Com_247_neg	4-Hydroxy-2-oxoglutaric acid	C_5_H_6_O_6_	162.098	0.68	161.00	Organic Acid And Its Derivatives	5,392,000	3,086,000	3,504,000	4,633,000	5,496,000	4,981,000	4,075,000	3,752,000	3,866,000
Com_195_neg	4-Hydroxybenzoic acid	C_7_H_6_O_3_	138.121	0.78	137.02	Phenols	4,686,000	3,329,000	2,630,000	2,804,000	2,826,000	2,589,000	1,798,000	1,737,000	1,761,000
Com_647_pos	4-Nitrophenol	C_6_H_5_NO_3_	139.109	29.43	140.00	Phenols	1,210,000	1,138,000	1,205,000	1,245,000	1,166,000	1,228,000	698,600	1,059,000	1,281,000
Com_485_pos	5′-Deoxy-5′-(methylthio)adenosine	C_11_H_15_N_5_O_3_S	297.333	2.57	298.00	Nucleotide And Its Derivates	2,370,000	22,660,000	16,880,000	11,170,000	10,400,000	10,500,000	4,789,000	4,347,000	4,120,000
Com_300_neg	beta-D-Lactose	C_12_H_22_O_11_	342.297	0.74	341.11	Carbohydrates	37,570,000	44,770,000	54,900,000	54,530,000	52,990,000	51,770,000	31,150,000	33,200,000	35,770,000
Com_487_pos	Biochanin A 7-O-beta-D-glucoside	C_22_H_22_O_10_	446.404	5.46	447.20	Flavonoids	1,057,000	74,550	72,300	325,800	352,000	323,100	132,500	220,300	177,900
Com_368_pos	Biotin	C_10_H_16_N_2_O_3_S	244.311	9.67	245.10	Terpenoids	563,500	363,600	393,000	402,600	388,600	456,800	274,300	390,400	416,400
Com_728_pos	Camelliaside A	C_33_H_40_O_20_	756.668	4.21	757.22	Flavonoids	47,800	67,980	40,690	55,470	50,270	77,070	14,590	23,090	15,770
Com_417_pos	Chrysophanol	C_15_H_10_O_4_	254.238	8.17	255.06	Phenols	131,998	22,440	6,259	56,540	44,750	66,440	45,470	53,540	51,910
Com_178_neg	cis-Aconitic acid	C_6_H_6_O_6_	174.108	0.60	173.00	Organic Acid And Its Derivatives	4,248,000	2,009,000	3,113,000	3,624,000	3,708,000	3,272,000	1,294,000	1,589,000	1,520,000
Com_672_pos	Cytidine monophosphate	C_9_H_14_N_3_O_8_P	323.197	0.74	324.00	Nucleotide And Its Derivates	246,900	253,000	226,900	218,300	227,000	206,500	108,300	126,100	124,600
Com_253_neg	Coniferin	C_16_H_22_O_8_	342.341	0.87	341.00	Phenols	169,400,000	223,900,000	251,000,000	240,200,000	223,600,000	240,500,000	149,200,000	160,000,000	170,700,000
Com_277_neg	Coumalic acid	C_6_H_4_O_4_	140.094	1.15	141.02	Organic Acid And Its Derivatives	98,560	90,310	69,600	77,940	114,800	110,700	56,040	70,340	62,880
Com_32_neg	Curcumin	C_21_H_20_O_6_	368.380	4.53	367.12	Phenols	118,100	49,710	60,470	126,100	108,100	120,400	30,700	36,390	33,940
Com_134_neg	Cyclic adenosine monophosphate	C_10_H_12_N_5_O_6_P	329.206	3.60	328.10	Nucleotide And Its Derivates	478,100	2,589,000	2,549,000	2,181,000	2,054,000	2,283,000	870,500	880,800	980,400
Com_29_neg	D-Galacturonic acid	C_6_H_10_O_7_	194.139	0.71	193.04	Carbohydrates	137,000	118,200	342,900	180,000	226,200	225,000	92,630	87,140	103,400
Com_27_neg	D-Glucono-1,5-lactone	C_6_H_10_O_6_	178.140	0.80	177.14	Amino Acid And Its Derivatives	254,700	128,600	83,170	230,200	222,400	235,300	136,800	166,100	145,300
Com_4_neg	Echinacoside	C_35_H_46_O_20_	786.737	7.70	685.22	Phenols	275,200	311,500	448,500	365,900	334,300	344,500	171,900	154,800	117,000
Com_194_neg	Forsythoside B	C_34_H_44_O_19_	756.702	7.52	755.24	Phenylpropanoids	446,900	549,000	325,000	486,400	465,100	469,100	183,700	190,800	194,700
Com_238_neg	Galactinol	C_12_H_22_O_11_	342.116	0.87	341.10	Carbohydrates	192,900,000	203,400,000	234,800,000	208,300,000	191,200,000	238,000,000	151,100,000	148,800,000	156,700,000
Com_79_neg	Geniposidic acid	C_16_H_22_O_10_	374.340	0.75	373.11	Terpenoids	104,700	43,100	36,340	65,210	45,800	56,160	33,990	36,810	28,020
Com_273_neg	Glucarate O-phosphoric acid	C_6_H_11_PO_11_	290.100	0.63	289.10	Carbohydrates	280,000	47,750	48,560	164,600	145,500	167,800	79,100	80,190	102,700
Com_707_pos	Homovanillic Acid	C_9_H_10_O_4_	182.173	9.37	183.10	Organic Acid And Its Derivatives	90,000	5,783	4,315	25,880	24,000	24,810	12,050	14,070	18,100
Com_588_pos	iP9G	C_16_H_23_N_5_O_5_	365.384	0.77	366.20	Others	810,100	906,900	1,022,000	911,800	893,000	955,300	651,900	691,700	753,500
Com_111_neg	Isorhamnetin-3-O-neohespeidoside	C_28_H_32_O_16_	624.544	7.93	623.16	Flavonoids	4,094,000	10,180,000	10,700,000	10,560,000	9,969,000	10,810,000	3,783,000	4,058,000	4,088,000
Com_213_neg	Jionoside A1	C_36_H_48_O_20_	800.760	8.14	799.27	Others	50,711	14,330	17,309	40,330	32,340	45,130	11,840	11,080	9100
Com_522_pos	L-Homoarginine	C_7_H_16_N_4_O_2_	188.227	2.59	189.00	Amino Acid And Its Derivatives	176,900	833,600	723,000	888,500	785,800	780,400	332,100	317,700	304,100
Com_182_neg	L-Homocitrulline	C_7_H_15_N_3_O_3_	189.212	0.82	188.10	Amino Acid And Its Derivatives	9204	7947	6433	8819	6806	10,430	7105	7172	3215
Com_164_neg	Lobetyolin +HCOOH	C_21_H_30_O_10_	442.460	0.81	441.18	Others	291,200	16,640	18,360	73,710	70,290	71,450	65,130	61,160	34,790
Com_301_neg	LysoPA 18:0	C_21_H_35_O_7_P	438.270	0.80	437.27	Lipids	25,280	19,130	29,190	27,660	22,070	23,370	23,600	21,710	17,100
Com_320_neg	Methylhesperidin	C_29_H_36_O_15_	624.587	7.95	623.20	Flavonoids	3,760,000	9,394,000	10,600,000	10,530,000	9,403,000	9,479,000	3,518,000	3,835,000	3,961,000
Com_47_neg	Methylophiopogonanone A	C_19_H_18_O_6_	342.343	0.76	341.10	Flavonoids	39,480	67,950	78,550	75,730	75,180	75,100	49,290	35,960	53,470
Com_103_neg	N,N-Dimethylglycine	C_4_H_9_NO_2_	103.120	0.64	102.06	Amino Acid And Its Derivatives	754,700	222,600	124,000	297,800	361,300	377,700	257,600	287,000	124,000
Com_600_pos	Narcissoside	C_28_H_32_O_16_	624.544	5.92	625.18	Flavonoids	1,213,000	3,106,000	4,647,000	2,422,000	1,918,000	2,037,000	983,100	1,251,000	1,191,000
Com_51_neg	Nicotinamide-N-oxide	C_6_H_6_N_2_O_2_	138.043	0.70	137.00	Terpenoids	32,870,000	33,780,000	31,940,000	34,720,000	38,290,000	33,330,000	20,920,000	18,380,000	19,710,000
Com_446_pos	Nicotinic acid-hexoside			1.09	286.00	Phenols	144,700	104,500	130,100	120,400	104,000	84,530	65,870	67,680	70,900
Com_171_neg	Palmitaldehyde	C_16_H_32_O	240.425	14.03	239.00	Organic Acid And Its Derivatives	63,100	32,180	16,110	33,370	51,830	32,360	46,760	35,500	19,610
Com_460_pos	Palmitic acid	C_16_H_32_O_2_	256.424	18.32	257.25	Lipids	17,060	17,170	13,510	15,500	15,020	17,780	13,480	15,510	15,230
Com_304_neg	Purpureaside C	C_35_H_46_O_20_	786.728	7.61	785.25	Phenylpropanoids	242,900	239,600	345,200	275,900	278,200	239,000	120,500	112,200	109,800
Com_196_neg	Salicylic acid	C_7_H_6_O_3_	138.121	0.67	137.10	Organic Acid And Its Derivatives	18,580,000	9,875,000	10,580,000	12,990,000	12,950,000	11,250,000	7,783,000	7,140,000	7,478,000
Com_218_neg	Shikimic acid	C_7_H_10_O_5_	174.151	0.57	173.05	Organic Acid And Its Derivatives	23,910,000	15,040,000	15,710,000	16,810,000	17,570,000	17,540,000	11,030,000	10,600,000	10,850,000
Com_65_neg	Suberic acid	C_8_H_14_O_4_	174.194	0.63	173.10	Organic Acid And Its Derivatives	2,458,000	1,542,000	1,552,000	1,737,000	1,788,000	1,937,000	1,051,000	1,212,000	1,131,000
Com_311_neg	Sucralose	C_12_H_19_C_l3_O_8_	397.634	0.63	395.01	Carbohydrates	317,600	314,900	204,600	294,400	315,100	316,600	250,000	225,000	195,300
Com_38_neg	trans-Ferulic acid	C_10_H_10_O_4_	194.184	2.87	193.00	Phenols	628,200	94,470	155,500	348,700	242,900	271,700	295,000	166,100	138,000
Com_262_neg	Trehalose	C_12_H_22_O_11_	342.297	0.74	341.11	Carbohydrates	175,400,000	207,700,000	216,200,000	209,200,000	192,200,000	196,300,000	137,600,000	147,400,000	162,400,000
Com_263_neg	Trehalose 6-phosphate	C_12_H_23_O_14_P	422.276	0.51	421.08	Carbohydrates	740,600	528,700	634,900	673,200	668,300	615,900	328,800	325,700	397,700
Com_340_pos	Trigonelline	C_7_H_7_NO_2_	137.136	0.73	138.06	Alkaloids	38,200,000	29,560,000	36,390,000	45,160,000	43,450,000	41,950,000	17,400,000	17,590,000	17,930,000
Com_268_neg	Vitamin D3	C_27_H_44_O	384.339	0.75	383.00	Terpenoids	576,800	175,500	249,600	235,800	227,100	242,000	145,600	209,500	229,500

^1^ MW molecular weight. RT retention time. Q1 parent ion. QC quality control.

**Table 2:** Pearson correlation among different samples detected by HPLC-MS/MS.

Names	Flower1	Flower2	Flower3	Fruit1	Fruit2	Fruit3	QC1	QC2	QC3
Flower1	1.000	0.985 **	0.983 **	0.729	0.724	0.729	0.539	0.556	0.562
Flower2	0.985 **	1.000	0.986 **	0.711	0.710	0.713	0.542	0.559	0.567
Flower3	0.983 **	0.986 **	1.000	0.665	0.667	0.668	0.542	0.561	0.568
Fruit1	0.729	0.711	0.665	1.000	0.987 **	0.986 **	0.543	0.570	0.542
Fruit2	0.724	0.710	0.667	0.987 **	1.000	0.986 **	0.550	0.577	0.543
Fruit3	0.729	0.713	0.668	0.986 **	0.986 **	1.000	0.547	0.570	0.538
QC1	0.539	0.542	0.542	0.543	0.550	0.547	1.000	0.982 **	0.986 **
QC2	0.556	0.559	0.561	0.570	0.577	0.570	0.982 **	1.000	0.989 **
QC3	0.562	0.567	0.568	0.542	0.543	0.538	0.986 **	0.989 **	1.000

** *p* < 0.01.

**Table 3:** Correlation of intracellular antioxidant indexes to metabolites in *D. nobile* ^1^.

NO.	Name	Cell Survival Rate		Cell Suppressing Rate		ROS1	ROS2	CAT1	CAT2	SOD1	SOD2
		50	100	50	100						
1	(-)-*trans*-Carveol	0.996 **	0.940 *	−0.984 **	−0.982 **	−0.980 **	−0.929 *	0.976 **	−0.897 *	0.993 **	−0.916 *
2	(+)-Dihydrojasmonic acid	0.913 *	0.861	−0.839	−0.817	−0.871	−0.816	0.933 *	−0.899 *	0.921 *	−0.877
3	13-HOTrE	0.976 **	0.932 *	−0.975 **	−0.935 *	−0.999 **	−0.990 **	0.921 *	−0.892 *	0.938 *	−0.899 *
4	2-Methyladenosine	0.876	0.816	−0.935 *	−0.892 *	−0.927 *	−0.937*	0.789	−0.714	0.855	−0.752
5	3-Hydroxy-3-methylpentane- 1,5-dioic acid	0.966 **	0.965 **	−0.924 *	−0.923 *	−0.967 **	−0.935 *	0.937 *	−0.964 **	0.943 *	−0.952 *
6	3-Methylcrotonylglycine	0.895 *	0.981 **	−0.883 *	−0.922 *	−0.945 *	−0.941 *	0.817	−0.949 *	0.898 *	−0.882 *
7	3-O-p-coumaroyl shikimic- acid O-hexoside	0.924 *	0.906 *	−0.946 *	−0.904 *	−0.978 **	−0.995 **	0.839	−0.848	0.885 *	−0.824
8	4-Hydroxy-2-oxoglutaric acid	0.955 *	0.930 *	−0.899 *	−0.888 *	−0.937 *	−0.895 *	0.947 *	−0.946 *	0.926 *	−0.950 *
9	4-Hydroxybenzoic acid	0.937 *	0.873	−0.975 **	−0.952 *	−0.960 **	−0.943 *	0.874	−0.782	0.929*	−0.847
10	4-Nitrophenol	0.973 **	0.952 *	−0.926 *	−0.953 *	−0.940 *	−0.873	0.975 **	−0.944 *	0.979 **	−0.894 *
11	5′-Deoxy-5′-(methylthio)- adenosine	0.883 *	0.837	−0.937 *	−0.907 *	−0.932 *	−0.937 *	0.796	−0.736	0.869	−0.772
12	beta-D-Lactose	0.827	0.917 *	−0.845	−0.864	−0.910 *	−0.938 *	0.717	−0.863	0.818	−0.772
13	Biochanin A-7-O-beta- D-glucoside	0.846	0.863	−0.896 *	−0.899 *	−0.912 *	−0.924 *	0.746	−0.768	0.847	−0.761
14	Biotin	0.938 *	0.867	−0.934 *	−0.974 **	−0.889 *	−0.800	0.945 *	−0.801	0.978 **	−0.945 *
15	Camelliaside A	0.822	0.877	−0.851	−0.832	−0.912 *	−0.955 *	0.705	−0.819	0.790	−0.728
16	Chrysophanol	0.832	0.815	−0.884 *	−0.823	−0.916 *	−0.961 **	0.719	−0.734	0.784	−0.693
17	cis-Aconitic acid	0.923 *	0.909 *	−0.945 *	−0.906 *	−0.978 **	−0.994 **	0.838	−0.851	0.886 *	−0.826
18	Cytidylic acid	0.856	0.818	−0.915 *	−0.870	−0.922 *	−0.946 *	0.757	−0.720	0.828	−0.728
19	Coniferin	0.816	0.912 *	−0.837	−0.866	−0.899 *	−0.924 *	0.705	−0.853	0.815	−0.766
20	Coumalic acid	0.870	0.836	−0.900 *	−0.817	−0.943 *	−0.987 **	0.772	−0.784	0.801	−0.733
21	Curcumin	0.891 *	0.888 *	−0.894 *	−0.833	−0.955 *	−0.988 **	0.803	−0.861	0.827	−0.789
22	Cyclic adenylic acid	0.839	0.834	−0.882 *	−0.825	−0.924 *	−0.970 **	0.726	−0.763	0.789	−0.708
23	D-Galacturonic acid	0.880 *	0.900 *	−0.911 *	−0.895 *	−0.949 *	−0.971 **	0.779	−0.831	0.858	−0.790
24	D-Glucono-1,5-lactone	0.863	0.954 *	−0.820	−0.837	−0.910 *	−0.916 *	0.797	−0.968 **	0.837	−0.857
25	Echinacoside	0.989 **	0.966 **	−0.969 **	−0.982 **	−0.980 **	−0.931 *	0.965 **	−0.932 *	0.991 **	−0.975 **
26	Ferulic acid	0.984 **	0.890 *	−0.996 **	−0.942 *	−0.991 **	−0.971 **	0.941 *	−0.833	0.948 *	−0.892 *
27	Forsythoside B	0.962 **	0.908 *	−0.976 **	−0.924 *	−0.994 **	−0.995 **	0.897 *	−0.855	0.920 *	−0.865
28	Galactinol	0.825	0.908 *	−0.853	−0.883 *	−0.903 *	−0.922 *	0.717	−0.838	0.831	−0.772
29	Geniposidic acid	0.834	0.819	−0.893*	−0.855	−0.910 *	−0.941 *	0.726	−0.724	0.808	−0.710
30	Glucarate O-phosphoric acid	0.853	0.912 *	−0.866	−0.853	−0.933 *	−0.967 **	0.748	−0.869	0.821	−0.777
31	Homovanillic acid	0.966 **	0.867	−0.978 **	−0.975 **	−0.937 *	−0.874	0.954 *	−0.793	0.978 **	−0.923 *
32	N6-Isopentenyl adenine- 9-glucoside	0.849	0.906 *	−0.880 *	−0.891 *	−0.924 *	−0.944 *	0.744	−0.834	0.844	−0.780
33	Isorhamnetin-3-O- neohespeidoside	0.845	0.878	−0.874	−0.841	−0.930 *	−0.971 **	0.734	−0.821	0.806	−0.743
34	Jionoside A1	0.946 *	0.893 *	−0.917 *	−0.852	−0.960 **	−0.956 *	0.909 *	−0.894 *	0.882 *	−0.875
35	L-Homoarginine	0.942 *	0.885 *	−0.938 *	−0.862	−0.976 **	−0.989 **	0.881 *	−0.861	0.874	−0.839
36	L-Homocitrulline	0.845	0.986 **	−0.803	−0.910 *	−0.869	−0.831	0.794	−0.975 **	0.889 *	−0.911 *
37	Lobetyolin +HCOOH	0.837	0.810	−0.900 *	−0.897 *	−0.885 *	−0.884 *	0.749	−0.696	0.846	−0.743
38	LysoPA 18:0	0.957 *	0.941 *	−0.907 *	−0.956 *	−0.911 *	−0.828	0.969 **	−0.930 *	0.981 **	−0.856
39	Methylhesperidin	0.846	0.880 *	−0.875	−0.845	−0.930 *	−0.970 **	0.735	−0.821	0.808	−0.745
40	Methylophiopogonanone A	0.971 **	0.971 **	−0.943 *	−0.940 *	−0.983 **	−0.958 *	0.931 *	−0.957 *	0.950 *	−0.945 *
41	N,N-Dimethylglycine	0.866	0.975 **	−0.850	−0.952 *	−0.889 *	−0.847	0.811	−0.932 *	0.919 *	−0.910 *
42	Narcissoside	0.832	0.812	−0.896 *	−0.883 *	−0.892 *	−0.903 *	0.735	−0.702	0.830	−0.727
43	Nicotinamide-N-oxide	0.944 *	0.914 *	−0.963 **	−0.925 *	−0.988 **	−0.994 **	0.868	−0.856	0.910 *	−0.850
44	Nicotinic acid-hexoside	0.971 **	0.836	−0.969 **	−0.946 *	−0.922 *	−0.849	0.982 **	−0.779	0.970 **	−0.929 *
45	Palmitaldehyde	0.962 **	0.992 **	−0.935 *	−0.966 **	−0.973 **	−0.938 *	0.920 *	−0.968 **	0.965 **	−0.901 *
46	Palmitic acid	0.971 **	0.952 *	−0.974 **	−0.959 **	−0.997 **	−0.983 **	0.912 *	−0.903 *	0.951 *	−0.910 *
47	Purpureaside C	0.949 *	0.974 **	−0.948 *	−0.982 **	−0.971 **	−0.942 *	0.892 *	−0.921 *	0.962 **	−0.929 *
48	Salicylic acid	0.944 *	0.912 *	−0.970 **	−0.944 *	−0.983 **	−0.980 **	0.871	−0.841	0.923 *	−0.856
49	Shikimic acid	0.960 **	0.913 *	−0.982 **	−0.951 *	−0.989 **	−0.980 **	0.896 *	−0.845	0.938 *	−0.874
50	Suberic acid	0.961 **	0.887 *	−0.985 **	−0.931 *	−0.989 **	−0.985 **	0.898 *	−0.821	0.923 *	−0.856
51	Sucralose	0.857	0.898 *	−0.882 *	−0.863	−0.937 *	−0.970 **	0.749	−0.841	0.827	−0.768
52	Trehalose	0.823	0.910 *	−0.850	−0.886 *	−0.899 *	−0.916 *	0.716	−0.839	0.832	−0.775
53	Trehalose 6-phosphate	0.884 *	0.913 *	−0.913 *	−0.906 *	−0.951 *	−0.968 **	0.787	−0.844	0.869	−0.805
54	Trigonelline	0.880 *	0.871	−0.906 *	−0.848	−0.953 *	−0.990 **	0.780	−0.818	0.826	−0.762
55	Vitamin D3	0.841	0.866	−0.881 *	−0.938 *	−0.872	−0.842	0.769	−0.763	0.886 *	−0.811

^1^ 50 and 100 indicate the concentration of *D. nobile* extracts. ROS1 ROS accumulation, ROS2 ROS increasing, CAT1 CAT activities, CAT2 suppressed CAT activities, SOD1 SOD activities, SOD2 suppressed SOD activities. * *p* < 0.05, ** *p* < 0.01.

## Data Availability

The data presented in this study are available in the article and Supplementary Materials.

## Supplementary Materials

The following supporting information can be downloaded at: https://www.mdpi.com/article/10.3390/metabo13060702/s1, Table S1: Detailed information of 657 metabolites identified by HPLC-MS/MS in *D. nobile*. Figure S1: The survival rates of H293T cells under different concentrations of H_2_O_2_. Figure S2: Standard curves used for quantification of total proteins, total soluble saccharides, total phenols, and CAT activities. Figure S3: Schematic diagram for the entire experimental procedure in this study. Figure S4: HPLC-MS/MS total ion chromatograms of QC samples. Figure S5: Partial extracted ion chromatograms screened by MRM.

## References

[1] Forman H.J., Zhang H. (2021). Targeting oxidative stress in disease: Promise and limitations of antioxidant therapy. Nat. Rev. Drug Discov..

[2] Pisoschi A.M., Pop A. (2015). The role of antioxidants in the chemistry of oxidative stress: A review. Eur. J. Med. Chem..

[3] Xu D.P., Li Y., Meng X., Zhou T., Zhou Y., Zheng J., Zhang J.J., Li H.B. (2017). Natural antioxidants in foods and medicinal plants: Extraction, assessment and resources. Int. J. Mol. Sci..

[4] Zafar F., Asif H.M., Shaheen G., Ghauri A.O., Rajpoot S.R., Tasleem M.W., Shamim T., Hadi F., Noor R., Ali T. (2023). A comprehensive review on medicinal plants possessing antioxidant potential. Clin. Exp. Pharmacol. Physiol..

[5] Periera da Silva A., Rocha R., Silva C.M., Mira L., Duarte M.F., Florêncio M.H. (2000). Antioxidants in medicinal plant extracts. A research study of the antioxidant capacity of *Crataegus*, *Hamamelis* and *Hydrastis*. Phytother. Res..

[6] Inayatullah S., Prenzler P.D., Obied H.K., Rehman A.U., Mirza B. (2012). Bioprospecting traditional Pakistani medicinal plants for potent antioxidants. Food Chem..

[7] Swain S., Rautray T.R. (2021). Estimation of trace elements, antioxidants, and antibacterial agents of regularly consumed indian medicinal plants. Biol. Trace Elem. Res..

[8] Zhao X., Dou M., Zhang Z., Zhang D., Huang C. (2017). Protective effect of *Dendrobium officinale* polysaccharides on H_2_O_2_-induced injury in H9c2 cardiomyocytes. Biomed. Pharmacother..

[9] Chan C.F., Wu C.T., Huang W.Y., Lin W.S., Wu H.W., Huang T.K., Chang M.Y., Lin Y.S. (2018). Antioxidation and melanogenesis inhibition of various *Dendrobium tosaense* extracts. Molecules.

[10] Paudel M.R., Chand M.B., Pant B., Pant B. (2018). Antioxidant and cytotoxic activities of *Dendrobium moniliforme* extracts and the detection of related compounds by GC-MS. BMC Complement. Altern. Med..

[11] Zhang X., Zhang S., Gao B., Qian Z., Liu J., Wu S., Si J. (2019). Identification and quantitative analysis of phenolic glycosides with antioxidant activity in methanolic extract of *Dendrobium catenatum* flowers and selection of quality control herb-markers. Food Res. Int..

[12] Warinhomhoun S., Muangnoi C., Buranasudja V., Mekboonsonglarp W., Rojsitthisak P., Likhitwitayawuid K., Sritularak B. (2021). Antioxidant sctivities and protective effects of dendropachol, a new bisbibenzyl compound from *Dendrobium pachyglossum*, on hydrogen peroxide-induced oxidative stress in HaCaT keratinocytes. Antioxidants.

[13] Zhang X., Zhao R., Zheng S., Chun Z., Hu Y. (2020). *Dendrobium* liquor eliminates free radicals and suppresses cellular proteins expression disorder to protect cells from oxidant damage. J. Food Biochem..

[14] Rao D., Hu Y.D., Zhao R.X., Li H.J., Chun Z., Zheng S.G. (2022). Quantitative identification of antioxidant basis for *Dendrobium nobile* flower by high performance liquid chromatography-tandem mass spectrometry. Int. J. Anal. Chem..

[15] Lei H., Zou S., Lin J., Zhai L., Zhang Y., Fu X., Chen S., Niu H., Liu F., Wu C. (2022). Antioxidant and anti-inflammatory activity of constituents isolated from *Dendrobium nobile* (Lindl.). Front. Chem..

[16] Long Y., Wang W., Zhang Y., Zhang S., Li Z., Deng J., Li J. (2023). *Dendrobium nobile* Lindl polysaccharides attenuate UVB-induced photodamage by regulating oxidative stress, inflammation and MMPs expression in mice model. Photochem. Photobiol..

[17] Zheng S.G., Hu Y.D., Zhao R.X., Yan S., Zhang X.Q., Zhao T.M., Chun Z. (2018). Genome-wide researches and applications on *Dendrobium*. Planta.

[18] Li X.W., Chen H.P., He Y.Y., Chen W.L., Chen J.W., Gao L., Hu H.Y., Wang J. (2018). Effects of rich-polyphenols extract of *Dendrobium loddigesii* on anti-diabetic, anti-inflammatory, anti-oxidant, and gut microbiota modulation in db/db mice. Molecules.

[19] Zheng S., Hu Y., Zhao R., Zhao T., Li H., Rao D., Chun Z. (2020). Quantitative assessment of secondary metabolites and cancer cell inhibiting activity by high performance liquid chromatography fingerprinting in *Dendrobium nobile*. J. Chromatogr. B Anal. Technol. Biomed. Life Sci..

[20] Wang C., Qiu J., Li G., Wang J., Liu D., Chen L., Song X., Cui L., Sun Y. (2022). Application and prospect of quasi-targeted metabolomics in age-related hearing loss. Hear Res..

[21] Gao J.N., Li Y., Liang J., Chai J.H., Kuang H.X., Xia Y.G. (2023). Direct acetylation for full analysis of polysaccharides in edible plants and fungi using reverse phase liquid chromatography-multiple reaction monitoring mass spectrometry. J. Pharm. Biomed. Anal..

[22] Luo Z., Liu L., Nie Q., Huang M., Luo C., Sun Y., Ma Y., Yu J., Du F. (2023). HPLC-based metabolomics of *Dendrobium officinale* revealing its antioxidant ability. Front. Plant. Sci..

[23] Zhao R., Zheng S., Li Y., Zhang X., Rao D., Chun Z., Hu Y. (2022). As a novel anticancer candidate, ether extract of *Dendrobium nobile* overstimulates cellular protein biosynthesis to induce cell stress and autophagy. J. Appl. Biomed..

[24] Gai F., Janiak M.A., Sulewska K., Peiretti P.G., Karamać M. (2023). Phenolic compound profile and antioxidant capacity of flax (*Linum usitatissimum* L.) harvested at different growth stages. Molecules.

[25] de Munter J., Pavlov D., Gorlova A., Sicker M., Proshin A., Kalueff A.V., Svistunov A., Kiselev D., Nedorubov A., Morozov S. (2021). Increased oxidative stress in the prefrontal cortex as a shared feature of depressive- and PTSD-like syndromes: Effects of a standardized herbal antioxidant. Front. Nutr..

[26] Bastin A.R., Nazari-Robati M., Sadeghi H., Doustimotlagh A.H., Sadeghi A. (2022). Trehalose and N-acetyl cysteine alleviate inflammatory vytokine production and oxidative stress in LPS-stimulated human peripheral blood mononuclear cells. Immunol. Investig..

[27] Gao W., Yu T., Li G., Shu W., Jin Y., Zhang M., Yu X. (2021). Antioxidant activity and anti-apoptotic effect of the small molecule procyanidin B1 in early mouse embryonic development produced by somatic cell nuclear transfer. Molecules.

[28] Xiaojin Y., Caiyan L., Lianrong Y., Guoliang X., Zhengqing L., Shizhe C., Xiaodi Y., Hua H. (2022). Study on the antioxidant and anticancer activities of *Sorbus pohuashanensis* (hance) Hedl flavonoids in vitro and its screen of small molecule active components. Nutr. Cancer.

[29] Arzola-Rodríguez S.I., Muñoz-Castellanos L.N., López-Camarillo C., Salas E. (2022). Phenolipids, amphipilic phenolic antioxidants with modified properties and their spectrum of applications in development: A review. Biomolecules.

[30] Shin H.K., Kim T.W., Kim Y.J., Park S.R., Seo C.S., Ha H., Jung J.Y. (2017). Protective effects of *Dendrobium nobile* against cisplatin nephrotoxicity both in-vitro and in-vivo. Iran. J. Pharm. Res..

[31] Tanveer M.A., Rashid H., Nazir L.A., Archoo S., Shahid N.H., Ragni G., Umar S.A., Tasduq S.A. (2023). Trigonelline, a plant derived alkaloid prevents ultraviolet-B-induced oxidative DNA damage in primary human dermal fibroblasts and BALB/c mice via modulation of phosphoinositide 3-kinase-Akt-Nrf2 signalling axis. Exp. Gerontol..

[32] Al-Malki A.L. (2019). Shikimic acid from *Artemisia absinthium* inhibits protein glycation in diabetic rats. Int. J. Biol. Macromol..

[33] de Oliveira D.P., Garcia E.F., de Oliveira M.A., Candido L.C.M., Coelho F.M., Costa V.V., Batista N.V., Queiroz-Junior C.M., Brito L.F., Sousa L.P. (2022). cis-Aconitic Acid, a constituent of *Echinodorus grandiflorus* leaves, inhibits antigen-induced arthritis and gout in mice. Planta Med..

