# Supplementary material for: Revealing of Intracellular Antioxidants in Dendrobium nobile by High Performance Liquid Chromatography-Tandem Mass Spectrometry
Source: Metabolites. 2023 May 28;13(6):702. doi: 10.3390/metabo13060702 (PMC10303210; doi:10.3390/metabo13060702)
Supplement: Supplementary file 1 [file metabolites-13-00702-s001.zip › Figure S2.pdf]

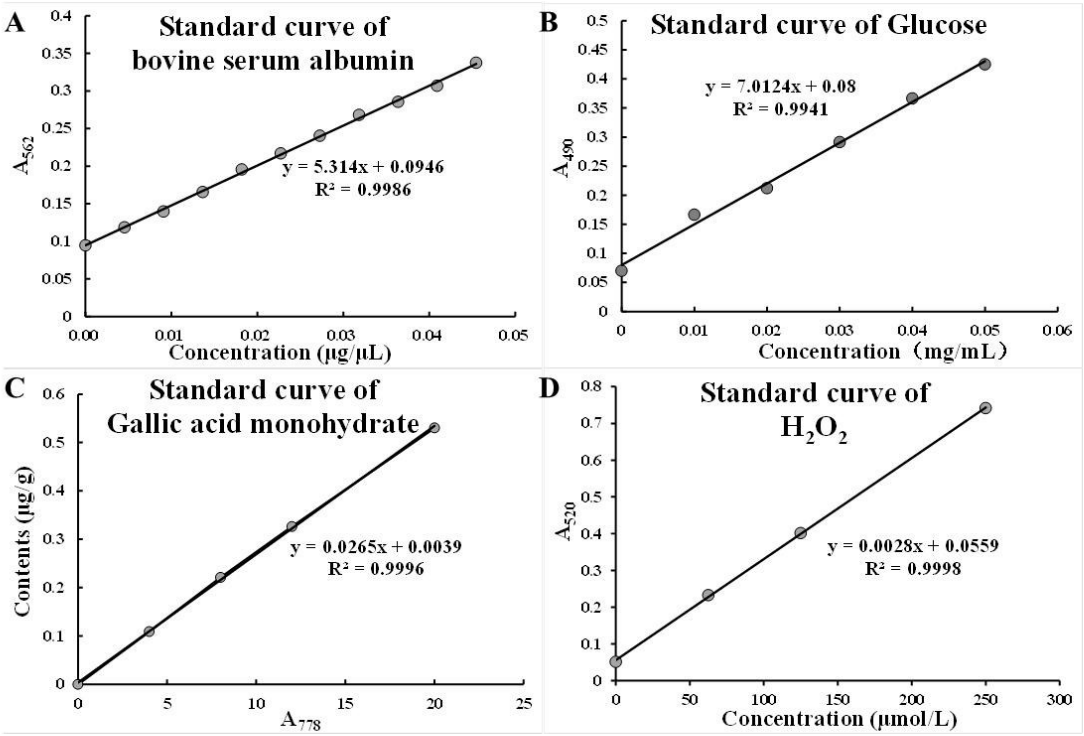

Figure S2 Standard curves used for quantification of total proteins, total soluble saccharides, total phenols, and CAT activities. A Standard curve of bovine serum albumin. B Standard curve of glucose. C Standard curve of gallic acid monohydrate. D Standard curve of H_2O_2.
