# Supplementary material for: Revealing of Intracellular Antioxidants in Dendrobium nobile by High Performance Liquid Chromatography-Tandem Mass Spectrometry
Source: Metabolites. 2023 May 28;13(6):702. doi: 10.3390/metabo13060702 (PMC10303210; doi:10.3390/metabo13060702)
Supplement: Supplementary file 1 [file metabolites-13-00702-s001.zip › Figure S3.pdf]

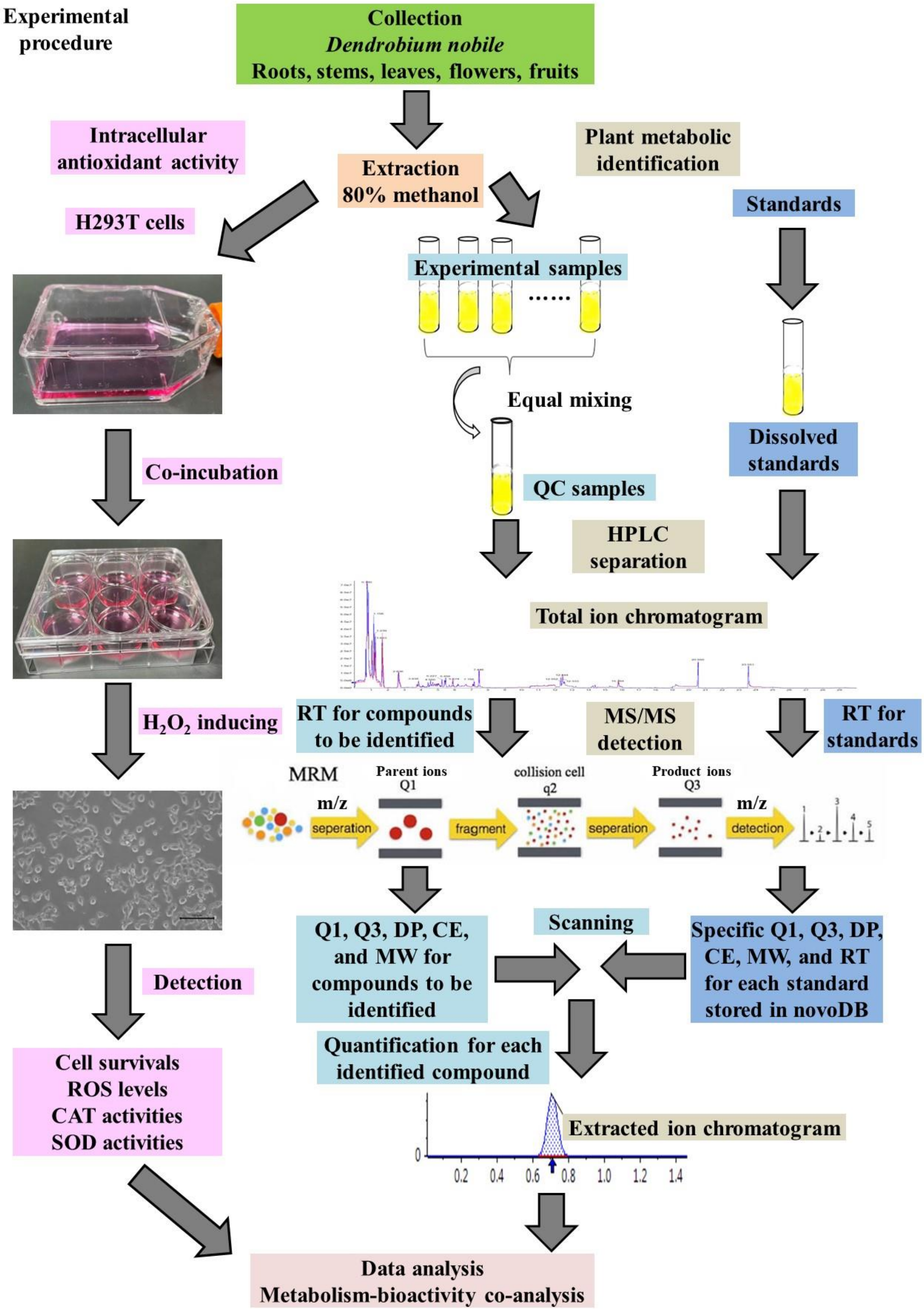

Figure S3 Schematic diagram for the entire experimental procedure in this study. HPLC-MS/MS was mainly supported by Novogene Co., Ltd. (Beijing, China). ROS reactive oxygen species, SOD superoxide dismutase, CAT catalase, Q1 parent ion, Q3 daughter ion, DP declustering potential, CE collision energy, MW molecular weight, RT retention time.
