# Supplementary material for: Revealing of Intracellular Antioxidants in Dendrobium nobile by High Performance Liquid Chromatography-Tandem Mass Spectrometry
Source: Metabolites. 2023 May 28;13(6):702. doi: 10.3390/metabo13060702 (PMC10303210; doi:10.3390/metabo13060702)
Supplement: Supplementary file 1 [file metabolites-13-00702-s001.zip › Figure S4.pdf]

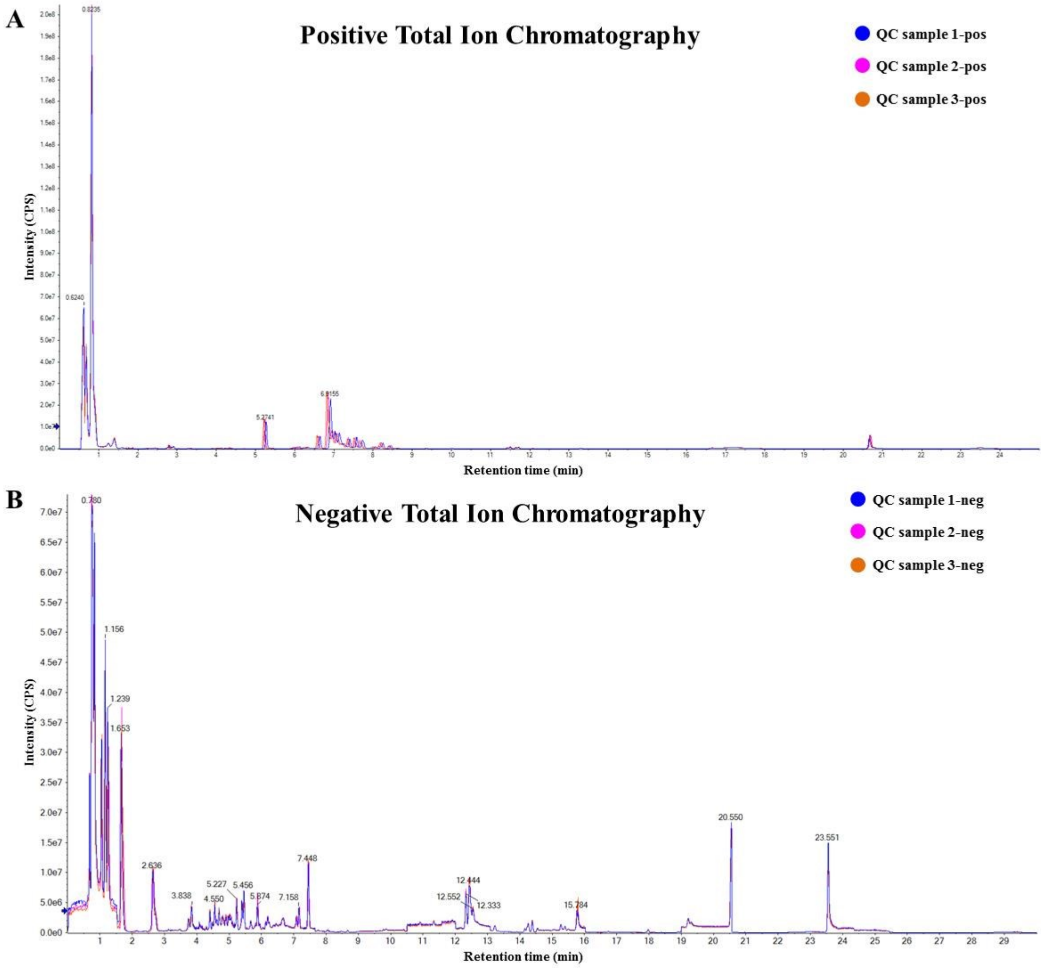

Figure S4 HPLC-MS/MS total ion chromatograms of QC samples. A Positive ion mode. B Negative ion mode. The QC sample is a mixed sample of root, stem, leaf, flower, and fruit in this study, which is used to evaluate the stability and reliability of HPLC-MS/MS.
