# Supplementary material for: Revealing of Intracellular Antioxidants in Dendrobium nobile by High Performance Liquid Chromatography-Tandem Mass Spectrometry
Source: Metabolites. 2023 May 28;13(6):702. doi: 10.3390/metabo13060702 (PMC10303210; doi:10.3390/metabo13060702)
Supplement: Supplementary file 1 [file metabolites-13-00702-s001.zip › Figure S5.pdf]

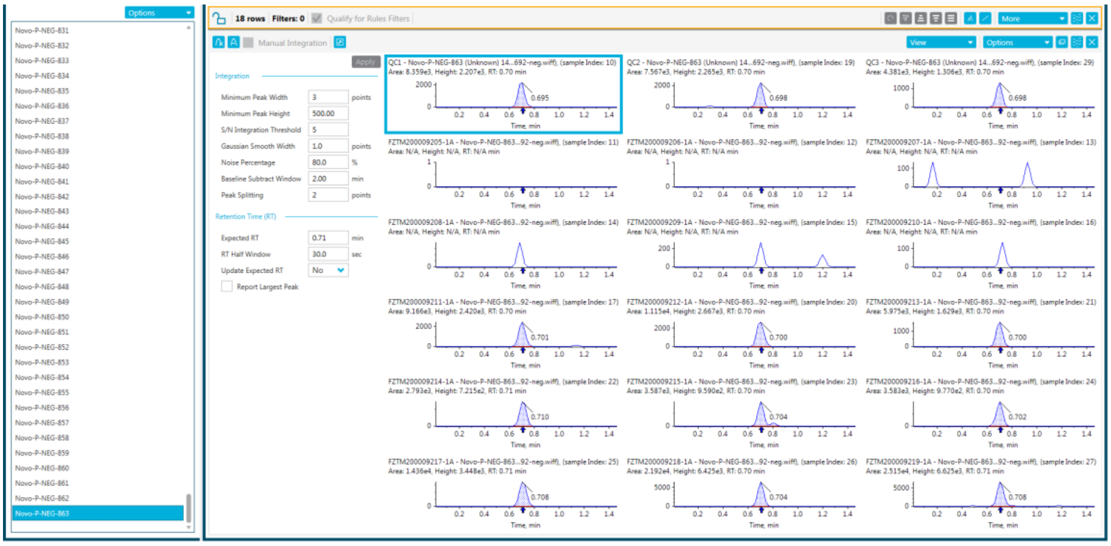

Figure S5 Partial extracted ion chromatograms screened by MRM. Metabolite identification and relative quantification were performed in the software of SCIEX OS Version 1.4. A minimum peak height of 500, a signal/noise ratio of 5, and a Gaussian smooth width of 1 were set for screening of chromatographic peaks. MRM detection was based on an internal database of Beijing Novogene (China).
