# Supplementary material for: Revealing of Intracellular Antioxidants in Dendrobium nobile by High Performance Liquid Chromatography-Tandem Mass Spectrometry
Source: Metabolites. 2023 May 28;13(6):702. doi: 10.3390/metabo13060702 (PMC10303210; doi:10.3390/metabo13060702)
Supplement: Supplementary file 1 [file metabolites-13-00702-s001.zip › Table S1.pdf]

Table S1 Detailed information of 657 metabolites identified by HPLC-MS/MS in *D. nobile*[1]

Compound ID	Name	Formula	MW (Da)	RT (min)	Q1 (m/z)	Class	Peak areas								
							Flower1	Flower2	Flower3	Fruit1	Fruit2	Fruit3	QC1	QC2	QC3
Com_39_neg	(-)-Epigallocatechin gallate	C22H18O11	458.37	5.77	457.08	Organic Acid And Its Derivatives	29,364	18,881	14,263	32,380	25,980	27,680	10,250	11,430	11,700
Com_563_pos	(-)-Maackiain	C16H12O5	284.26	0.60	285.08	Phenols	79,010	10,660	18,460	64,820	50,360	58,730	44,350	26,620	31,700
Com_407_pos	(-)-N-[3',4'-Dihydroxy-(E)-cinnamoyl]-L-glutamic acid	C14H15N1O7	309.27	19.23	310.00	Amino Acid And Its Derivatives	158,100	155,700	181,800	140,600	126,500	167,700	148,800	155,500	145,000
Com_403_pos	(-)-Norepinephrine N-hexoside	C14H21NO8	331.32	0.77	332.13	Others	2,726,000	1,667,000	1,227,000	5,851,000	5,425,000	5,240,000	2,021,000	2,175,000	2,101,000
Com_580_pos	(-)-Pinoresinol	C20H22O6	358.39	8.83	352.11	Phenols	103,905	27,990	37,750	121,200	105,000	110,000	36,010	50,170	46,090
Com_3_neg	(+)-Arabinose	C5H10O5	150.13	0.71	158.25	Carbohydrates	2,793	3,587	3,583	14,360	21,920	25,150	8,359	7,567	4,381
Com_404_pos	(+-)-Baclofen	C10H12ClNO2	213.66	9.53	214.20	Others	423,600	471,000	524,300	400,000	424,300	440,300	358,800	443,200	450,500
Com_356_pos	(+)-pinoresinol 4'-O-β-D-glucopyranoside	C26H32O11NH3	520.53	6.30	538.23	Phenols	43,910	12,910	1,905	47,980	62,990	60,990	20,780	24,040	25,540
Com_401_pos	(+)-Tetrandrine	C38H42N2O6	622.75	5.53	623.31	Alkaloids	61,620	19,400	5,605	248,800	284,100	248,500	46,490	110,500	123,100
Com_575_pos	(2S,3R)-2-{[{9-[(2R,3R,4S,5R)-3,4-Dihydroxy-5-(hydroxymethyl)tetrahydro-2-furanyl]-9H-purin-6-yl}(methyl)carbamoyl]amino}-3-hydroxybutanoic acid (non-preferred name)	C16H22N6O8	426.38	4.06	427.16	Nucleotide And Its Derivates	113,300	76,650	64,330	24,200	22,620	22,820	20,060	29,780	32,350
Com_574_pos	(3,4-Dimethoxyphenyl) Acetic Acid	C10H12O4	196.20	3.76	197.10	Organic Acid And Its Derivatives	61,900	19,380	10,440	261,200	267,800	249,100	85,150	102,800	105,900
Com_572_pos	(S)-(+)-2-(anilinomethyl) pyrrolidine	C11H16N2	176.26	4.06	177.30	Amino Acid And Its Derivatives	176,000	70,100	179,100	21,840	18,410	12,690	20,370	35,600	33,440
Com_678_pos	1,2,3,6,7-Pentamethoxyxanthone	C18H18O7	346.33	29.34	347.11	Others	3,474	3,582	3,592	3,665	4,501	3,913	2,583	5,914	2,353
Com_502_pos	1,3-Dimethyluric Acid	C7H8N4O3	196.16	7.09	197.00	Organic Acid And Its Derivatives	213,173	124,218	78,582	18,180	26,290	29,630	73,090	68,010	53,730
Com_5_neg	1,5-Anhydro-D-glucitol	C6H12O5	164.07	0.73	163.20	Carbohydrates	321,200	128,700	139,900	400,700	434,400	452,200	239,300	233,200	203,100
Com_2_neg	1,6-Anhydro-β-D-Glucopyranose	C6H10O5	162.14	0.73	161.05	Carbohydrates	912,000	593,100	1,727,000	533,100	540,200	541,000	552,500	443,000	573,800
Com_503_pos	10-Formyl-THF	C20H23N7O7	473.44	2.02	474.20	Organic Acid And Its Derivatives	214,000	29,980	64,790	227,100	209,100	215,500	60,530	79,080	87,770
Com_306_neg	12OHJA-Ile-1			8.79	338.00	Organic Acid And Its Derivatives	3,883	767	1,982	23,030	22,020	23,840	28,640	21,700	19,990
Com_504_pos	14,15-Dehydrocrepenynic acid	C18H28O2	276.41	14.12	277.22	Lipids	1,436,000	642,900	166,500	1,617,000	1,604,000	1,759,000	1,638,000	1,825,000	1,833,000
Com_391_pos	16-Hydroxy-hexadecanoic acid	C32H62O5	526.83	10.16	529.20	Lipids	4,985	9,887	7,311	7,961	6,823	7,833	10,180	10,260	6,209
Com_390_pos	18-Nor-4,15-dihydroxyabieta-8,11,13-trien-7-one	C19H26O3	302.41	6.52	303.20	Terpenoids	67,030	36,510	25,110	44,570	40,460	57,010	30,620	18,580	26,960

Com_393_pos	1-Aminocyclopentanecarboxylate	C6H11NO2	129.16	0.84	130.10	Amino Acid And Its Derivatives	572,200	1,117,000	1,292,000	185,200	148,000	150,600	486,000	502,800	495,600
Com_650_pos	1-Aminocyclopropanecarboxylic acid	C4H7NO2	101.10	0.69	102.10	Amino Acid And Its Derivatives	1,656,000	3,521,000	4,536,000	696,500	712,700	703,400	1,730,000	1,618,000	1,582,000
Com_366_pos	1-Deoxynojirimycin	C6H13NO4	163.17	0.81	164.09	Alkaloids	62,240	7,062	12,440	30,340	36,850	34,910	28,980	31,400	27,020
Com_389_pos	1-Linoleoyl-Rac-Glycerol	C21H38O4	354.52	16.67	355.28	Lipids	10,050	15,510	13,010	27,070	30,800	34,360	5,704	5,531	4,930
Com_388_pos	1-Methyladenine	C6H7N5	149.15	0.82	150.00	Nucleotide And Its Derivates	321,600	59,770	56,430	43,000	43,540	44,150	72,030	69,410	54,740
Com_323_pos	1-Methyladenosine	C11H15N5O4	281.27	1.16	282.10	Nucleotide And Its Derivates	327,800	56,020	33,240	103,600	88,080	72,330	90,570	64,830	61,090
Com_324_pos	1-Methylhistidine	C7H11N3O2	169.18	1.10	170.10	Amino Acid And Its Derivatives	378,400	403,600	247,600	464,900	351,400	482,700	217,000	214,300	208,800
Com_307_neg	1-Methyluric acid	C6H6N4O3	182.14	1.09	181.00	Organic Acid And Its Derivatives	43,860	9,470	8,628	28,070	30,050	41,930	26,000	20,510	29,510
Com_308_neg	1-O-b-D-glucopyranosyl sinapate	C17H22O10	386.35	6.55	385.10	Phenols	20,760	2,870	4,321	108,900	99,090	103,600	18,710	19,200	21,950
Com_162_neg	1-O-beta-D-Glucopyranosyl sinapate	C17H22O10	386.10	7.87	385.10	Organic Acid And Its Derivatives	48,570	13,480	6,601	166,500	160,400	148,800	64,300	53,620	58,280
Com_303_neg	2,3,5,4'-Tetrahydroxystilbene-2-O-b-D-glucoside	C20H22O9	406.38	9.30	405.12	Others	1,314,000	26,510	17,620	163,700	153,300	140,800	246,400	284,500	253,800
Com_266_neg	2,3-Dihydroxybenzoic acid	C7H6O4	154.03	0.73	153.20	Organic Acid And Its Derivatives	3,062,000	368,400	271,300	546,000	513,600	675,100	601,300	433,100	468,400
Com_325_pos	2,4-Epibrassinolide	C28H48O6	480.68	11.57	481.35	Others	8,727	9,883	13,260	12,200	19,460	11,650	7,175	5,976	11,130
Com_198_neg	2,5-Dihydroxybenzoic acid	C7H6O4	154.12	0.72	153.00	Organic Acid And Its Derivatives	6,230,000	1,053,000	872,500	927,700	1,058,000	1,027,000	1,270,000	1,335,000	1,183,000
Com_326_pos	2,6-Diaminooimelic Acid	C7H14N2O4	190.20	0.70	191.10	Organic Acid And Its Derivatives	605,200	768,300	497,700	162,900	158,300	160,600	706,200	734,800	668,700
Com_329_pos	2-Amino-1,3,4-tetradecanetriol	C14H31NO3	261.40	7.80	262.30	Amino Acid And Its Derivatives	14,910	15,050	12,920	14,180	2,972	15,720	9,361	12,370	9,567
Com_406_pos	2-Amino-4-hydroxybutyric acid	C4H9NO3	119.12	0.76	120.00	Amino Acid And Its Derivatives	513,000	805,700	1,117,000	218,800	215,400	200,500	420,900	405,800	349,200
Com_625_pos	2'-Deoxyadenosine 5'-monophosphate	C10H14N5O6P	331.22	0.92	332.00	Nucleotide And Its Derivates	156,700	148,300	162,600	305,700	285,800	286,200	92,670	103,800	119,000
Com_627_pos	2'-Deoxycytidine 5'-diphosphate	C9H12N3Na2O7P	351.16	11.75	358.10	Nucleotide And Its Derivates	297,000	2,262,000	2,241,000	294,100	285,600	318,600	388,800	520,200	514,900
Com_626_pos	2'-Deoxycytidine 5'-monophosphate	C9H14N3O7P	307.20	0.84	308.00	Nucleotide And Its Derivates	56,190	23,640	23,990	76,470	58,850	93,880	29,230	39,490	33,580
Com_211_neg	2-Deoxyribose 1-Phosphate	C5H11O7P	214.11	0.57	213.10	Carbohydrates	89,170	24,910	25,730	32,750	39,500	37,940	60,550	32,880	20,660
Com_135_neg	2-Deoxyribose 5'-phosphate	C5H11O7P	214.10	0.65	213.10	Carbohydrates	64,760	134,900	49,280	49,020	33,910	52,430	39,890	45,800	47,800
Com_115_neg	2–furanoic acid	C5H4O3	112.08	0.60	111.02	Organic Acid And Its Derivatives	1,337,000	453,400	319,100	1,070,000	1,330,000	1,359,000	1,120,000	1,069,000	749,000

Com_629_pos	2-Hydroxy-6-Aminopurine	C5H5N5O	151.13	0.95	152.10	Nucleotide And Its Derivates	4,126,000	2,151,000	1,707,000	1,381,000	1,237,000	1,287,000	1,539,000	1,146,000	1,354,000
Com_209_neg	2-Hydroxybutanoic acid	C4H8O3	104.10	0.62	103.00	Organic Acid And Its Derivatives	13,180	13,480	8,795	19,610	23,540	26,500	13,140	10,800	9,175
Com_628_pos	2-Hydroxycinnamate	C9H8O3	164.16	5.46	165.06	Organic Acid And Its Derivatives	832,947	1,026,216	922,975	78,170	75,270	78,100	438,500	487,900	397,400
Com_208_neg	2-Hydroxyisocaproic Acid	C6H12O3	132.16	0.70	131.10	Organic Acid And Its Derivatives	15,480	6,395	13,190	12,840	19,210	9,967	13,600	9,491	8,351
Com_45_neg	2-Isopropylmalic acid	C7H12O5	176.17	0.66	177.05	Organic Acid And Its Derivatives	417,300	404,100	825,200	183,800	191,600	171,600	638,100	511,500	533,900
Com_43_neg	2-Methylglutaric Acid	C6H10O4	146.14	0.64	145.10	Organic Acid And Its Derivatives	507,500	1,836,000	2,370,000	300,200	235,000	332,600	696,300	554,100	571,300
Com_559_pos	2'-O-methyladenosine	C11H15N5O4	281.27	1.17	282.20	Nucleotide And Its Derivates	2,403,000	650,600	908,100	488,000	375,900	405,600	735,100	738,700	660,800
Com_269_neg	2-Oxoadipic acid	C6H8O5	160.04	0.78	159.10	Organic Acid And Its Derivatives	112,500	109,600	79,580	198,500	180,100	206,300	95,910	101,900	92,060
Com_560_pos	2-Phenylglycine	C8H9NO2	151.16	1.16	152.00	Amino Acid And Its Derivatives	4,586,000	2,294,000	2,223,000	1,562,000	1,400,000	1,509,000	1,827,000	1,728,000	1,786,000
Com_561_pos	2-Pyridinecarboxylic acid	C6H5NO2	123.11	0.87	124.00	Organic Acid And Its Derivatives	1,964,000	1,967,000	1,433,000	1,104,000	966,600	1,030,000	1,377,000	1,307,000	1,192,000
Com_200_neg	3-(2-Naphthyl)-D-alanine	C13H13NO2	215.10	5.90	214.10	Amino Acid And Its Derivatives	23,380	4,786	3,989	30,580	35,380	20,390	13,820	9,574	4,066
Com_554_pos	3,4-Dihydroxybenzaldehyde	C7H6O3	138.12	3.31	139.04	Phenols	1,062,000	134,400	141,900	89,944	92,160	83,478	166,600	222,000	154,400
Com_274_neg	3,7-Di-O-methylquercetin	C17H14O7	330.07	11.80	329.10	Flavonoids	3,828,000	420,700	501,300	235,100	231,600	227,000	1,326,000	1,352,000	1,061,000
Com_439_pos	3',4',5'-Tricetin 3-O-hexoside	C24H26O12	506.46	6.51	507.15	Flavonoids	126,100	20,430	13,880	268,200	274,200	264,700	60,300	70,670	83,210
Com_292_neg	3-Aminosalicylic Acid	C7H7NO3	153.14	3.49	152.00	Organic Acid And Its Derivatives	111,629	106,889	128,487	159,913	138,264	127,873	117,300	87,620	58,210
Com_438_pos	3-Chloro-L-Tyrosine	C9H10ClNO3	215.63	4.35	216.00	Amino Acid And Its Derivatives	260,400	72,470	30,730	66,770	37,660	48,540	111,000	123,700	81,850
Com_291_neg	3-Dehydroshikimic acid	C7H8O5	172.14	0.57	171.03	Organic Acid And Its Derivatives	3,183,000	3,034,000	2,988,000	2,204,000	2,246,000	2,629,000	2,151,000	2,031,000	1,924,000
Com_293_neg	3-Hydroxy-4-methyl-3-carboxypentanoate			0.70	175.00	Others	275,800	158,500	272,600	888,800	548,400	458,000	384,900	439,800	493,600
Com_429_pos	3-Hydroxy-9,10-Dimethoxypterocarpan	C17H16O5	300.31	0.63	301.11	Others	55,780	42,530	23,120	123,700	97,840	96,350	45,560	54,360	59,530
Com_290_neg	3-Hydroxybutyrate	C4H8O3	104.10	0.56	103.10	Organic Acid And Its Derivatives	1,246,000	102,900	124,700	78,570	83,330	109,900	285,200	172,100	217,700
Com_242_neg	3-Hydroxy-glabrol	C25H28O5	40.50	0.61	40.70	Phenols	3,733	10,010	5,538	10,610	11,700	7,895	7,547	6,336	2,018
Com_637_pos	3-Hydroxyhippuric Acid	C9H9NO4	195.17	0.94	196.10	Organic Acid And Its Derivatives	68,600	10,650	3,258	248,000	216,400	188,500	116,300	114,800	95,950
Com_289_neg	3-Hydroxymorindone	C15H10O6	286.24	12.98	285.04	Phenols	14,879	11,140	7,230	15,200	13,340	13,050	1,504	1,537	1,810

Com_244_neg	3-Indoleacrylic acid	C11H9NO2	187.20	4.54	186.06	Amino Acid And Its Derivatives	50,810	303,000	325,600	97,150	72,160	65,130	98,730	102,400	105,900
Com_147_neg	3-O-Feruloylquinic acid	C17H20O9	368.34	4.71	367.00	Organic Acid And Its Derivatives	55,990	9,687	10,680	130,600	104,500	109,000	35,270	36,820	27,080
Com_188_neg	3-Phenylpropanoic acid	C9H10O2	150.17	0.63	149.06	Organic Acid And Its Derivatives	37,230	18,030	23,480	34,410	30,000	40,910	19,830	21,740	19,600
Com_649_pos	4,6-Dihydroxyquinoline O-hexoside			12.61	324.20	Amino Acid And Its Derivatives	12,980	7,887	9,281	15,790	26,340	28,100	9,757	6,172	7,242
Com_697_pos	4-Acetamidobutyric Acid	C6H11NO3	145.16	0.78	146.10	Organic Acid And Its Derivatives	1,308,000	669,600	917,700	1,287,000	1,089,000	1,095,000	1,814,000	1,638,000	1,601,000
Com_698_pos	4beta-Hydroxy-11-O-(2'-pyrolylcarboxy)epilupinine	C15H22N2O3	278.35	5.66	279.10	Alkaloids	34,950	30,600	36,750	12,580	10,010	12,600	12,970	14,010	10,410
Com_696_pos	4-Dimethylaminobenzaldehyde	C9H11NO	149.19	0.79	150.09	Others	58,770	16,380	11,480	35,750	43,950	31,880	18,670	7,031	10,220
Com_695_pos	4-Guanidinobutyric acid	C5H11N3O2	145.16	0.78	146.10	Organic Acid And Its Derivatives	4,280,000	1,535,000	1,508,000	6,353,000	5,431,000	5,751,000	6,217,000	6,001,000	5,501,000
Com_394_pos	4-Hydroxybenzoate	C7H6O3	138.12	2.98	139.00	Phenols	1,326,000	130,400	179,200	42,000	32,790	39,990	250,300	221,500	257,100
Com_617_pos	4'-O-β-Glucopyranosyl-5-O-methylvisamminol	C22H28O10	452.45	6.52	453.18	Flavonoids	148,600	52,950	40,690	98,820	117,700	148,600	84,150	117,900	85,850
Com_241_neg	4-p-Coumaroylquinic acid	C16H18O8	338.31	3.97	337.10	Phenols	28,340	3,518	2,764	9,891	9,449	9,163	5,991	7,310	10,300
Com_613_pos	4-Pyridoxate	C8H9NO4	183.16	1.06	184.10	Organic Acid And Its Derivatives	144,100	40,350	20,790	39,230	37,080	18,620	83,730	93,530	83,160
Com_494_pos	5,6-Dihydro-5-methyluracil	C5H8N2O2	128.13	0.60	129.10	Nucleotide And Its Derivates	299,200	580,200	563,200	361,800	315,500	335,100	288,000	276,800	279,900
Com_562_pos	5-Aminosalicylic Acid	C7H7NO3	153.14	1.46	154.00	Organic Acid And Its Derivatives	630,500	6,001,000	4,812,000	217,800	191,600	187,900	718,200	752,700	805,000
Com_483_pos	5-Ethyl-2'-deoxyuridine	C11H16N2O5	256.26	0.64	257.10	Nucleotide And Its Derivates	55,180	35,160	24,750	85,470	80,910	87,010	97,880	61,550	83,570
Com_136_neg	5-hydroxyhexanoic acid	C6H12O3	132.08	0.74	131.08	Organic Acid And Its Derivatives	74,370	35,090	39,170	91,050	98,680	75,800	57,080	37,900	38,350
Com_701_pos	5-Hydroxymethylfurfural	C6H6O3	126.11	0.78	127.04	Others	227,800	131,700	126,800	263,600	224,300	245,000	175,500	159,800	141,000
Com_421_pos	5-Methoxyindoleacetic acid	C11H11NO3	205.21	2.55	206.00	Amino Acid And Its Derivatives	408,600	1,519,000	1,180,000	379,600	308,800	325,300	912,800	952,100	865,100
Com_488_pos	5-Methylcytidine	C10H15N3O5	257.24	0.71	258.20	Nucleotide And Its Derivates	380,200	174,600	137,800	421,300	313,700	270,100	223,500	198,100	221,200
Com_557_pos	5-Methylcytosine	C5H7N3O	125.13	1.38	126.10	Nucleotide And Its Derivates	76,320	20,990	30,510	76,107	74,682	70,143	27,390	22,030	12,740
Com_556_pos	5-Methyltryptamine	C11H14N2	174.24	8.13	175.10	Amino Acid And Its Derivatives	23,320	22,050	4,663	65,740	31,730	43,740	30,320	45,510	46,160
Com_236_neg	5-O-p-Coumaroylquinic acid	C16H18O8	338.00	4.20	337.00	Organic Acid And Its Derivatives	94,820	25,440	27,990	19,420	17,820	18,680	20,670	22,470	22,200
Com_151_neg	5-Oxoproline	C5H7NO3	129.04	0.75	128.00	Amino Acid And Its Derivatives	343,700	93,890	149,000	22,810	20,840	17,600	97,590	98,790	75,480

Com_175_neg	5-Phosphoribosyl 1-pyrophosphate	C5H13O14P3	390.07	0.62	389.00	Carbohydrates	43,120	23,570	31,310	39,350	28,430	27,780	28,320	31,600	21,830
Com_699_pos	6,7-Dimethoxy-4-Methylcoumarin	C12H12O4	220.22	6.51	221.10	Phenylpropanoids	83,950	28,877	27,063	73,500	70,730	51,050	36,910	32,390	38,160
Com_700_pos	6-Aminohexanoic acid	C6H13NO2	131.17	1.16	132.00	Amino Acid And Its Derivatives	185,000	381,700	383,500	70,250	62,250	69,860	123,900	98,010	109,400
Com_199_neg	6-Deoxycatalpol +HCOOH	C15H22O9.HCOOH	392.35	17.58	391.13	Others	638	6,938	10,000	5,026	4,706	1,680	842	1,279	1,351
Com_364_pos	6-Formyl-isoophiopogonanone A	C19H16O7	356.33	0.62	357.10	Flavonoids	31,640	10,760	5,799	79,310	77,250	87,230	48,560	54,660	48,460
Com_693_pos	6-Gingerol	C17H26O4	294.39	10.52	307.17	Phenols	204,000	285,200	196,900	1,286,000	1,227,000	1,234,000	1,017,000	462,400	516,300
Com_376_pos	6-Hydroxynicotinic acid	C6H5NO3	139.11	1.00	140.00	Organic Acid And Its Derivatives	400,400	106,100	69,570	360,300	300,300	290,300	216,100	193,100	238,800
Com_181_neg	6-Phosphogluconic acid	C6H13O10P	276.14	0.67	275.00	Carbohydrates	65,180	34,580	16,990	47,480	56,020	45,850	26,720	27,920	42,220
Com_374_pos	6-Sialyllactose	C23H39NO19	633.55	0.74	634.22	Carbohydrates	44,240	36,520	20,300	14,190	14,900	9,145	13,510	18,630	15,930
Com_631_pos	7-Methylguanine	C6H7N5O	165.15	5.60	166.00	Nucleotide And Its Derivates	154,200	17,440	24,730	35,000	36,390	29,510	51,150	62,750	54,060
Com_593_pos	7-O-Ethylmorroniside	C19H30O11	434.44	0.62	457.17	Terpenoids	155,900	21,720	21,630	92,280	85,060	86,070	50,690	64,140	36,600
Com_373_pos	7-O-Methylaloeresin A	C29H30O11	554.54	7.22	555.19	Others	128,000	120,600	60,180	46,700	36,780	36,030	29,100	40,970	32,150
Com_372_pos	8-Chlro-1-tetrahydronorharmanone	C11H9ClN2O	220.66	1.07	221.00	Others	63,410	122,000	84,420	57,420	50,850	47,510	50,860	54,580	47,660
Com_233_neg	8-Gingerol	C43H32O20	868.70	0.84	871.21	Phenols	98,290	352,300	383,600	35,680	45,220	43,670	150,400	132,900	146,400
Com_451_pos	8-hydroxy-6,7-dimethoxycoumarin	C11H10O5	222.20	11.26	223.06	Phenylpropanoids	1,737,000	1,788,000	1,472,000	1,665,000	1,250,000	1,306,000	1,921,000	2,439,000	2,751,000
Com_378_pos	8-Hydroxyguanosine	C10H13N5O6	299.24	0.97	300.10	Nucleotide And Its Derivates	148,900	45,990	20,090	38,260	17,410	35,770	41,530	42,710	76,330
Com_87_neg	8-O-Methylreyusin	C17H14O5	298.29	11.38	297.08	Others	10,000	8,271	4,075	107,400	88,940	87,720	33,010	29,810	28,730
Com_379_pos	8-Oxo-dGMP	C10H13N5O5	283.24	17.78	284.00	Nucleotide And Its Derivates	4,637	9,178	14,320	2,482	2,965	1,878	2,270	3,710	5,727
Com_183_neg	9,10-EODE	C18H32O3	296.00	0.70	295.00	Lipids	536,200	368,600	484,400	422,000	751,500	453,100	314,100	478,600	555,600
Com_86_neg	9-Hotre	C18H30O3	294.43	14.33	293.00	Lipids	20,460	3,659	1,918	35,890	29,580	36,920	51,140	52,240	45,150
Com_133_neg	9-HpOTrE	C18H30O4	310.10	15.80	309.10	Lipids	55,880	15,880	25,600	4,410	4,380	4,838	12,410	11,650	14,660
Com_489_pos	9-Hydroxy-(10E,12Z,15Z)-octadecatrienoic acid	C18H30O3	294.43	15.21	295.20	Lipids	3,111,000	603,700	806,200	1,008,000	921,100	1,060,000	2,194,000	2,402,000	2,306,000
Com_491_pos	Abscisic acid	C15H20O4	264.32	7.14	265.14	Organic Acid And Its Derivatives	80,870	59,210	45,460	58,090	96,453	95,088	90,130	109,400	61,420
Com_81_neg	Acacetin	C16H12O5	284.26	12.01	283.00	Flavonoids	15,305	9,964	6,526	9,122	7,742	8,513	2,803	3,020	2,135
Com_490_pos	Acetaminophen	C8H9NO2	151.16	1.17	152.10	Phenols	4,389,000	2,527,000	2,003,000	1,283,000	1,137,000	1,308,000	1,648,000	1,449,000	1,451,000
Com_492_pos	Acetylcholine	C7H16ClNO2	181.66	0.82	164.10	Others	4,970,000	1,976,000	1,781,000	7,219,000	6,828,000	7,008,000	7,617,000	6,669,000	7,184,000
Com_605_pos	Adenine	C5H5N5	135.13	0.75	136.06	Nucleotide And Its Derivates	3,917,000	2,062,000	1,364,000	2,010,000	1,937,000	2,047,000	1,785,000	1,765,000	1,493,000
Com_604_pos	Adenosine	C10H13N5O4	267.24	0.97	268.10	Nucleotide And Its Derivates	#########	#########	#########	#########	#########	#########	#########	#########	#########
Com_180_neg	Adenosine 5'-monophosphate	C10H14N5O7P	347.22	0.80	346.00	Nucleotide And Its Derivates	150,400	465,800	409,800	527,200	593,300	355,600	468,000	462,200	378,200

Com_608_pos	Adenosine O-ribose			0.94	400.20	Nucleotide And Its Derivates	103,900	74,260	67,310	119,100	101,400	104,200	84,940	82,590	79,790
Com_606_pos	Adenosine-3'-monophosphate	C10H14N5O7P	347.22	0.70	348.10	Nucleotide And Its Derivates	899,700	3,278,000	2,369,000	2,794,000	2,696,000	999,600	1,678,000	1,799,000	1,873,000
Com_114_neg	Adipic acid	C6H10O4	146.06	0.70	145.06	Organic Acid And Its Derivatives	539,500	2,331,000	2,940,000	252,100	313,200	355,500	769,100	731,900	737,100
Com_256_neg	Afzelechin	C15H14O5	274.08	0.70	273.00	Flavonoids	357,500	287,100	281,100	476,300	434,300	432,600	288,800	328,300	322,400
Com_332_pos	Agmatine Sulfate	C5H14N4	130.19	0.63	131.13	Phenols	5,984	4,718	5,818	8,916	9,463	10,350	6,275	12,080	7,479
Com_333_pos	Albiflorin	C23H28O11	480.46	10.31	481.17	Terpenoids	68,873	28,988	38,040	93,030	97,010	100,400	20,560	22,170	22,520
Com_121_neg	Allantoin	C4H6N4O3	158.12	0.67	157.00	Others	43,320	39,710	55,010	18,400	22,010	17,670	74,700	65,500	68,320
Com_331_pos	Allopurinol	C5H4N4O	136.11	0.70	137.00	Nucleotide And Its Derivates	457,900	200,100	149,700	235,500	214,100	179,500	234,400	186,600	188,000
Com_316_neg	Allysine	C6H11NO3	145.07	0.70	144.10	Amino Acid And Its Derivatives	89,090	74,520	95,390	55,430	58,510	39,670	95,380	89,430	89,450
Com_119_neg	alpha-Ketoglutaric acid	C5H6O5	146.10	0.67	145.00	Organic Acid And Its Derivatives	88,960	236,900	223,300	22,050	26,810	26,410	76,060	61,280	36,840
Com_476_pos	alpha-Linolenic acid	C18H30O2	278.43	16.75	279.23	Lipids	168,500	25,610	22,210	28,150	24,150	33,860	50,600	56,640	57,790
Com_327_pos	alpha-Terthienyl	C12H8S3	248.39	6.84	248.99	Others	12,080	3,556	2,227	18,290	19,500	20,740	20,140	17,430	24,140
Com_116_neg	Amentoflavone	C30H18O10	538.46	10.70	537.10	Flavonoids	32,831	23,924	19,587	27,290	22,110	26,140	14,830	12,120	14,420
Com_155_neg	Aminomalonic acid	C3H5NO4	119.02	0.73	118.00	Organic Acid And Its Derivatives	1,168,000	746,700	1,041,000	423,000	482,000	458,200	683,600	665,000	656,100
Com_117_neg	Androsin	C15H20O8	328.32	1.59	327.11	Phenols	343,000	82,700	143,100	98,138	103,639	96,896	150,900	103,400	109,400
Com_449_pos	ANGELOL-K	C20H24O7	376.41	12.24	377.16	Others	649,500	2,196,000	1,274,000	1,231,000	783,600	769,400	700,500	705,100	692,400
Com_222_neg	Apigenin	C15H10O5	270.24	9.82	269.00	Flavonoids	20,284	20,023	16,354	17,723	16,975	15,086	14,250	13,020	10,460
Com_221_neg	Apigenin 4-O-rhamnoside	C21H20O9	416.38	9.91	415.10	Flavonoids	11,340	4,430	22,238	17,210	10,720	16,620	53,160	61,640	79,930
Com_224_neg	Apigenin 5-O-glucoside	C21H20O10	432.38	6.93	431.10	Flavonoids	71,610	13,540	43,510	301,800	282,600	231,500	154,100	135,900	154,500
Com_18_neg	Apigenin-6,8-di-C-glycoside	C27H30O15	594.52	4.83	593.15	Flavonoids	#########	1,783,000	1,349,000	3,869,000	3,448,000	3,462,000	#########	#########	#########
Com_223_neg	Apigenin-7-glucoside	C21H20O10	432.38	7.15	431.10	Flavonoids	611,000	56,440	169,100	41,890	35,270	35,730	175,300	159,600	193,900
Com_447_pos	Apo-13-zeaxanthinone	C18H26O2	274.40	14.54	275.20	Others	50,620	20,350	9,563	9,648	16,790	15,950	25,390	26,270	18,910
Com_567_pos	Arabidopyl ketoadipic acid	C15H14O9	338.27	18.27	339.07	Organic Acid And Its Derivatives	37,600	28,520	30,920	51,950	60,590	64,450	32,980	39,050	35,380
Com_568_pos	Arabidopyl shikimic acid	C16H16O9	352.29	14.54	353.09	Organic Acid And Its Derivatives	27,870	17,730	20,400	37,170	28,820	37,680	16,770	20,110	18,540
Com_566_pos	Arctigenin	C21H24O6	372.41	7.79	373.17	Phenols	11,700	2,409	11,942	14,490	16,770	19,960	10,530	15,240	21,150
Com_565_pos	Artemisinin	C15H22O5	282.33	19.22	283.15	Terpenoids	850,300	894,600	933,800	854,800	852,900	884,000	980,000	854,800	988,100
Com_313_neg	Astragalin	C21H20O11	448.38	7.54	447.10	Flavonoids	#########	#########	7,047,000	659,600	610,100	646,500	7,104,000	6,739,000	6,636,000
Com_179_neg	ATP	C10H16N5O13P3	507.18	0.70	506.00	Nucleotide And Its Derivates	38,430	14,480	18,640	36,870	28,640	23,970	18,570	17,440	26,770
Com_704_pos	Aucubin	C15H22O9	346.33	1.07	369.11	Lipids	32,530	11,780	15,430	35,710	27,300	25,570	42,630	32,750	45,550
Com_703_pos	Azelaic Acid	C9H16O4	188.22	0.98	189.10	Organic Acid And Its Derivatives	64,120	29,560	11,010	79,770	60,640	62,800	50,310	32,050	21,000
Com_702_pos	Benzamidine	C7H8N2	120.15	1.65	121.10	Phenols	4,037,000	#########	#########	1,128,000	957,300	972,500	2,753,000	2,783,000	2,537,000
Com_484_pos	Berberrubine	C19H16NO4.Cl	357.79	5.64	372.11	Alkaloids	57,940	6,085	1,075	24,330	12,390	12,210	15,890	11,530	9,048

Com_718_pos	Bergapten	C12H8O4	216.19	8.38	217.05	Phenylpropanoids	17,230	24,550	2,458	67,310	75,850	72,660	46,790	43,910	39,830
Com_507_pos	beta-Carotene	C40H56	536.87	23.59	537.45	Terpenoids	#########	#########	#########	#########	#########	#########	#########	#########	#########
Com_501_pos	Betaine	C5H11NO2	117.15	0.82	118.10	Alkaloids	#########	#########	6,393,000	#########	#########	#########	#########	#########	#########
Com_387_pos	beta-Nicotyrine	C10H10N2	158.20	2.84	159.09	Alkaloids	609,200	2,881,000	2,360,000	648,500	578,900	592,700	625,200	662,500	640,000
Com_72_neg	Betulinic acid	C30H48O3	456.70	18.17	455.35	Terpenoids	88,866	70,225	73,231	82,680	24,340	26,710	33,320	79,890	46,670
Com_495_pos	Biliverdin	C33H34N4O6	582.65	11.00	583.25	Others	5,449	44,120	18,770	16,080	20,600	25,170	20,170	12,040	13,670
Com_37_neg	Boc-β-cyano-L-alanine	C9H14N2O4	214.22	5.78	227.18	Amino Acid And Its Derivatives	18,838	12,233	605	14,470	16,450	15,930	8,381	7,054	6,028
Com_630_pos	Butylphthalide	C12H14O2	190.24	15.81	181.21	Others	6,204	2,428	6,912	7,082	2,790	4,084	3,615	2,461	3,971
Com_367_pos	Caffeic Acid	C9H8O4	180.16	3.08	181.05	Organic Acid And Its Derivatives	#########	#########	#########	89,830	110,400	59,810	#########	#########	#########
Com_235_neg	Caffeic acid O-glucoside	C15H18O9	342.00	1.40	341.00	Organic Acid And Its Derivatives	7,279,000	#########	#########	1,974,000	1,365,000	1,636,000	2,617,000	2,931,000	2,626,000
Com_543_pos	Calycosin-7-O-glucoside	C22H22O10	446.40	5.32	447.13	Flavonoids	507,400	19,550	15,530	59,920	32,080	50,550	58,480	74,370	70,140
Com_30_neg	Cellotriose	C18H32O16	504.44	0.91	503.20	Carbohydrates	1,485,000	1,096,000	1,979,000	945,500	823,100	694,500	844,300	799,000	854,200
Com_601_pos	C-hexosyl-apigenin O-hexosyl-O-hexoside	C33H40O20	756.66	3.73	757.22	Flavonoids	68,880	25,863	23,864	30,750	22,330	25,580	26,850	28,300	28,350
Com_602_pos	C-hexosyl-apigenin O-p-coumaroylhexoside	C36H36O17	740.66	5.88	741.20	Flavonoids	226,900	55,760	17,610	28,080	31,050	29,500	76,680	85,090	105,300
Com_527_pos	Chinese bittersweet alkaloid II	C18H33N3O3	339.47	6.75	340.10	Alkaloids	77,580	30,220	36,980	171,326	160,282	163,033	124,600	140,700	186,400
Com_66_neg	Chlorogenic acid methyl ester	C17H20O9	368.11	5.20	367.00	Organic Acid And Its Derivatives	87,670	11,540	10,920	129,000	127,900	140,500	41,130	43,660	40,730
Com_529_pos	Cholesterol	C27H46O	386.65	13.04	387.10	Lipids	1,315,000	1,198,000	1,135,000	1,186,000	1,113,000	1,120,000	931,600	1,054,000	1,075,000
Com_528_pos	Choline	C5H14NO+	104.17	0.70	105.10	Others	1,959,000	1,769,000	2,048,000	1,237,000	1,259,000	1,331,000	1,241,000	1,128,000	1,126,000
Com_524_pos	Chrysoeriol	C16H12O6	300.26	8.98	301.10	Flavonoids	107,800	27,490	63,720	183,600	171,000	189,000	56,370	65,150	73,200
Com_74_neg	Chrysoeriol 8-C-pentosyl-O-rutinoside		740.20	8.07	739.20	Flavonoids	132,700	1,507	3,305	8,490	9,188	6,021	15,760	17,730	14,350
Com_416_pos	Chrysoeriol C-hexoside	C22H22O11	462.40	5.69	463.12	Flavonoids	2,751,000	234,100	583,800	639,700	560,300	644,100	562,200	586,000	620,400
Com_73_neg	Chrysoeriol O-acetylhexoside		504.10	0.90	503.10	Flavonoids	245,400	279,100	366,900	160,700	180,300	184,600	221,100	189,500	254,100
Com_564_pos	Cinnamoyl tyramine			4.40	268.00	Amino Acid And Its Derivatives	8,511	7,438	8,196	18,040	22,690	23,090	37,450	23,300	31,370
Com_186_neg	Citraconic Acid	C5H6O4	130.10	0.57	129.00	Organic Acid And Its Derivatives	539,800	310,200	231,900	471,000	413,900	546,800	491,600	481,600	403,300
Com_132_neg	Citramalate	C5H8O5	148.00	0.60	147.00	Organic Acid And Its Derivatives	28,840	11,050	7,108	20,600	11,030	28,080	13,250	8,610	8,318
Com_8_neg	Citric acid	C6H8O7	192.03	0.60	191.00	Organic Acid And Its Derivatives	#########	#########	#########	#########	#########	#########	#########	#########	#########
Com_348_pos	COLUMBIANETIN ACETATE	C16H16O5	288.30	12.56	289.11	Phenylpropanoids	38,066	26,781	28,479	82,620	85,220	66,760	21,350	24,700	17,950
Com_276_neg	Columbin	C20H22O6	358.39	10.83	313.22	Others	8,408	1,013	4,926	11,050	9,957	11,390	1,935	2,540	1,828
Com_347_pos	Coniferyl aldehyde	C10H10O3	178.19	14.95	179.10	Phenols	142,100	23,060	44,450	31,860	31,740	48,580	98,650	115,100	105,000
Com_646_pos	Cordycepin	C10H13N5O3	251.25	1.27	216.07	Alkaloids	3,988,000	1,969,000	1,696,000	1,178,000	867,200	894,200	1,545,000	1,504,000	1,376,000
Com_350_pos	Corydaline	C22H27NO4	369.45	7.28	370.20	Alkaloids	31,582	10,480	3,835	31,680	31,450	29,290	5,949	14,490	10,450

Com_653_pos	Coumarin	C9H6O2	146.14	6.51	147.10	Phenylpropanoids	316,600	7,394	53,222	26,900	26,980	21,580	284,900	297,400	281,300
Com_656_pos	C-pentosyl-C-hexosyl-apigenin	C26H28O14	564.49	4.70	565.16	Flavonoids	471,200	74,280	25,110	5,015,000	4,640,000	4,868,000	933,400	1,036,000	1,012,000
Com_278_neg	Creatine	C4H9N3O2	131.13	0.70	130.10	Amino Acid And Its Derivatives	396,800	543,400	495,400	288,500	164,300	309,800	200,000	221,500	206,000
Com_279_neg	Crocetin	C20H24O4	328.40	10.65	327.16	Organic Acid And Its Derivatives	135,300	16,200	4,216	42,250	36,990	36,910	84,340	88,010	90,810
Com_210_neg	Crotonoside	C10H13N5O5	283.24	2.73	282.08	Alkaloids	#########	9,265,000	8,470,000	7,390,000	7,688,000	7,137,000	9,587,000	8,623,000	7,984,000
Com_280_neg	Curculigoside	C22H26O11	466.44	18.35	465.14	Phenols	9,641	8,168	4,417	19,540	13,100	7,269	1,925	2,418	1,576
Com_184_neg	Cyanidin 3-O-glucoside	C21H21O11	448.30	8.33	447.30	Flavonoids	2,431,000	1,084,000	476,600	88,670	82,630	81,530	307,600	296,400	284,900
Com_76_neg	Cyanidin O-acetylhexoside		490.10	8.17	489.10	Flavonoids	1,579,000	1,991,000	1,982,000	26,590	28,730	25,920	361,900	334,300	303,400
Com_75_neg	Cyanidin O-diacetyl-hexoside-O-glyceric acid		620.10	8.46	619.10	Flavonoids	68,530	155,300	85,670	266,100	234,000	253,500	51,970	45,350	63,720
Com_711_pos	Cyanidin O-rutinoside	C27H31O15	595.52	5.25	595.17	Flavonoids	3,703,000	6,834,000	2,794,000	708,100	617,900	714,600	647,600	885,600	880,800
Com_673_pos	Cytidine	C9H13N3O5	243.22	0.74	244.10	Nucleotide And Its Derivates	6,986,000	1,914,000	1,645,000	2,515,000	2,573,000	2,786,000	3,010,000	2,983,000	2,978,000
Com_671_pos	Cytosine	C4H5N3O	111.10	0.72	112.00	Nucleotide And Its Derivates	3,194,000	1,096,000	774,200	1,288,000	1,188,000	1,187,000	1,528,000	1,428,000	1,402,000
Com_228_neg	D-(-)-Salicin	C13H18O7	286.28	4.71	285.10	Organic Acid And Its Derivatives	26,740	26,000	7,132	16,890	16,510	20,100	6,563	6,613	6,839
Com_620_pos	D-(+)-Octopine	C9H18N4O4	246.26	0.70	247.14	Amino Acid And Its Derivatives	112,500	59,880	39,340	77,130	74,870	62,940	87,380	80,560	94,300
Com_622_pos	D-2-Aminobutyric acid	C4H9NO2	103.12	0.71	104.10	Organic Acid And Its Derivatives	6,150,000	5,840,000	6,488,000	4,102,000	3,771,000	4,041,000	3,903,000	3,636,000	3,466,000
Com_41_neg	D-2-Hydroxypentanedioic acid	C5H6Na2O5	192.08	0.51	189.02	Amino Acid And Its Derivatives	1,052,000	655,000	503,200	1,136,000	1,172,000	1,171,000	832,600	583,300	577,400
Com_40_neg	D-3-Phosphoglyceric acid	C3H5H2O7P	230.02	0.50	248.99	Lipids	55,410	59,770	61,460	101,100	87,560	86,470	137,900	158,200	117,200
Com_618_pos	Daidzein	C15H10O4	254.24	3.85	257.12	Flavonoids	17,150	13,840	18,970	91,790	95,030	91,430	171,300	158,400	111,900
Com_619_pos	D-Arabitol	C5H12O5	152.15	0.62	175.06	Carbohydrates	#########	#########	#########	#########	#########	#########	#########	#########	#########
Com_24_neg	dATP	C10H14N5Na2O12P3	535.15	0.62	549.00	Nucleotide And Its Derivates	67,910	56,970	32,420	576,600	601,200	538,800	185,900	177,100	183,600
Com_616_pos	D-beta-Homophenylalanine	C10H13NO2	179.22	1.19	180.10	Amino Acid And Its Derivatives	67,450	39,870	17,750	95,420	55,730	80,600	60,870	39,800	44,360
Com_42_neg	Dehydrodiisoeugenol	20H22O4	326.39	5.44	325.15	Phenols	191,900	7,153	20,280	25,064	22,671	24,856	23,020	21,230	19,030
Com_742_pos	delta-Aminolevulinic acid hydrochloride	C5H10ClNO3	167.59	1.17	162.10	Carbohydrates	806,300	2,089,000	1,833,000	333,400	286,700	297,600	548,200	551,000	504,100
Com_736_pos	Dendrobine	C16H25NO2	263.38	18.28	264.20	Alkaloids	8,990	5,688	6,454	8,484	6,654	6,671	7,682	7,268	8,269
Com_358_pos	Denin	C33H56O14	676.79	13.46	694.40	Flavonoids	12,840	14,850	3,444	71,070	65,390	59,220	54,930	57,040	48,550
Com_359_pos	Deoxyadenosine	C10H13N5O3	251.24	9.28	252.00	Nucleotide And Its Derivates	36,220	9,115	9,273	27,080	29,214	27,582	5,447	3,737	6,850
Com_357_pos	Deoxyguanosine	C10H13N5O4	267.24	0.99	268.10	Nucleotide And Its Derivates	378,800	211,600	193,300	150,600	123,500	117,900	227,800	187,400	141,000

Com_231_neg	deoxyribose-phosphate			0.65	213.00	Nucleotide And Its Derivates	10,180	4,342	4,379	3,250	1,968	6,404	13,900	16,270	20,240
Com_230_neg	D-Erythrose 4-phosphate	C4H9O7P	200.08	0.61	199.00	Carbohydrates	65,190	30,930	30,670	79,690	76,770	91,750	54,750	37,800	55,650
Com_229_neg	D-Fructose 1,6-bisphosphate	C6H14O12P2	340.12	0.50	338.99	Carbohydrates	171,100	179,200	123,100	119,200	117,400	104,500	312,200	327,900	332,000
Com_174_neg	D-Fructose 6-phosphate	C6H13O9P	260.03	0.64	259.00	Carbohydrates	531,900	162,400	210,500	156,100	126,300	114,600	228,100	254,900	233,800
Com_139_neg	dGDP	C10H15N5O10P2	427.20	0.68	426.00	Nucleotide And Its Derivates	32,590	24,530	19,010	31,530	14,030	22,240	24,210	14,970	17,700
Com_141_neg	D-Gluconic acid	C6H12O7	196.16	0.63	195.10	Carbohydrates	170,100	119,000	45,330	236,200	196,300	206,400	104,300	111,100	101,500
Com_415_pos	D-Glucosamine	C6H13NO5	179.17	0.63	180.00	Carbohydrates	1,687,000	218,400	108,600	280,100	277,700	381,500	862,800	645,600	509,000
Com_28_neg	D-Glucose	C6H12O6	180.16	0.79	179.06	Carbohydrates	1,004,000	867,200	936,800	1,746,000	1,870,000	1,838,000	955,300	928,500	961,900
Com_129_neg	D-Glucose 1-phosphate	C6H13O9P	260.14	0.65	259.01	Carbohydrates	343,800	448,100	510,500	181,700	109,000	167,200	187,500	282,400	289,500
Com_158_neg	D-Glucose 6-phosphate	C6H13O9P	260.14	0.63	259.00	Carbohydrates	2,493,000	711,200	1,388,000	442,800	486,700	465,400	456,500	523,800	517,800
Com_143_neg	D-Glucuronic acid	C6H10O7	194.14	0.54	193.00	Carbohydrates	824,000	1,332,000	3,063,000	992,000	989,300	1,137,000	724,000	686,500	741,400
Com_142_neg	D-Glutamic acid	C5H9NO4	147.13	0.73	148.07	Amino Acid And Its Derivatives	72,840	170,800	84,880	39,590	47,970	60,060	45,310	39,410	34,380
Com_669_pos	D-Glutamine	C5H10N2O3	146.15	0.65	147.10	Amino Acid And Its Derivatives	#########	#########	#########	#########	#########	#########	#########	#########	#########
Com_140_neg	dGMP	C10H14N5O7P	347.22	0.68	346.10	Nucleotide And Its Derivates	131,400	274,200	222,100	313,800	345,200	187,300	206,300	260,900	265,900
Com_362_pos	di-C,C-hexosyl-apigenin	C27H30O15	594.52	4.28	595.17	Flavonoids	6,377,000	509,900	316,100	788,600	727,300	759,800	4,884,000	5,306,000	5,664,000
Com_360_pos	di-C,C-hexosyl-apigenin derivative	C27H30O15	594.52	5.02	595.17	Flavonoids	131,200	13,550	108,500	40,300	28,370	33,010	123,300	92,310	97,080
Com_361_pos	di-C,C-hexosyl-methylluteolin	C28H32O16	624.54	4.59	625.18	Flavonoids	979,700	342,200	201,400	1,936,000	1,753,000	1,773,000	732,500	793,300	905,100
Com_477_pos	Diethanolamine	C4H11NO2	105.14	0.73	106.00	Amino Acid And Its Derivatives	229,800	859,300	861,400	178,400	143,100	179,700	378,300	335,000	355,500
Com_202_neg	Dihydrodehydrodiconiferyl Alcohol	C20H24O6	360.40	14.14	359.15	Others	19,830	1,392	6,468	4,670	2,232	5,085	11,910	11,160	13,640
Com_537_pos	Diosgenin	C27H42O3	414.62	13.04	414.90	Terpenoids	1,805,000	7,435,000	7,431,000	1,423,000	1,509,000	1,560,000	2,225,000	2,663,000	2,603,000
Com_538_pos	Diosmetin	C16H12O6	300.26	8.55	301.07	Flavonoids	91,330	16,260	47,680	148,100	125,000	131,300	36,740	40,310	42,400
Com_91_neg	Dipsacoside B	C53H86O22	1075.24	9.26	1045.14	Terpenoids	889,100	20,570	14,850	135,300	123,100	124,700	187,500	195,400	212,300
Com_539_pos	Dl-2-Aminooctanoic Acid	C8H17NO2	159.23	0.74	160.10	Organic Acid And Its Derivatives	1,936,000	1,194,000	1,011,000	2,193,000	1,880,000	1,873,000	1,114,000	1,109,000	1,004,000
Com_252_neg	Dl-Glyceraldehyde3-phosphate	C3H7O6P	170.00	0.62	169.00	Others	3,245	3,569	3,155	3,175	4,428	7,600	4,369	3,172	2,780
Com_639_pos	DL-Pipecolinic acid	C6H11NO2	129.16	0.75	140.15	Organic Acid And Its Derivatives	#########	#########	#########	#########	#########	#########	#########	#########	#########
Com_541_pos	DL-threo-beta-Methylaspartic acid	C5H9NO4	147.13	0.74	148.10	Amino Acid And Its Derivatives	14,530	29,060	19,840	10,970	5,285	10,540	17,300	11,710	13,480
Com_154_neg	D-Malic acid	C4H6O5	134.09	0.63	133.01	Organic Acid And Its Derivatives	#########	#########	#########	#########	#########	#########	#########	#########	#########
Com_90_neg	D-Mannose 6-phosphate	C6H13O9P	260.14	0.53	259.00	Carbohydrates	653,700	158,500	291,100	163,100	138,300	160,600	130,600	140,800	121,300
Com_535_pos	D-Methionine	C5H11NO2S	149.21	0.91	150.10	Amino Acid And Its Derivatives	8,800	4,012,000	5,037,000	258,600	203,900	240,100	588,200	520,400	384,600

Com_536_pos	D-Norvaline	C5H11NO2	117.15	0.90	118.00	Amino Acid And Its Derivatives	#########	#########	#########	#########	#########	#########	#########	#########	#########
Com_250_neg	Docetaxel	C43H53NO14	807.88	0.69	852.35	Terpenoids	3,595	6,819	5,237	2,322	1,963	2,440	5,100	2,416	3,386
Com_667_pos	D-Proline	C5H9NO2	115.13	0.79	116.00	Amino Acid And Its Derivatives	#########	#########	#########	8,630,000	8,081,000	8,014,000	#########	#########	#########
Com_666_pos	Dracorhodin perochlorate	C17H15ClO7	366.75	8.60	367.06	Others	199,700	19,410	5,172	95,490	94,510	102,200	54,210	55,780	54,700
Com_234_neg	D-Ribulose 5-phosphate	C5H11O8P	230.02	0.60	229.02	Carbohydrates	135,200	65,500	64,050	47,190	53,210	86,590	85,430	62,550	84,870
Com_94_neg	D-Sedoheptulose 7-phosphate	C7H13BaO10P	425.47	0.52	389.03	Carbohydrates	211,000	103,000	100,700	125,200	153,300	274,800	114,500	112,200	123,200
Com_232_neg	D-sedoheptulose-1-7-phosphate			0.62	289.00	Carbohydrates	159,500	105,100	96,060	231,300	182,900	210,100	121,500	99,400	126,700
Com_665_pos	D-Sorbitol	C6H14O6	182.17	0.61	183.00	Carbohydrates	55,070	28,610	16,530	85,130	82,280	82,800	68,710	48,380	44,580
Com_92_neg	D-Tagatose	C6H12O6	180.16	0.62	179.06	Carbohydrates	900,400	716,200	499,800	2,177,000	2,285,000	2,243,000	1,011,000	891,500	1,066,000
Com_674_pos	dUDP	C9H14N2O11P2	388.16	11.75	388.10	Nucleotide And Its Derivates	299,200	2,231,000	2,211,000	304,700	298,000	277,800	386,000	510,000	490,400
Com_93_neg	Dulcitol	C6H14O6	182.17	0.69	181.07	Carbohydrates	940,200	300,700	40,160	145,000	192,600	241,600	1,157,000	1,017,000	2,238,000
Com_61_neg	D-Xylonic acid	C5H10O6	166.05	0.69	165.05	Organic Acid And Its Derivatives	4,360,000	1,517,000	1,948,000	7,126,000	7,583,000	8,250,000	4,045,000	3,784,000	3,583,000
Com_97_neg	D-Xylose	C5H10O5	150.13	0.72	149.05	Carbohydrates	226,000	192,100	179,800	759,100	872,200	789,000	399,800	365,300	386,100
Com_98_neg	D-Xylulose	C5H10O5	150.13	0.72	149.03	Carbohydrates	184,000	129,700	133,400	501,600	523,600	495,900	454,900	398,400	404,300
Com_34_neg	D-Xylulose 5-phosphate	C5H11O8P	230.11	0.70	265.00	Carbohydrates	21,490	20,000	34,920	26,190	23,710	20,300	24,480	10,850	17,020
Com_48_neg	E-3,4,5'-Trihydroxy-3'-glucopyranosylstilbene			10.70	405.10	Others	63,350	21,670	39,320	61,490	61,580	57,650	158,200	144,200	154,800
Com_57_neg	Ellagic acid	C14H6O8	302.19	3.41	301.00	Phenols	304,900	15,560	19,260	37,700	37,980	37,960	55,030	53,960	57,170
Com_49_neg	Engeletin	C21H22O10	434.39	7.16	433.11	Flavonoids	50,280	6,679	16,710	21,754	21,061	21,476	12,020	16,860	16,410
Com_730_pos	Epigoitrin	C5H7NOS	129.18	0.77	130.03	Others	2,581,000	3,387,000	2,352,000	2,376,000	2,188,000	2,114,000	3,861,000	3,067,000	2,899,000
Com_469_pos	epsilon-Viniferin	C28H22O6	454.47	9.86	455.15	Others	26,847	10,475	6,780	47,150	47,270	41,500	5,506	9,430	8,019
Com_296_neg	Eriodictyol	C15H12O6	288.25	7.73	287.06	Flavonoids	13,110	26,659	26,860	92,540	78,410	86,040	32,520	25,840	28,700
Com_297_neg	Erucic acid	C22H42O2	338.57	20.25	337.31	Lipids	#########	#########	#########	#########	#########	#########	#########	#########	#########
Com_295_neg	Esculin hydrate	C15H16O9	340.28	3.68	339.07	Phenylpropanoids	558,800	144,700	87,480	87,100	92,320	99,980	66,170	78,250	86,600
Com_526_pos	Ethyl ferulate	C12H14O4	222.24	29.31	223.10	Phenols	138,800	138,200	153,700	150,900	117,400	131,100	181,000	153,800	138,600
Com_427_pos	Ferulic Acid	C10H10O4	194.18	3.41	195.06	Phenols	1,966,000	242,700	114,000	736,200	682,400	713,100	281,400	324,800	311,000
Com_735_pos	Feruloylputrescine	C14H20N2O3	264.32	3.39	265.16	Phenols	47,090	35,050	24,860	95,750	80,970	77,950	131,800	93,230	137,900
Com_167_neg	Flavin Adenine Dinucleotide	C27H33N9O15P2	785.55	5.76	784.20	Nucleotide And Its Derivates	6,750	9,972	4,896	41,700	36,510	39,060	17,800	22,030	25,250
Com_166_neg	Formononetin	C16H12O4	268.26	11.40	267.00	Flavonoids	13,494	7,618	6,800	2,408	2,440	2,361	2,802	2,100	2,701
Com_128_neg	Fructose	C6H12O6	180.06	0.74	178.80	Carbohydrates	2,983,000	521,700	513,200	2,331,000	2,505,000	2,090,000	1,351,000	1,199,000	1,219,000
Com_71_neg	Fumaric Acid	C4H4O4	116.07	0.56	115.00	Organic Acid And Its Derivatives	940,300	454,200	327,900	1,814,000	1,594,000	1,878,000	1,082,000	758,000	765,700
Com_510_pos	Fustin	C15H12O6	288.25	4.90	289.00	Flavonoids	134,700	101,000	100,700	76,833	75,003	67,998	32,990	25,240	25,910
Com_70_neg	GA7-1			11.37	329.10	Organic Acid And Its Derivatives	2,568,000	204,700	275,200	205,100	184,600	183,000	968,500	1,050,000	1,065,000
Com_239_neg	Gallocatechin-catechin		594.10	8.44	593.10	Organic Acid And Its Derivatives	19,620	59,680	25,040	64,890	60,770	63,010	18,180	23,350	24,040

Com_465_pos	gamma-Aminobutyric acid	C4H9NO2	103.12	0.69	104.10	Organic Acid And Its Derivatives	675,500	1,506,000	1,040,000	1,231,000	1,100,000	1,209,000	952,000	806,900	840,100
Com_721_pos	gamma-Dodecalactone	C12H22O2	198.30	1.47	199.20	Others	119,000	36,124	37,793	17,260	23,570	12,000	20,700	21,940	20,960
Com_281_neg	GDP-alpha-D-glucose	C16H25N5O16P2	637.34	0.66	626.05	Nucleotide And Its Derivates	38,516	7,934	5,814	12,520	10,340	13,620	14,780	6,980	5,311
Com_520_pos	GDP-L-fucose	C16H25N5O15P2	589.34	0.80	590.09	Nucleotide And Its Derivates	16,800	337	1,694	11,220	9,148	4,386	11,610	7,133	7,889
Com_456_pos	Gentiobiose	C12H22O11	342.30	0.60	365.10	Carbohydrates	213,500	77,890	78,230	119,200	108,300	141,000	98,560	90,020	81,780
Com_413_pos	Germacrone;(3E,7E)-3,7-dimethyl-10-propan-2-ylidenecyclodeca-3,7-dien-1-one	C15H22O	218.34	14.93	219.17	Others	22,410	14,994	19,939	33,048	34,096	31,947	12,950	13,110	8,974
Com_260_neg	Ginsenoside C-K	C36H62O8	622.87	0.76	667.44	Terpenoids	22,800	12,370	9,425	10,640	5,944	7,315	6,232	5,538	8,930
Com_258_neg	Glucosylvitexin	C27H30O15	594.52	4.84	593.15	Flavonoids	1,512,000	123,100	78,380	221,500	206,600	229,000	1,972,000	2,114,000	2,184,000
Com_414_pos	Glu-Glu	C10H14N2O7	276.24	0.82	277.10	Amino Acid And Its Derivatives	65,010	19,180	17,860	48,460	37,040	45,620	34,710	37,130	42,200
Com_282_neg	Glycitin	C22H22O10	446.40	8.24	445.10	Flavonoids	16,070	73,564	46,509	32,770	33,490	31,740	98,970	107,200	104,100
Com_733_pos	Glycyl-L-Leucine	C8H16N2O3	188.22	1.30	189.10	Amino Acid And Its Derivatives	320,400	82,610	65,880	114,300	112,800	129,800	159,600	141,900	119,300
Com_284_neg	GMP	C10H14N5O8P	363.22	0.62	362.10	Nucleotide And Its Derivates	44,650	33,520	28,510	27,640	28,450	25,940	35,250	25,910	34,280
Com_283_neg	Guanidinoethyl Sulfonate	C3H9N3O3S	167.19	0.72	166.03	Organic Acid And Its Derivatives	149,600	7,653	7,252	19,170	8,405	11,720	70,280	69,730	45,830
Com_475_pos	Guanosine	C10H13N5O5	283.24	0.91	284.10	Nucleotide And Its Derivates	501,000	307,700	252,700	387,900	258,500	285,600	232,500	221,900	242,300
Com_519_pos	Guanosine 3',5'-Cyclic Monophosphate	C10H12N5O7P	345.21	0.89	346.05	Nucleotide And Its Derivates	92,290	64,970	90,260	153,400	118,800	146,500	47,880	55,660	38,560
Com_161_neg	Guanosine 5'-triphosphate	C10H16N5O14P3	523.18	0.68	522.00	Nucleotide And Its Derivates	12,230	7,515	7,115	2,513	5,197	8,524	4,805	4,125	4,735
Com_190_neg	Helicid	C13H16O7	284.26	9.88	283.08	Phenols	10,334	5,520	4,777	2,457	6,876	7,895	2,764	2,941	1,035
Com_191_neg	Heptamethoxyflavone	C22H24O9	432.42	5.00	431.10	Flavonoids	25,447	5,865	4,988	5,560	8,109	3,993	8,641	5,683	5,068
Com_518_pos	Hesperetin 5-O-glucoside	C22H24O11	464.42	6.52	465.10	Flavonoids	1,337,000	4,836,000	2,314,000	360,600	304,200	316,100	499,400	538,300	570,800
Com_264_neg	Hesperidin	C28H34O15	610.19	7.50	609.19	Flavonoids	1,038,000	2,750,000	3,560,000	157,300	122,500	130,900	473,600	481,500	525,700
Com_422_pos	Hippuric Acid	C9H9NO3	179.17	2.87	180.10	Amino Acid And Its Derivatives	82,750	19,820	16,000	45,880	47,080	47,400	33,090	40,510	33,470
Com_426_pos	Histamine	C5H9N3	111.15	0.79	112.10	Amino Acid And Its Derivatives	1,965,000	654,700	561,200	754,200	721,500	726,300	1,000,000	907,600	857,900
Com_423_pos	Histidine	C6H9N3O2	155.16	0.61	156.08	Amino Acid And Its Derivatives	257,700	1,479,000	1,178,000	574,600	475,800	550,900	483,000	467,500	385,900
Com_302_neg	Homocysteine	C4H9NO2S	134.19	0.70	133.90	Amino Acid And Its Derivatives	70,630	82,020	3,948	108,300	322,000	190,200	26,880	20,940	12,250
Com_192_neg	Homogentisic Acid	C8H8O4	168.15	0.84	167.10	Organic Acid And Its Derivatives	61,790	31,760	10,590	56,330	70,540	57,310	22,140	18,410	20,370

Com_430_pos	Hordenine	C10H15NO	165.23	1.61	166.12	Alkaloids	#########	#########	#########	5,201,000	4,846,000	4,606,000	#########	#########	#########
Com_189_neg	Hydroxycitric acid	C6H8O8	208.12	0.63	207.02	Organic Acid And Its Derivatives	160,500	55,750	60,070	138,500	136,900	130,700	105,700	86,090	96,470
Com_399_pos	Hypoxanthine	C5H4N4O	136.11	7.63	137.00	Nucleotide And Its Derivates	42,120	50,970	53,870	43,000	37,450	41,100	57,660	32,740	47,470
Com_715_pos	Hypoxanthine-9-β-D-Arabinofuranoside	C10H12N4O5	268.23	0.83	269.10	Nucleotide And Its Derivates	7,226,000	4,737,000	3,514,000	4,075,000	3,978,000	4,136,000	2,491,000	3,247,000	2,793,000
Com_716_pos	IAA-Asp			4.81	291.30	Amino Acid And Its Derivatives	13,010	4,610	7,552	15,760	17,260	22,360	18,880	16,640	26,690
Com_714_pos	IAA-Glu			0.56	305.10	Amino Acid And Its Derivatives	68,660	37,550	33,180	112,000	122,600	130,600	65,200	80,970	84,920
Com_632_pos	IBA	C12H13NO2	203.24	8.18	204.10	Amino Acid And Its Derivatives	52,080	7,821	6,727	13,860	22,164	19,820	12,330	30,040	23,710
Com_392_pos	Indole-3-acetic acid	C10H9NO2	175.18	5.66	176.07	Amino Acid And Its Derivatives	177,600	93,350	86,230	30,390	47,440	62,170	41,880	62,610	64,050
Com_542_pos	Indole-3-Carboxaldehyde	C9H7NO	145.16	2.64	146.10	Amino Acid And Its Derivatives	247,300	1,236,000	1,072,000	281,100	250,600	259,400	278,000	310,500	275,100
Com_590_pos	Indole-3-carboxylic acid	C9H7NO2	161.16	5.87	162.15	Alkaloids	31,450	20,000	19,640	13,990	12,830	11,870	48,680	52,780	35,180
Com_26_neg	Indole-5-carboxylic acid	C9H7NO2	161.05	0.90	160.05	Amino Acid And Its Derivatives	89,330	116,000	79,240	55,260	50,720	56,060	45,030	22,240	37,240
Com_589_pos	Inosine-5'-monophosphate	C10H13N4O8P	348.21	0.78	349.10	Nucleotide And Its Derivates	71,630	217,000	146,300	186,500	182,100	93,330	95,490	143,300	139,900
Com_25_neg	Inositol	C6H12O6	180.16	0.72	179.16	Carbohydrates	1,052,000	2,571,000	815,700	3,782,000	4,651,000	4,900,000	2,246,000	2,250,000	2,291,000
Com_69_neg	Isoacteoside	C29H36O15	624.59	8.66	623.20	Phenylpropanoids	165,700	41,280	137,400	40,070	31,960	34,250	30,440	18,430	30,750
Com_80_neg	IsochlorogenicacidC	C25H24O12	516.46	0.90	515.12	Phenols	772,700	5,809,000	3,841,000	2,165,000	1,718,000	2,327,000	1,931,000	1,692,000	2,033,000
Com_68_neg	Isocitrate	C6H8O7	192.12	0.48	191.00	Organic Acid And Its Derivatives	#########	3,692,000	3,794,000	#########	#########	#########	8,623,000	7,219,000	8,615,000
Com_113_neg	Isomucronulatol-7-O-glucoside	C23H28O10	464.46	6.52	463.16	Flavonoids	#########	#########	#########	3,695,000	3,301,000	3,099,000	5,580,000	5,408,000	4,990,000
Com_726_pos	Isoorientin	C21H20O11	448.38	5.24	449.11	Flavonoids	2,070,000	112,600	140,300	350,500	328,400	339,000	322,500	361,300	353,800
Com_110_neg	Isorhamnetin	C16H12O7	316.26	10.02	315.05	Flavonoids	12,700	32,078	5,778	466,700	405,200	449,700	79,660	79,860	79,850
Com_109_neg	Isorhoifolin	C27H30O14	578.52	9.29	577.10	Flavonoids	11,180	24,390	35,990	50,320	48,280	37,410	14,540	17,040	19,280
Com_576_pos	Isorhynchophylline	C22H28N2O4	384.47	7.46	385.21	Alkaloids	67,500	13,771	1,350	61,980	43,110	58,870	20,830	22,670	20,440
Com_108_neg	Isoschaftoside	C26H28O14	564.49	5.43	563.14	Flavonoids	855,500	209,000	55,800	448,900	374,800	413,700	684,200	704,300	734,200
Com_397_pos	Isotanshinone IIA	C19H18O3	294.34	15.10	295.10	Others	379,600	70,030	104,700	141,800	120,700	123,000	261,000	286,200	273,100
Com_148_neg	Isotrifoliin	C21H20O12	464.10	7.40	463.00	Flavonoids	#########	#########	#########	3,919,000	3,204,000	2,943,000	7,638,000	4,783,000	4,899,000
Com_214_neg	Isovitexin	C21H20O10	432.38	6.93	431.10	Flavonoids	#########	2,761,000	#########	3,049,000	2,993,000	2,680,000	#########	#########	#########
Com_215_neg	JA-Ile			11.79	322.10	Organic Acid And Its Derivatives	127,500	365,400	162,800	162,400	152,300	153,900	271,100	249,200	243,200
Com_212_neg	Kaempferide	C16H12O6	300.26	12.80	299.00	Flavonoids	102,900	16,940	3,007	#########	#########	#########	955,900	1,017,000	1,026,000
Com_130_neg	Kaempferin	C21H20O10	432.11	9.13	431.00	Flavonoids	18,950	14,310	8,419	26,160	26,080	24,980	9,265	7,545	7,766
Com_461_pos	Kaempferitrin	C27H30O14	578.52	5.84	579.17	Flavonoids	47,970	8,264	24,423	52,040	38,500	37,120	89,620	72,110	69,980
Com_355_pos	Kaempferol 3-O-glucoside-2‘-O-rhamnoside			5.29	595.30	Flavonoids	949,100	1,607,000	642,400	116,700	165,500	136,600	209,000	188,900	184,300

Com_157_neg	Kaempferol 3-O-robinobioside (Biorobin)	C27H30O15	594.52	8.46	593.16	Flavonoids	569,800	2,098,000	979,600	3,009,000	2,632,000	2,633,000	714,200	781,800	838,000
Com_607_pos	Kaempferol-3-O-rutinoside	C27H30O15	594.52	5.49	595.17	Flavonoids	96,380	107,071	106,232	217,100	223,000	237,900	92,360	59,390	78,950
Com_354_pos	Kaempferol7-O-β-D-glucopyranoside	C21H20O11	448.38	5.49	449.11	Flavonoids	1,258,000	1,629,000	730,300	242,400	243,800	263,000	259,900	286,300	238,700
Com_14_neg	Kaji-Ichigoside F1	C36H58O10	650.84	6.97	649.40	Others	878,100	555,600	938,000	24,897	23,818	24,343	90,760	76,040	91,230
Com_15_neg	Kinsenoside	C10H16O8	264.23	0.89	309.08	Others	331,700	308,400	247,100	784,600	917,900	747,700	482,900	527,800	492,000
Com_662_pos	Kolavic acid	C20H30O4	334.45	14.54	335.22	Terpenoids	9,379	6,545	2,717	7,842	12,980	17,690	14,670	10,890	10,880
Com_33_neg	Kynurenic acid	C10H7NO3	189.17	4.52	188.04	Organic Acid And Its Derivatives	122,200	15,480	6,503	12,240	9,902	9,788	49,430	29,120	64,740
Com_138_neg	Kynurenic acid O-hexside	C16H17NO8	351.10	2.74	350.10	Organic Acid And Its Derivatives	15,601	7,959	7,610	9,392	9,665	9,571	4,300	4,276	5,544
Com_12_neg	L-3-Phenyllactic Acid	C9H10O3	166.17	0.74	165.10	Organic Acid And Its Derivatives	72,920	17,890	34,550	68,550	84,570	90,000	47,640	34,370	41,150
Com_125_neg	Lactic acid	C3H6O3	90.08	0.72	89.00	Organic Acid And Its Derivatives	21,980	3,562	2,776	15,460	33,250	34,820	6,740	5,975	2,764
Com_10_neg	Lactobionic acid	C12H22O12	358.30	0.59	357.10	Carbohydrates	#########	#########	#########	#########	#########	#########	#########	#########	#########
Com_731_pos	lactucopicrin	C23H22O7	410.42	12.20	411.14	Others	5,771	14,340	8,632	3,950	6,778	4,655	16,640	11,980	21,600
Com_660_pos	L-Alanine	C3H7NO2	89.09	0.81	90.00	Amino Acid And Its Derivatives	3,037,000	4,653,000	1,839,000	1,816,000	1,613,000	1,626,000	2,136,000	2,147,000	1,880,000
Com_64_neg	L-Arabinose	C5H10O5	150.05	0.71	149.00	Carbohydrates	677,900	531,100	441,100	2,378,000	2,731,000	2,757,000	1,081,000	1,152,000	1,180,000
Com_88_neg	L-Arabitol	C5H12O5	152.15	0.71	151.06	Carbohydrates	270,500	44,420	20,240	78,500	100,100	89,330	393,900	263,700	271,700
Com_11_neg	L-Arginine	C6H14N4O2	174.20	0.75	173.10	Amino Acid And Its Derivatives	13,190	8,527	6,041	10,460	19,730	8,885	8,442	4,826	5,233
Com_314_neg	L-Argininosuccinate	C10H18N4O6	290.27	0.57	289.10	Organic Acid And Its Derivatives	162,600	97,010	120,000	165,600	105,800	172,200	117,400	171,400	107,700
Com_107_neg	Laricitrin	C16H12O8	332.26	1.70	331.00	Flavonoids	3,355,000	228,600	247,100	2,604,000	2,853,000	2,542,000	1,072,000	1,140,000	1,181,000
Com_661_pos	L-Ascorbate	C6H8O6	176.12	12.28	177.00	Terpenoids	2,328,000	2,306,000	2,238,000	2,136,000	2,087,000	2,134,000	2,006,000	2,346,000	2,019,000
Com_658_pos	L-Asparagine	C4H8N2O3	132.12	0.77	133.00	Amino Acid And Its Derivatives	7,149,000	#########	#########	3,133,000	2,972,000	2,842,000	#########	#########	#########
Com_664_pos	L-Citrulline	C6H13N3O3	175.19	0.74	176.10	Amino Acid And Its Derivatives	125,300	599,100	318,400	469,200	421,700	462,400	387,900	356,200	351,900
Com_533_pos	L-Cystine	C6H12N2O4S2	240.30	1.06	241.00	Amino Acid And Its Derivatives	1,564,000	1,291,000	923,500	3,538,000	3,014,000	3,056,000	1,717,000	1,450,000	1,476,000
Com_534_pos	Levistilide A	C24H28O4	380.48	15.87	381.21	Terpenoids	10,670	10,170	16,260	13,230	14,690	17,830	7,480	11,430	12,660
Com_634_pos	Levodopa	C9H11NO4	197.19	0.78	198.10	Alkaloids	491,700	9,327,000	7,368,000	340,100	393,800	426,400	1,481,000	1,158,000	1,294,000
Com_7_neg	L-Fucose	C6H12O5	164.07	0.83	163.00	Carbohydrates	40,880	23,910	26,030	44,130	61,460	58,810	26,440	31,640	28,820
Com_531_pos	L-Glutamate	C5H9NO4	147.13	0.81	148.10	Amino Acid And Its Derivatives	7,320,000	#########	#########	4,302,000	3,869,000	4,073,000	#########	9,871,000	9,833,000
Com_6_neg	L-Gulonic acid gamma-lactone	C6H10O6	178.05	0.78	177.14	Carbohydrates	30,920	5,231	7,256	18,820	19,290	27,980	10,180	15,040	8,167
Com_424_pos	L-Homocarnosine	C10H16N4O3	240.26	0.64	241.30	Amino Acid And Its Derivatives	587,800	318,900	220,300	1,071,000	1,114,000	1,260,000	415,600	578,200	545,600

Com_425_pos	L-Homocystine	C8H16N2O4S2	268.35	0.90	269.00	Amino Acid And Its Derivatives	4,857,000	3,548,000	2,445,000	2,874,000	2,868,000	3,064,000	1,881,000	2,455,000	2,154,000
Com_177_neg	L-Homoserine	C4H9NO3	119.06	0.73	118.10	Amino Acid And Its Derivatives	16,390	15,980	19,580	4,009	4,008	5,606	15,580	8,800	5,605
Com_349_pos	Linthospermic Acid +Na	C27H22O12	538.46	6.98	561.10	Phenols	8,729	23,340	21,860	69,940	58,130	51,960	17,090	28,040	23,740
Com_577_pos	Lipoamide	C8H15NOS2	205.34	2.57	206.20	Amino Acid And Its Derivatives	173,000	861,600	694,100	188,900	170,200	172,300	176,600	190,700	204,300
Com_352_pos	L-Isoleucine	C6H13NO2	131.17	1.23	132.00	Amino Acid And Its Derivatives	#########	#########	#########	#########	#########	#########	#########	#########	#########
Com_351_pos	L-Leucine	C6H13NO2	131.17	1.23	132.10	Amino Acid And Its Derivatives	#########	#########	#########	#########	#########	#########	#########	#########	#########
Com_511_pos	L-Methionine sulfone	C5H11NO4S	181.21	0.78	182.00	Amino Acid And Its Derivatives	266,000	898,700	846,700	157,200	158,900	138,400	267,400	263,700	280,900
Com_470_pos	L-Mimosine	C8H10N2O4	198.18	0.71	199.07	Lipids	49,000	17,640	7,257	24,560	41,930	37,730	15,100	20,390	18,140
Com_163_neg	Lonicerin	C27H30O15	594.52	7.67	593.15	Flavonoids	5,231,000	7,679,000	4,984,000	869,100	869,300	862,200	1,428,000	1,502,000	1,486,000
Com_471_pos	L-Ornithine	C5H13ClN2O2	132.16	0.70	133.00	Amino Acid And Its Derivatives	528,200	1,263,000	1,158,000	279,600	203,600	204,500	880,200	799,600	872,000
Com_473_pos	LPC(1-acyl 16:0)	C24H50NO7P	495.63	15.06	496.34	Lipids	193,100	351,200	649,600	1,368,000	1,204,000	1,272,000	61,210	75,790	63,310
Com_472_pos	LPC(1-acyl 16:1)	C24H48NO7P	493.61	14.12	494.32	Lipids	39,020	10,040	3,743	17,580	14,670	11,400	9,906	11,060	10,010
Com_474_pos	LPC(1-acyl 18:1)	C26H52NO7P	521.67	15.29	522.36	Lipids	36,950	52,170	22,810	107,300	106,600	52,830	120,200	139,100	126,100
Com_464_pos	LPC(1-acyl 18:2)	C26H50NO7P	519.65	14.52	520.34	Lipids	1,063,000	636,500	480,900	3,002,000	2,773,000	2,230,000	928,800	1,018,000	965,900
Com_651_pos	L-Phenylalanine	C9H11NO2	165.19	1.53	166.10	Amino Acid And Its Derivatives	#########	#########	#########	#########	#########	#########	#########	#########	#########
Com_587_pos	L-Pipecolate	C6H11NO2	129.16	0.73	130.00	Amino Acid And Its Derivatives	#########	#########	#########	8,053,000	6,993,000	7,146,000	#########	#########	#########
Com_67_neg	L-Saccharopine	C11H20N2O6	276.13	0.72	275.00	Amino Acid And Its Derivatives	1,824,000	1,645,000	1,915,000	2,096,000	2,095,000	1,995,000	1,874,000	1,830,000	2,000,000
Com_584_pos	L-Serine	C3H7NO3	105.09	0.81	106.00	Amino Acid And Its Derivatives	2,843,000	7,425,000	8,489,000	1,594,000	1,501,000	1,514,000	3,529,000	3,555,000	3,255,000
Com_63_neg	L-Tartaric acid	C4H6O6	150.02	0.64	149.00	Organic Acid And Its Derivatives	146,400	102,000	69,630	472,600	458,700	502,700	199,100	194,700	206,500
Com_585_pos	L-Theanine	C7H14N2O3	174.20	0.72	175.11	Amino Acid And Its Derivatives	730,400	2,129,000	1,337,000	1,138,000	1,039,000	1,062,000	1,428,000	1,288,000	1,122,000
Com_586_pos	L-Tryptophan	C11H12N2O2	204.23	2.67	205.10	Amino Acid And Its Derivatives	#########	#########	#########	#########	#########	#########	#########	#########	#########
Com_287_neg	L-Tyrosine	C9H11NO3	181.19	1.31	180.00	Amino Acid And Its Derivatives	1,138,000	2,154,000	1,821,000	191,100	387,400	181,900	372,500	260,900	281,200
Com_165_neg	Lumichrome	C12H10N4O2	242.23	10.00	241.07	Alkaloids	24,750	56,510	32,360	456,700	384,900	381,700	185,200	162,500	109,700
Com_31_neg	Luteolin 7-O-glucoside	C21H20O11	448.38	8.51	447.09	Flavonoids	167,600	160,500	151,300	23,240	16,530	23,000	43,960	41,660	45,990
Com_713_pos	L-Valine	C5H11NO2	117.15	0.79	118.09	Amino Acid And Its Derivatives	1,437,000	4,573,000	4,380,000	553,900	482,500	509,500	1,278,000	1,343,000	1,172,000

Com_555_pos	Lycoperodine	C12H12N2O2	216.24	3.75	217.20	Amino Acid And Its Derivatives	#########	7,842,000	4,596,000	#########	8,763,000	9,154,000	#########	#########	#########
Com_298_neg	LysoPA 18:0 (2n isomer)	C21H35O7P	438.27	0.81	437.27	Lipids	19,880	22,320	24,130	21,660	11,750	12,800	23,300	16,860	10,130
Com_156_neg	Malate	C4H6O5	134.09	0.63	133.00	Organic Acid And Its Derivatives	#########	#########	#########	#########	#########	#########	#########	#########	#########
Com_89_neg	Malic acid	C4H6O5	134.09	0.56	133.00	Organic Acid And Its Derivatives	#########	9,733,000	2,731,000	#########	#########	#########	#########	#########	#########
Com_259_neg	Malonicacid	C3H4O4	104.06	0.57	103.00	Organic Acid And Its Derivatives	123,400	8,324	7,525	13,090	10,690	13,310	21,400	12,280	6,348
Com_722_pos	Maltitol	C12H24O11	344.31	0.71	345.14	Carbohydrates	187,600	90,650	89,790	190,400	159,100	156,700	101,200	102,900	91,830
Com_137_neg	Maltotetraose	C24H42O21	666.10	0.87	665.10	Carbohydrates	43,480	20,906	19,689	34,020	58,920	40,760	11,040	11,330	5,736
Com_723_pos	Maltotriitol	C18H34O16	506.45	0.71	529.18	Carbohydrates	314,900	319,200	404,800	260,200	287,900	243,200	258,600	203,700	231,900
Com_36_neg	Maslinic acid	C30H48O4	472.70	16.26	471.30	Terpenoids	844,500	166,800	242,105	732,100	564,500	388,700	389,100	405,100	394,600
Com_727_pos	Matairesinol	C20H22O6	358.39	6.92	359.15	Phenols	27,250	6,653	1,079	31,741	27,767	26,629	22,410	15,890	21,930
Com_670_pos	Melezitose	C18H32O16	504.44	0.72	527.16	Carbohydrates	4,334,000	7,891,000	#########	4,402,000	5,007,000	4,009,000	4,563,000	4,031,000	4,413,000
Com_506_pos	Methional	C4H8OS	104.17	0.77	105.00	Others	1,717,000	1,613,000	2,038,000	1,153,000	1,043,000	1,116,000	1,184,000	1,038,000	986,100
Com_420_pos	Methyl arachidonate	C21H34O2	318.49	19.58	319.26	Lipids	9,871	9,355	12,360	7,754	9,137	9,260	10,770	9,327	9,345
Com_530_pos	Methyl cinnamate	C10H10O2	162.19	9.88	163.07	Phenylpropanoids	87,946	34,886	37,639	262,000	266,000	287,000	53,740	57,150	34,820
Com_176_neg	Methyl indole-3-acetate	C11H11NO2	189.08	0.70	188.10	Amino Acid And Its Derivatives	116,800	54,770	71,520	98,750	102,700	95,440	102,800	90,700	119,400
Com_532_pos	Methyl linoleate	C19H33O2	294.47	15.28	295.26	Lipids	523,500	106,200	120,200	180,800	174,200	197,300	354,900	414,200	411,100
Com_419_pos	methylApigenin C-hexoside	C22H22O10	446.40	6.52	447.13	Flavonoids	39,133	9,243	2,575	49,456	52,237	47,273	11,410	26,420	24,800
Com_418_pos	methylApigenin C-pentoside	C21H20O9	416.38	6.27	417.12	Flavonoids	100,800	4,592	11,680	77,433	75,876	71,520	20,850	15,590	33,930
Com_44_neg	Methylenesuccinic acid	C5H6O4	130.10	0.62	131.00	Lipids	26,060	10,750	10,220	4,711	3,626	6,278	5,142	9,484	11,020
Com_46_neg	Methylmalonic Acid	C4H6O4	118.09	0.56	117.00	Organic Acid And Its Derivatives	410,100	68,120	37,860	403,400	547,700	362,600	98,920	77,370	48,670
Com_657_pos	methylQuercetin O-hexoside			6.08	479.00	Flavonoids	#########	#########	9,023,000	1,080,000	988,700	978,200	2,479,000	3,220,000	2,784,000
Com_1_neg	Mevalonic acid	C6H12O4	148.16	0.75	147.10	Organic Acid And Its Derivatives	436,400	86,490	131,800	635,600	645,900	667,500	317,300	246,500	268,400
Com_624_pos	Miltirone	C19H22O2	282.38	15.96	283.10	Others	8,722	11,050	11,290	6,234	9,674	15,150	21,950	11,410	19,720
Com_609_pos	Miyabenol C	C42H32O9	680.70	0.75	681.21	Phenols	25,750	4,619	8,514	19,060	9,876	12,920	9,197	18,370	10,990
Com_219_neg	Morin hydrate	C15H12O8	320.25	7.25	301.04	Flavonoids	292,700	208,500	132,900	31,890	27,490	24,870	34,620	38,420	40,000
Com_655_pos	Muscone	C16H30O	238.41	18.81	239.24	Others	476,600	437,500	453,500	393,100	534,100	419,200	529,800	479,100	418,300
Com_146_neg	Myricitrin	C21H20O12	464.38	6.86	463.09	Flavonoids	#########	#########	#########	7,908,000	7,647,000	6,916,000	#########	#########	#########
Com_145_neg	Myrtillin chloride	C21H21ClO12	500.84	9.08	481.10	Flavonoids	15,016	10,196	6,562	8,259	9,767	10,368	2,195	3,507	3,746
Com_480_pos	N- Feruloylcadaverine			14.94	279.00	Amino Acid And Its Derivatives	1,232,000	212,600	256,000	455,000	405,100	489,600	665,900	709,700	634,200
Com_428_pos	N-(2-Hydroxyethyl)iminodiacetic acid	C6H11NO5	177.16	0.81	178.07	Organic Acid And Its Derivatives	3,518,000	2,179,000	1,770,000	1,084,000	937,500	976,700	1,794,000	1,816,000	1,903,000
Com_240_neg	N-(3-Indolylacetyl)-L-Alanine	C13H14N2O3	246.26	5.95	267.07	Amino Acid And Its Derivatives	25,210	7,963	15,700	18,150	21,340	13,980	35,010	15,060	22,470
Com_641_pos	N-(9H-Purin-6-ylcarbamoyl)threonine	C10H12N6O4	280.24	17.02	281.10	Nucleotide And Its Derivates	13,570	27,920	44,640	13,520	14,290	12,140	11,960	10,070	8,504

Com_640_pos	N', N'', N'''-DiFeruloyl,Sinapoylspermidine			17.31	704.10	Phenols	5,590	6,366	8,804	4,142	6,891	4,171	6,687	4,062	7,740
Com_642_pos	N', N'', N'''-Di-p-hydroxybenxoyl, feruloylagmatine			17.30	548.10	Amino Acid And Its Derivatives	2,195	3,969	2,146	3,374	3,110	2,721	1,503	2,863	2,682
Com_643_pos	N', N'',N'''-Triferuloylagmatine			17.32	660.00	Amino Acid And Its Derivatives	5,907	6,957	4,439	4,647	5,151	5,089	4,560	4,048	5,592
Com_644_pos	N', N'',N'''-Tri-p-cinnamoylagmatine			4.45	522.00	Amino Acid And Its Derivatives	59,820	4,355	2,758	57,870	40,610	68,160	19,340	19,050	15,940
Com_341_pos	N', N''-Dicaffeoylspermidine			24.25	471.00	Amino Acid And Its Derivatives	11,400	9,248	10,190	14,710	10,120	9,974	9,939	13,180	12,690
Com_343_pos	N', N''-DiFeruloylagamtine-N-arabinoside			17.37	615.00	Phenols	39,670	42,960	39,290	35,330	33,680	34,570	49,880	45,650	32,710
Com_342_pos	N', N''-DiFeruloylspermidine	C27H35N3O6	497.58	15.75	498.26	Phenols	13,870	11,670	12,090	19,790	13,510	25,100	19,550	15,890	19,460
Com_610_pos	N', N''-DiSinapoylspermidine	C29H39N3O8	557.63	4.72	558.28	Phenols	7,490	6,306	14,647	62,450	70,880	50,570	18,180	21,370	19,620
Com_710_pos	N', N''-Feruloyl, Caffeoylspermidine	C26H33N3O6	483.56	0.65	485.00	Amino Acid And Its Derivatives	13,240	14,470	6,822	12,900	10,320	27,190	5,553	7,782	3,527
Com_102_neg	N-[(Tert-butoxy)carbonyl]-L-tryptophan	C16H20N2O4	304.34	10.27	303.13	Amino Acid And Its Derivatives	25,980	1,540	1,182	5,584	2,796	3,556	3,728	3,471	3,266
Com_369_pos	N1-Acetylspermine	C12H28N4O	244.38	1.13	245.20	Organic Acid And Its Derivatives	61,350	31,520	35,420	34,070	41,050	20,540	69,170	58,810	50,820
Com_623_pos	N2, N2-Dimethylguanosine	C12H17N5O5	311.29	1.59	312.13	Nucleotide And Its Derivates	2,318,000	1,236,000	1,128,000	550,000	476,600	419,700	542,800	536,100	580,000
Com_371_pos	N2-Methylguanosine	C11H15N5O5	297.27	1.07	298.20	Nucleotide And Its Derivates	180,200	124,000	124,700	41,710	41,900	38,840	50,990	76,030	28,130
Com_457_pos	N6,N6-dimethyladenine	C7H9N5	163.18	0.63	164.10	Nucleotide And Its Derivates	1,250,000	713,300	327,700	1,366,000	1,008,000	1,137,000	746,500	863,500	867,600
Com_370_pos	N6-Acetyl-L-Lysine	C8H16N2O3	188.22	0.76	189.12	Amino Acid And Its Derivatives	368,200	556,700	698,600	138,700	121,200	122,500	474,600	387,800	363,500
Com_686_pos	N6-Succinyl Adenosine	C14H17N5O8	383.31	1.27	384.12	Nucleotide And Its Derivates	3,399,000	2,193,000	769,000	2,452,000	2,094,000	2,044,000	2,297,000	2,540,000	2,557,000
Com_85_neg	N7-Methylguanosine	C11H17N5O5	298.28	3.90	296.10	Nucleotide And Its Derivates	378,600	254,700	325,000	82,470	86,880	84,230	122,000	135,700	131,200
Com_687_pos	N8-Acetylspermidine	C9H23Cl2N3O	260.20	0.65	188.18	Amino Acid And Its Derivatives	753,900	114,400	94,600	172,500	183,900	148,900	195,300	150,900	162,300
Com_101_neg	N-Acetylaspartate	C6H9NO5	175.14	0.59	174.04	Amino Acid And Its Derivatives	511,900	198,500	167,000	54,640	53,530	62,040	190,700	208,100	190,700
Com_100_neg	N-Acetylaspartylglutamic acid	C11H16N2O8	304.25	2.55	303.10	Organic Acid And Its Derivatives	1,550	463	1,063	8,723	7,773	7,406	4,170	4,266	3,598
Com_299_neg	N-Acetyl-D-Glucosamine	C8H15NO6	221.21	0.78	220.08	Carbohydrates	65,892	67,354	55,000	10,870	5,151	2,929	25,090	13,970	17,440
Com_689_pos	N-Acetyl-Dl-glutamic acid	C7H11NO5	189.17	0.61	190.07	Amino Acid And Its Derivatives	824,000	624,600	419,600	1,607,000	1,248,000	1,268,000	1,013,000	1,234,000	859,900

Com_688_pos	N-Acetyl-D-tryptophan	C13H14N2O3	246.26	5.53	247.11	Amino Acid And Its Derivatives	59,700	73,360	70,140	201,700	197,000	151,400	133,500	167,000	139,200
Com_52_neg	N-acetylglycine	C4H7NO3	117.10	0.70	116.00	Amino Acid And Its Derivatives	41,090	84,490	66,130	5,597	7,178	8,767	23,160	16,770	12,750
Com_685_pos	N-Acetyl-L-aspartic acid	C6H9NO5	175.14	5.81	176.07	Amino Acid And Its Derivatives	56,550	30,520	10,810	49,070	21,960	23,630	36,960	30,310	19,080
Com_185_neg	N-Acetyl-L-glutamic acid	C7H11NO5	189.17	0.67	188.10	Amino Acid And Its Derivatives	62,130	17,520	17,350	41,320	46,840	51,300	60,430	54,920	68,400
Com_690_pos	N-Acetyl-L-histidine	C8H11N3O3	197.19	0.74	198.09	Amino Acid And Its Derivatives	322,900	117,500	74,720	182,700	202,300	189,000	158,800	214,100	193,500
Com_691_pos	N-Acetyl-L-leucine	C8H15NO3	173.21	4.59	174.11	Amino Acid And Its Derivatives	98,720	20,570	36,596	21,490	41,250	48,070	60,630	55,640	51,050
Com_599_pos	N-Acetyl-L-Tyrosine	C11H13NO4	223.23	2.56	224.09	Amino Acid And Its Derivatives	35,210	6,183	9,315	10,920	11,930	13,560	20,520	18,290	21,550
Com_598_pos	N-Acetylneuraminic acid	C11H19NO9	309.27	19.24	310.10	Amino Acid And Its Derivatives	143,400	132,500	159,700	144,400	123,000	170,400	172,500	144,900	132,400
Com_597_pos	N-Acetylputrescine	C6H14N2O	130.19	0.74	131.00	Amino Acid And Its Derivatives	151,300	33,710	32,700	108,200	73,500	84,880	78,660	97,010	93,500
Com_603_pos	N-Acetylserotonin	C12H14N2O2	218.25	7.16	219.00	Amino Acid And Its Derivatives	54,006	12,460	21,246	72,260	49,250	53,950	33,720	30,940	19,790
Com_144_neg	N-Acetylthreonine	C6H11NO4	161.16	0.78	160.00	Amino Acid And Its Derivatives	160,000	99,110	233,100	149,900	138,800	142,700	129,000	108,700	132,900
Com_596_pos	N-Acetyltryptamine	C12H14N2O	202.25	0.83	203.10	Amino Acid And Its Derivatives	180,600	119,800	30,770	222,600	188,500	206,200	240,900	198,300	224,000
Com_380_pos	Nardosinone	C15H22O3	250.33	12.44	251.16	Terpenoids	#########	#########	#########	#########	#########	#########	#########	#########	#########
Com_19_neg	Naringenin	C15H12O5	272.25	8.24	271.00	Flavonoids	10,480	16,820	22,390	45,890	39,870	39,050	16,830	13,420	8,560
Com_594_pos	Naringerin	C15H12O5	272.25	7.80	273.08	Flavonoids	188,894	37,589	42,527	2,222,000	2,041,000	2,159,000	497,300	508,800	522,100
Com_20_neg	Naringin	C27H32O14	580.54	9.43	579.10	Flavonoids	36,290	108,100	23,611	13,660	11,150	9,655	19,530	23,230	20,620
Com_21_neg	Narirutin	C27H32O14	580.54	9.17	579.17	Flavonoids	43,580	111,500	12,940	11,640	12,740	13,040	20,990	15,870	19,150
Com_482_pos	N-Coumaroyltyramine	C17H17NO3	283.32	7.05	284.00	Amino Acid And Its Derivatives	2,246,000	9,739,000	400,200	#########	#########	#########	8,975,000	9,209,000	8,671,000
Com_444_pos	Neocryptotanshinone	C19H22O4	314.38	13.23	315.20	Others	282,300	106,900	41,820	26,160	38,390	40,490	52,120	43,570	39,070
Com_16_neg	Neoeriocitrin	C27H32O15	596.53	8.31	595.17	Flavonoids	37,550	19,820	16,250	56,030	49,440	51,140	17,940	20,270	13,870
Com_481_pos	Neotuberostemonine	C22H33NO4	375.50	12.19	376.25	Alkaloids	#########	#########	#########	#########	#########	#########	#########	#########	#########
Com_479_pos	N-Feruloylagmatine	C15H21N4O3	305.35	4.17	307.18	Phenols	11,350	13,180	6,921	10,590	11,730	9,428	22,330	11,490	7,912
Com_734_pos	N-Feruloylserotonin	C20H20N2O4	352.38	14.53	353.15	Phenols	21,050	22,740	7,697	47,790	44,210	49,400	38,650	36,500	42,230
Com_738_pos	N-Feruloylspermidine derivative			16.05	500.50	Phenols	7,982	7,959	2,127	5,355	6,420	8,015	34,800	32,660	37,640
Com_732_pos	N-Feruloyltyramine	C18H19NO4	313.35	7.37	314.14	Phenols	4,350,000	#########	1,175,000	#########	#########	#########	#########	#########	#########
Com_740_pos	NG,NG-Dimethyl-L-arginine	C8H20Cl2N4O2	202.25	0.80	203.00	Amino Acid And Its Derivatives	204,900	304,200	340,700	137,000	131,100	131,200	230,100	197,200	199,400
Com_729_pos	N-hexosyl-p-coumaroyl putrescine			7.75	397.10	Phenols	20,540	121,394	76,752	18,020	15,270	56,322	167,500	197,100	208,200

Com_741_pos	N-Hydroxy-L-tryptophan	C11H12N2O3	220.23	1.07	221.10	Amino Acid And Its Derivatives	99,600	228,200	134,300	101,600	92,610	79,920	87,410	95,880	84,070
Com_612_pos	Niacinamide	C6H6N2O	122.13	0.84	123.10	Terpenoids	3,459,000	1,490,000	1,354,000	1,986,000	1,812,000	1,809,000	1,195,000	1,231,000	1,168,000
Com_737_pos	Nicotianamine	C12H21N3O6	303.31	0.79	304.15	Others	1,940,000	7,432,000	4,457,000	5,690,000	4,875,000	5,056,000	2,946,000	3,225,000	3,234,000
Com_454_pos	Nicotinate ribonucleoside	C11H13NO6	255.22	0.76	256.20	Others	142,800	560,900	491,000	185,400	173,100	218,200	183,900	129,600	190,500
Com_445_pos	Nicotinic Acid	C6H5NO2	123.11	0.77	124.00	Terpenoids	1,892,000	1,727,000	1,537,000	1,038,000	881,400	892,500	1,198,000	1,215,000	1,079,000
Com_443_pos	Nicotinuric Acid	C8H8N2O3	180.16	9.67	181.10	Terpenoids	880,200	48,360	15,050	330,300	276,200	297,300	677,800	799,100	754,800
Com_440_pos	N-Lauryldiethanolamine	C16H35NO2	273.46	0.60	275.10	Others	49,660	32,790	27,350	94,050	67,490	59,300	61,280	52,730	77,200
Com_441_pos	Nobiletin	C21H22O8	402.40	10.91	403.10	Flavonoids	43,570	28,590	57,050	50,360	58,110	45,580	47,770	38,190	33,880
Com_648_pos	Nortanshinone	C17H12O4	280.27	20.37	303.00	Others	248,900	242,100	255,900	253,400	248,500	263,700	290,100	256,200	256,800
Com_552_pos	N-p-Cinnamoylputrescine	C13H18N2O	218.29	7.14	219.00	Amino Acid And Its Derivatives	52,680	70,350	9,853	489,000	412,900	451,700	192,300	199,100	193,200
Com_551_pos	N-p-Coumaroylagmatine	C14H20N4O2	276.33	15.30	277.17	Phenols	231,200	73,180	60,060	90,310	102,900	91,320	130,800	188,900	168,500
Com_550_pos	N-p-Coumaroylputrescine derivative			8.16	398.20	Phenols	102,300	4,545	8,316	15,390	8,392	21,920	20,340	34,320	29,150
Com_553_pos	N-Sinapoylagmatine	C16H24N4O4	336.39	18.28	337.19	Phenols	23,960	22,650	21,440	37,800	41,070	45,240	26,510	29,340	29,610
Com_377_pos	Nuciferin	C19H21NO2	295.38	10.78	296.17	Alkaloids	146,400	72,520	87,630	110,900	110,400	111,000	59,340	83,080	90,070
Com_434_pos	Nα-Acetyl-L-Arginine	C8H16N4O3	216.24	0.81	217.10	Amino Acid And Its Derivatives	302,000	87,070	58,840	329,100	380,000	340,000	1,531,000	1,247,000	1,359,000
Com_435_pos	N-Γ-Acetyl-N-2-Formyl-5-Methoxykynurenamine	C13H16N2O4	264.28	5.73	265.10	Organic Acid And Its Derivatives	103,100	21,040	31,908	110,400	113,500	85,210	58,660	58,660	36,760
Com_436_pos	O-Acetylserine	C5H9NO4	147.13	0.76	148.06	Amino Acid And Its Derivatives	9,026,000	#########	#########	5,400,000	5,195,000	4,757,000	#########	#########	#########
Com_432_pos	Oleic acid	C18H34O2	282.46	18.28	283.27	Lipids	24,480	12,200	26,030	45,140	61,230	50,670	18,790	20,920	18,300
Com_62_neg	Ononin	C22H22O9	430.13	0.73	429.13	Flavonoids	45,930	14,060	15,370	49,530	48,830	43,170	35,360	30,950	47,090
Com_319_neg	O-p-coumaroyl-O-salicyloyl quinic acid	C23H22O10	458.10	10.24	457.10	Organic Acid And Its Derivatives	175,300	71,990	24,300	2,185,000	1,998,000	2,039,000	951,700	1,028,000	985,600
Com_462_pos	O-Phosphocholine	C5H15ClNO4P	219.60	0.77	184.00	Others	653,700	3,599,000	5,694,000	6,606,000	5,620,000	5,799,000	1,794,000	1,802,000	1,792,000
Com_172_neg	O-Phosphorylethanolamine	C2H8NO4P	141.06	0.65	140.00	Others	46,130	128,400	199,100	32,510	60,380	63,840	37,430	36,490	39,360
Com_173_neg	Orcinol glucosid	C13H18O7	286.28	12.46	285.10	Phenols	114,100	130,400	166,800	105,900	120,300	109,000	166,200	142,800	121,200
Com_170_neg	Orotidine	C10H12N2O8	288.21	4.27	287.10	Nucleotide And Its Derivates	144,200	90,860	53,230	31,610	40,220	34,490	71,570	54,060	65,640
Com_463_pos	Oroxin B	C27H30O15	594.52	4.44	595.17	Flavonoids	781,600	95,480	43,230	419,000	372,800	338,700	638,800	688,900	743,900
Com_126_neg	Oxaloacetate	C4H4O5	132.07	0.63	131.00	Organic Acid And Its Derivatives	963,700	606,700	472,400	510,600	575,000	562,900	478,300	469,300	537,700
Com_692_pos	p-Aminobenzoate	C7H7NO2	137.14	5.47	137.30	Organic Acid And Its Derivatives	210,347	123,175	68,081	153,476	153,581	145,022	79,070	67,100	90,530
Com_346_pos	Panaxydol	C17H24O2	260.37	17.44	261.19	Lipids	74,760	35,790	23,480	125,100	151,200	139,100	47,190	60,570	56,850
Com_345_pos	p-Anisaldehyde	C8H8O2	136.15	6.16	137.06	Others	484,100	462,100	69,990	694,400	611,000	582,700	337,900	334,800	274,400
Com_668_pos	Pantothenate	C9H17NO5	219.24	1.96	220.12	Terpenoids	1,736,000	1,739,000	1,315,000	1,691,000	1,413,000	1,416,000	818,400	1,094,000	1,062,000
Com_60_neg	Pantothenol	C9H19NO4	205.13	0.90	204.13	Lipids	276,500	34,100	25,710	346,000	375,600	321,100	167,700	124,400	146,400
Com_344_pos	Parthenolide	C15H20O3	248.32	6.81	249.15	Others	1,370,000	317,900	175,100	350,500	283,100	296,500	520,500	538,800	507,400
Com_498_pos	p-Coumaraldehyde	C9H8O2	148.16	7.90	149.10	Phenylpropanoids	409,000	156,600	176,500	207,200	218,600	201,800	253,400	280,300	206,800

Com_694_pos	p-Coumaric acid	C9H8O3	164.16	3.64	165.20	Phenylpropanoids	########	########	########	106,700	103,800	124,500	5,068,000	5,598,000	5,273,000
Com_638_pos	p-Coumaroylagmatine			6.33	277.10	Phenols	26,330	8,591	2,796	8,014	9,930	5,053	12,770	28,930	17,360
Com_321_pos	Phellodendrine chloride	C20H24ClNO4	377.86	0.72	342.17	Alkaloids	46,650	29,950	37,730	24,700	32,540	28,220	19,760	40,240	23,600
Com_496_pos	Phellodenol H	C17H20O9	368.34	4.13	369.12	Phenols	46,790	31,410	124,404	1,295,000	1,211,000	1,240,000	200,400	254,900	261,500
Com_497_pos	Phellodenol H O-hexoside			4.91	531.50	Phenols	433,700	404,000	166,700	25,670	30,310	21,830	21,230	29,860	58,720
Com_382_pos	Phenethylamine	C8H11N	121.18	2.66	122.10	Amino Acid And Its Derivatives	604,100	256,100	171,700	705,400	579,900	596,200	404,900	446,100	417,800
Com_365_pos	Phenylacetylglutamine	C13H16N2O4	264.28	1.03	265.10	Amino Acid And Its Derivatives	1,170,000	2,555,000	1,912,000	70,990	79,430	97,950	478,500	457,900	521,300
Com_149_neg	Pheophorbide A	C35H36N4O5	592.68	18.58	591.26	Organic Acid And Its Derivatives	198,600	181,100	77,440	150,600	135,600	103,000	44,030	68,960	68,930
Com_582_pos	Phe-Phe	C18H20N2O3	312.36	4.92	313.15	Amino Acid And Its Derivatives	7,581	38,634	46,373	64,000	68,320	39,030	18,900	15,140	17,430
Com_615_pos	Phe-Pro	C14H18N2O3	262.30	4.48	263.14	Amino Acid And Its Derivatives	230,300	199,700	160,300	265,700	238,200	217,100	125,200	133,600	101,900
Com_614_pos	Phloretin	C15H14O5	274.27	7.75	275.09	Phenylpropanoids	68,896	32,799	31,890	639,100	556,100	534,700	76,730	138,900	127,600
Com_315_neg	Phloridzin	C21H28O12	472.44	5.94	437.14	Flavonoids	7,039	4,809	5,418	20,100	26,230	21,930	16,110	18,920	14,680
Com_431_pos	Phosphocreatine	C4H10N3O5P	211.11	1.08	212.10	Amino Acid And Its Derivatives	1,508,000	749,100	581,200	1,440,000	1,668,000	1,383,000	1,007,000	1,122,000	784,600
Com_160_neg	Phosphoenolpyruvic acid	C3H5O6P	168.04	0.61	167.10	Organic Acid And Its Derivatives	37,250	31,310	28,660	25,260	24,830	16,450	35,910	32,090	33,880
Com_123_neg	phosphoribosyl pyrophosphate	C5H13O14P3	196.25	0.62	189.00	Carbohydrates	56,760	38,970	58,320	19,050	32,700	26,030	16,440	14,160	12,780
Com_58_neg	Physcion	C16H12O5	284.26	11.97	283.06	Others	46,940	52,667	48,290	2,397	2,026	2,223	64,860	58,160	55,730
Com_59_neg	Physcion 8-O-Beta-D-Monoglucoside	C22H22O10	446.40	5.96	445.11	Others	95,355	77,209	63,510	11,960	16,360	15,050	34,930	37,220	41,500
Com_708_pos	Phytocassane E	C20H28O3	316.43	10.92	317.21	Terpenoids	14,020	53,614	51,728	61,750	56,960	51,800	57,440	30,750	28,200
Com_254_neg	Pinobanksin	C15H12O5	272.07	9.30	271.00	Flavonoids	32,270	20,670	9,797	28,800	34,260	25,120	17,170	14,700	14,010
Com_152_neg	Pinocembrin	C15H12O4	256.07	0.70	255.10	Flavonoids	41,730	22,850	18,180	41,040	46,820	40,190	22,240	31,170	27,900
Com_78_neg	PinoresinolDiglucoside	C32H42O16	682.68	7.91	679.06	Phenols	9,011	10,510	9,943	344,200	276,900	203,900	49,870	44,640	63,130
Com_23_neg	Pinoresinol-glucoside	C26H32O11	520.53	9.17	519.19	Phenylpropanoids	18,310	8,248	9,414	24,130	26,910	27,740	13,540	10,270	11,670
Com_706_pos	Pipecolic acid	C6H11NO2	129.16	0.79	130.09	Organic Acid And Its Derivatives	########	########	########	4,064,000	3,673,000	3,529,000	########	########	########
Com_54_neg	Plantagoside	C21H22O12	466.39	4.55	465.10	Flavonoids	253,700	173,100	162,400	9,769	5,558	11,430	31,200	26,820	28,190
Com_705_pos	p-Nitroaniline	C6H6N2O2	138.12	1.06	139.00	Others	93,870	162,400	98,700	314,300	282,700	298,900	216,600	197,000	206,800
Com_55_neg	Polydatin	C20H23O9	407.39	8.01	389.12	Phenylpropanoids	52,328	18,680	19,808	50,890	51,060	53,990	13,690	17,210	14,080
Com_385_pos	PolygalaxanthoneVI	C23H26O12	494.44	6.45	495.15	Others	92,540	45,810	77,860	23,340	24,060	27,510	16,280	20,740	18,170
Com_493_pos	PolygalaxanthoneVII	C27H32O16	612.54	5.26	613.18	Phenols	504,600	798,600	1,232,000	21,990	37,850	15,820	142,400	221,900	111,100
Com_386_pos	Polypodine B	C27H44O8	496.63	0.83	497.31	Terpenoids	343,700	243,500	389,100	337,600	308,900	326,500	151,800	206,500	209,800
Com_150_neg	Poncirin	C28H34O14	594.20	8.40	593.00	Flavonoids	572,000	1,890,000	849,100	2,584,000	2,223,000	2,342,000	660,800	683,300	746,200
Com_384_pos	Prim-O-glucosylcimifugin	C22H28O11	468.45	5.19	469.17	Flavonoids	427,400	47,080	76,380	679,100	656,500	536,900	939,500	1,200,000	1,046,000
Com_334_pos	Proline	C5H9NO2	115.13	0.75	133.15	Amino Acid And Its Derivatives	########	########	########	3,927,000	3,623,000	3,697,000	########	########	9,938,000
Com_294_neg	Protocatechuic Acid	C7H6O4	154.12	0.73	153.00	Phenols	########	2,193,000	2,410,000	1,663,000	1,856,000	1,964,000	2,697,000	2,734,000	2,555,000

Com_118_neg	Prunin	C21H22O10	434.39	9.40	433.12	Flavonoids	95,070	46,330	35,290	65,980	86,170	96,100	41,240	36,740	36,530
Com_122_neg	Prunindihydrochalcone	C21H24O10	436.41	7.38	435.13	Phenols	22,960	9,158	2,051	11,732	12,974	13,442	5,444	4,842	7,686
Com_611_pos	Przewaquinone A	C19H18O4	310.34	12.50	311.00	Others	237,700	257,500	221,400	246,600	242,900	230,400	317,500	190,600	234,900
Com_540_pos	Pseudoephedrine	C10H15NO	165.23	2.87	166.12	Alkaloids	1,361,000	218,200	249,100	36,290	25,190	32,720	138,500	149,400	147,200
Com_237_neg	Pseudouridine	C9H12N2O6	244.07	0.90	243.00	Nucleotide And Its Derivates	175,500	91,650	105,400	77,050	71,520	86,710	91,130	58,520	60,150
Com_549_pos	Psoralen	C11H6O3	186.16	8.12	187.04	Phenylpropanoids	33,530	19,540	32,022	44,310	51,690	39,080	33,950	34,570	44,370
Com_486_pos	Pteroside A	C21H30O8	410.46	4.53	411.20	Terpenoids	174,300	97,793	67,641	433,600	425,700	394,800	98,030	90,570	83,870
Com_409_pos	pterosin A	C15H20O3	248.32	8.97	249.15	Others	42,790	13,624	4,528	46,630	51,272	45,322	21,380	18,080	17,830
Com_339_pos	Pterostilbene	C16H16O3	256.30	0.61	257.12	Phenylpropanoids	94,470	49,160	33,550	176,900	263,800	151,400	113,100	155,200	165,700
Com_548_pos	Purine	C5H4N4	120.11	1.66	121.00	Nucleotide And Its Derivates	183,000	503,700	589,300	59,610	44,500	45,790	134,900	140,900	126,100
Com_305_neg	Pyridoxal 5'-Phosphate	C8H10NO6P	247.14	0.76	246.00	Terpenoids	31,790	19,650	9,600	31,910	29,330	18,370	26,010	24,970	15,270
Com_546_pos	Pyridoxine	C8H11NO3	169.18	0.85	170.10	Terpenoids	581,800	448,500	120,100	963,400	802,500	826,000	944,700	913,100	785,600
Com_682_pos	Pyrrole-2-Carboxylic Acid	C5H5NO2	111.10	0.73	112.10	Organic Acid And Its Derivatives	1,173,000	303,600	196,200	362,500	317,800	349,000	483,800	511,200	567,800
Com_53_neg	Pyrroloquinoline quinone	C14H6N2O8	330.01	23.50	329.00	Alkaloids	4,569,000	4,451,000	6,610,000	4,601,000	4,732,000	4,664,000	4,256,000	4,161,000	5,599,000
Com_257_neg	Pyruvic Acid	C3H4O3	88.06	0.59	87.01	Organic Acid And Its Derivatives	3,324,000	1,674,000	1,795,000	4,602,000	5,506,000	5,218,000	3,127,000	2,302,000	1,837,000
Com_681_pos	Quercetin	C15H10O7	302.24	6.78	303.05	Flavonoids	756,200	563,400	509,700	84,170	82,090	83,320	113,000	121,400	102,700
Com_679_pos	Quercetin 3-D-galactoside	C21H20O12	464.38	4.72	465.10	Flavonoids	#########	#########	#########	#########	9,865,000	#########	6,125,000	6,974,000	7,432,000
Com_207_neg	Quercetin 3-O-sophoroside	C27H30O1	626.52	5.95	625.14	Alkaloids	7,932,000	1,945,000	1,992,000	2,044,000	1,847,000	1,898,000	1,200,000	1,233,000	1,238,000
Com_205_neg	Quercetin 3-β-D-glucoside	C21H20O12	464.38	6.78	463.09	Carbohydrates	#########	#########	#########	#########	#########	#########	#########	#########	#########
Com_267_neg	Quercetin 4'-O-glucoside (Spiraeoside)	C21H20O12	464.10	7.40	463.00	Flavonoids	#########	#########	#########	3,600,000	3,298,000	3,365,000	5,671,000	6,232,000	5,216,000
Com_684_pos	Quercetin 5-O-hexoside			6.52	465.10	Flavonoids	1,201,000	4,650,000	2,556,000	386,300	375,600	349,200	374,600	589,300	558,400
Com_677_pos	Quercetin O-malonylhexoside			5.46	551.00	Flavonoids	644,200	864,400	920,500	119,200	56,890	86,090	127,600	185,300	209,600
Com_680_pos	Quercetin-3,4'-O-di-beta-glucopyranoside	C27H30O17	626.52	4.56	627.50	Flavonoids	6,701,000	1,895,000	2,042,000	908,400	957,800	1,139,000	617,100	799,900	804,200
Com_206_neg	Quercetin-3'-O-glucoside	C21H20O12	464.38	6.51	463.09	Flavonoids	#########	#########	#########	#########	9,795,000	#########	#########	#########	#########
Com_683_pos	Quercetin-O-glucoside	C21H20O12	464.38	6.52	465.10	Flavonoids	470,400	2,216,000	1,189,000	84,510	99,960	84,100	104,700	97,090	68,820
Com_581_pos	Quercitrin	C21H20O11	448.38	6.14	449.00	Flavonoids	118,500	470,500	178,300	16,780	18,110	10,230	61,840	60,550	40,760
Com_676_pos	Quinic acid	C7H12O6	192.17	0.81	193.10	Lipids	1,029,000	500,700	454,500	2,026,000	2,413,000	2,548,000	974,200	861,000	934,500
Com_249_neg	Quinolinic acid	C7H5NO4	167.12	0.67	166.02	Organic Acid And Its Derivatives	10,070	3,882	4,546	5,034	4,385	5,595	4,981	5,545	3,973
Com_9_neg	Raffinose	C18H32O16	504.44	0.90	503.44	Carbohydrates	3,213,000	2,476,000	3,740,000	2,133,000	2,180,000	2,381,000	2,076,000	2,288,000	2,219,000
Com_330_pos	Raspberry Ketone	C10H12O2	164.20	20.49	165.09	Phenols	13,364	8,249	9,209	14,331	15,429	15,315	5,591	5,106	8,034
Com_636_pos	Reduced glutathione	C10H17N3O6S	307.32	0.83	308.00	Amino Acid And Its Derivatives	27,190	35,110	28,920	26,310	34,930	26,210	36,830	40,180	27,720
Com_246_neg	Rehmannioside A	C21H32O15	524.47	8.56	523.17	Terpenoids	61,646	18,680	35,387	49,750	43,920	47,020	13,870	16,910	17,030
Com_22_neg	Rhamnetin	C16H12O7	316.26	1.60	315.00	Flavonoids	4,057,000	2,345,000	1,854,000	899,000	805,800	939,200	1,119,000	1,221,000	1,121,000
Com_739_pos	Rhodomyrtone	C26H34O6	442.54	13.03	460.40	Others	647,500	2,342,000	2,325,000	668,300	621,700	693,200	876,700	904,000	1,030,000
Com_251_neg	Rhoifolin	C27H30O14	578.52	7.78	577.16	Flavonoids	9,201	4,775	16,190	188,200	184,000	197,300	61,120	54,600	65,010

Com_120_neg	Ribitol	C5H12O5	152.07	0.70	151.00	Carbohydrates	218,300	56,520	17,290	25,970	29,730	34,460	575,000	425,500	361,000
Com_124_neg	Ribose 5-phosphate	C5H11O8P	230.11	0.62	229.00	Carbohydrates	31,410	51,870	40,490	16,670	28,160	11,760	30,540	31,120	23,270
Com_197_neg	Rutin	C27H30O16	610.52	6.66	609.10	Flavonoids	#########	#########	#########	3,254,000	3,127,000	3,071,000	#########	#########	#########
Com_433_pos	S-(5-Adenosy)-L-Homocysteine	C14H20N6O5S	384.41	0.82	385.00	Amino Acid And Its Derivatives	19,490	249,400	225,800	96,800	83,600	65,200	57,080	62,620	44,950
Com_275_neg	Salicylic acid O-glucoside	C13H16O8	300.10	2.66	299.10	Organic Acid And Its Derivatives	3,497,000	2,194,000	2,089,000	#########	#########	#########	3,324,000	3,345,000	3,550,000
Com_153_neg	Saponarin	C27H30O15	594.52	7.81	593.16	Others	609,700	54,970	656,400	288,500	287,800	267,000	568,500	592,900	568,000
Com_592_pos	Sattabacin	C13H18O2	206.28	4.81	207.20	Others	158,400	61,310	26,160	777,600	675,400	722,100	244,900	293,300	242,000
Com_591_pos	Schaftoside	C26H28O14	564.49	4.74	565.16	Flavonoids	509,700	178,600	39,590	386,600	382,500	323,700	312,000	361,200	375,700
Com_363_pos	Sclareol Glycol	C16H30O2	254.41	11.20	255.23	Others	26,500	20,230	27,680	23,800	18,630	23,160	22,660	16,510	27,100
Com_720_pos	Scopolamine	C17H21NO4	303.35	5.27	304.15	Alkaloids	1,903,000	3,580,000	2,174,000	208,800	225,700	197,800	575,100	665,000	629,400
Com_719_pos	Scopoletin	C10H8O4	192.17	4.48	193.10	Phenylpropanoids	69,500	27,135	36,223	115,600	122,500	94,680	34,870	26,630	25,100
Com_83_neg	Scutellarein	C15H10O6	286.24	14.67	285.04	Flavonoids	4,452	4,784	5,898	4,254	4,490	5,113	4,418	5,430	4,028
Com_84_neg	Sebacic acid	C10H18O4	202.25	0.63	201.10	Organic Acid And Its Derivatives	173,100	109,100	121,300	98,120	141,000	86,240	136,600	128,600	143,500
Com_398_pos	Secoisolariciresinol	C20H26O6	362.42	14.94	363.18	Phenols	6,294	5,790	8,441	1,676	2,108	1,456	1,856	5,752	6,115
Com_395_pos	Sedoheptulose Anhydride	C7H12O6	192.17	0.61	215.06	Carbohydrates	35,520	63,070	34,960	107,000	41,400	62,860	56,840	35,140	32,020
Com_517_pos	Serotonin	C10H12N2O	176.22	0.91	177.00	Amino Acid And Its Derivatives	61,950	77,070	37,130	71,870	107,500	86,350	119,300	53,080	79,010
Com_217_neg	Sibiricose A5	C22H30O14	518.47	5.90	517.16	Phenylpropanoids	9,273	20,200	12,830	11,220	9,203	8,619	6,315	6,838	8,968
Com_285_neg	Sibiricose A6	C23H32O15	548.49	6.29	547.17	Phenylpropanoids	38,200	69,230	67,370	13,550	13,960	12,360	12,930	16,660	15,450
Com_216_neg	Sibirioside A	C21H28O12	472.44	9.16	471.15	Others	168,100	571,300	589,500	13,540	17,870	15,220	122,200	106,200	112,300
Com_508_pos	Sibutramine	C17H26ClN	279.85	17.12	280.18	Others	21,470	65,870	94,320	19,240	21,860	18,310	20,430	24,050	24,320
Com_516_pos	Sildenafil	C22H30N6O4S	474.58	12.21	475.21	Others	36,620	70,160	61,950	57,380	44,070	36,250	43,600	37,470	42,950
Com_515_pos	Sinapoyl O-hexoside	C17H22O10	370.35	4.17	404.16	Phenols	27,140	16,080	15,005	11,260	8,995	8,536	10,710	7,381	5,809
Com_514_pos	Sinensetin	C20H20O7	372.37	11.68	373.10	Flavonoids	40,870	26,090	44,530	50,790	51,700	65,080	36,970	35,820	32,120
Com_270_neg	Sinigrin	C10H16KNO9S2	397.46	0.63	358.00	Others	222,900	133,300	139,600	553,000	558,700	488,700	285,900	248,200	286,500
Com_204_neg	Sinomenine	C19H23NO4	329.39	10.26	328.16	Alkaloids	5,867	4,337	7,430	5,862	4,808	5,410	8,868	10,710	14,260
Com_513_pos	S-Methyl-L-cysteine	C4H9NO2S	135.19	0.77	136.10	Amino Acid And Its Derivatives	2,059,000	755,700	535,800	1,008,000	982,200	983,000	858,400	893,400	803,800
Com_408_pos	Sodium Taurocholate	C26H44NNaO7S	537.69	24.63	538.28	Lipids	61,450	53,910	52,890	50,600	54,530	50,530	66,780	51,920	49,400
Com_512_pos	Sodium taurodeoxycholate	C26H44NNaO6S	521.69	10.98	522.29	Lipids	4,722	2,427	819	8,282	9,926	12,580	12,910	21,960	19,890
Com_410_pos	Sophoricoside	C21H20O10	432.38	6.15	433.11	Flavonoids	139,000	14,210	49,310	114,600	156,400	102,400	44,350	67,720	60,470
Com_400_pos	Spermidine	C7H19N3	145.25	0.50	146.20	Amino Acid And Its Derivatives	155,100	163,200	179,900	810,500	719,500	645,400	150,900	179,600	216,500
Com_411_pos	Spermidine derivative	C8H21N3O6	255.27	17.31	256.15	Phenols	4,141	9,405	12,750	3,524	2,287	4,001	4,972	5,070	3,705
Com_412_pos	Spermine	C10H26N4	202.34	0.56	203.20	Amino Acid And Its Derivatives	111,000	202,400	238,900	122,200	118,400	61,540	111,700	88,450	89,860
Com_402_pos	Stachydrine	C7H13NO3	159.18	0.78	144.10	Alkaloids	1,991,000	871,800	204,700	1,470,000	1,038,000	1,215,000	1,592,000	1,364,000	1,415,000
Com_193_neg	Stevioside	C38H60O18	804.87	10.29	803.37	Terpenoids	29,210	9,068	14,770	9,375	9,270	8,998	5,806	7,855	5,893
Com_381_pos	Stigmasterol	C29H48O	412.69	22.92	413.38	Lipids	3,963	1,453	2,878	3,372	6,403	4,797	8,121	3,773	3,022

Com_312_neg	Succinic acid	C4H6O4	118.09	0.89	117.02	Organic Acid And Its Derivatives	42,710	10,280	13,350	39,080	39,520	38,740	21,340	11,040	11,870
Com_288_neg	Succinic anhydride	C4H4O3	100.07	0.63	99.02	Organic Acid And Its Derivatives	212,900	71,050	74,870	273,600	442,100	388,500	190,700	147,300	127,900
Com_652_pos	Sugiol	C20H28O2	300.44	14.58	301.20	Terpenoids	5,596	22,016	22,837	201,700	175,800	195,300	26,930	39,080	40,050
Com_310_neg	Sulfosalicylic acid	C7H6O6S	218.18	0.66	216.98	Organic Acid And Its Derivatives	67,830	44,100	33,520	67,230	50,680	72,400	52,890	37,890	34,950
Com_500_pos	Swainsonine	C8H15NO3	173.21	0.71	174.11	Alkaloids	344,300	165,000	107,400	530,900	257,700	428,800	371,900	274,800	247,900
Com_478_pos	Syringetin 3-O-hexoside			3.30	509.10	Flavonoids	38,590	87,840	38,245	18,030	25,490	12,750	37,560	59,230	44,430
Com_272_neg	Syringic acid O-glucoside		360.10	1.10	359.10	Organic Acid And Its Derivatives	734,500	458,200	207,600	3,506,000	2,493,000	2,616,000	606,000	720,700	740,700
Com_578_pos	Syringin	C17H24O9	372.37	3.79	390.18	Phenols	28,114	14,318	17,935	53,060	61,990	49,040	5,549	10,410	8,074
Com_709_pos	Tanshinone II B	C19H18O4	310.35	11.16	311.00	Others	180,600	330,900	333,700	123,800	125,600	125,100	170,900	180,300	177,400
Com_187_neg	Taurine	C2H7NO3S	125.15	0.64	124.00	Amino Acid And Its Derivatives	194,000	56,200	23,770	49,500	59,720	51,900	53,990	39,280	30,950
Com_396_pos	Taurocholic acid sodium salt hydrate	C26H44NNaO7S	537.69	16.70	556.30	Lipids	765,300	694,200	736,100	779,200	718,600	702,900	926,800	803,900	696,600
Com_571_pos	Thiamin	C12H17N4OS	265.35	0.65	265.11	Terpenoids	897,400	981,300	673,600	319,900	350,200	341,700	264,900	327,400	320,300
Com_525_pos	Threonine	C4H9NO3	119.12	0.75	120.07	Amino Acid And Its Derivatives	294,100	444,500	740,700	90,690	85,750	106,700	241,200	234,800	222,400
Com_570_pos	Threonyl carbamoyl adenosine	C15H20N6O8	412.35	3.23	413.14	Nucleotide And Its Derivates	262,300	86,740	81,030	45,680	46,140	39,550	52,820	53,170	57,210
Com_569_pos	Thymidine	C10H14N2O5	242.23	1.06	243.10	Nucleotide And Its Derivates	173,200	81,300	67,990	111,304	107,789	110,298	105,800	30,220	56,420
Com_35_neg	Thymidine 5'-diphosphate			0.74	401.10	Nucleotide And Its Derivates	30,960	20,700	19,910	33,130	16,970	17,380	25,110	24,610	24,700
Com_573_pos	Thymine	C5H6N2O2	126.11	1.11	127.00	Nucleotide And Its Derivates	90,150	137,800	59,360	26,410	27,750	64,854	97,460	74,070	38,570
Com_579_pos	Tianshic acid	C18H37NO5	347.49	10.12	348.10	Lipids	648,200	91,240	86,940	52,490	58,700	54,240	196,400	205,500	199,600
Com_169_neg	Tiliroside	C30H26O13	594.52	7.69	593.13	Flavonoids	5,839,000	8,031,000	5,837,000	847,100	814,900	851,200	1,333,000	1,301,000	1,311,000
Com_583_pos	Timosaponin Al	C33H54O8	578.78	7.77	579.39	Others	193,400	18,010	60,910	91,360	101,400	111,200	99,160	89,240	106,900
Com_453_pos	Topotecan	C23H23N3O5	421.45	12.21	422.17	Alkaloids	2,443,000	4,691,000	3,828,000	3,764,000	3,104,000	2,863,000	2,844,000	3,130,000	3,033,000
Com_455_pos	trans-2-Hexenal	C6H10O	98.14	12.33	99.10	Others	#########	#########	#########	#########	#########	#########	#########	#########	#########
Com_335_pos	trans-3-Hydroxy-L-proline	C5H9NO3	131.13	0.81	132.06	Amino Acid And Its Derivatives	514,800	889,900	1,025,000	83,860	50,310	77,030	366,600	371,900	373,700
Com_336_pos	trans-3-Indoleacrylic acid	C11H9NO2	187.20	2.58	188.10	Organic Acid And Its Derivatives	4,833,000	#########	#########	5,233,000	4,441,000	4,702,000	4,618,000	5,047,000	4,959,000
Com_338_pos	trans-Zeatin riboside	C15H21N5O5	351.36	0.65	352.30	Nucleotide And Its Derivates	627,900	245,700	166,200	86,950	95,370	86,290	156,600	131,500	136,000
Com_337_pos	Tricetin O-hexoside			6.52	465.00	Flavonoids	1,106,000	4,079,000	2,050,000	349,800	249,200	318,400	464,500	493,000	535,300
Com_318_neg	Tricin 5-O-rutinoside		638.10	7.91	637.10	Flavonoids	18,340	9,635	13,900	9,022	10,500	8,041	8,272	2,807	5,550
Com_499_pos	Tricin di-O-hexoside			5.30	655.20	Flavonoids	9,490	2,894	14,230	8,460	7,135	2,636	5,781	5,515	3,121
Com_317_neg	Tricin O-saccharic acid		522.10	8.77	521.10	Flavonoids	536,700	175,400	35,890	1,260,000	1,268,000	1,285,000	409,800	344,100	402,800

Com_255_neg	Trifolin	C21H20O11	448.10	8.32	447.00	Flavonoids	4,246,000	2,627,000	1,214,000	183,000	177,900	175,600	670,800	723,000	747,600
Com_261_neg	Trifolirhizin +HCOOH	C22H22O10	446.40	18.58	491.12	Flavonoids	103,800	27,380	10,520	55,460	49,200	34,720	44,050	51,720	48,870
Com_452_pos	Trimethylamine N-Oxide	C3H9NO	75.11	0.85	76.00	Amino Acid And Its Derivatives	6,382	5,946	2,003	13,767	15,436	13,490	6,427	5,398	6,910
Com_505_pos	Tryptamine	C10H12N2	160.22	0.79	161.10	Alkaloids	138,800	211,200	299,300	141,000	150,400	128,600	155,900	157,500	144,300
Com_523_pos	Tyramine	C8H11NO	137.18	0.98	138.10	Others	44,880	410,400	157,300	391,300	339,700	371,700	141,700	101,100	116,800
Com_724_pos	Uracil	C4H4N2O2	112.09	0.72	113.00	Nucleotide And Its Derivates	792,100	425,900	263,800	761,700	627,800	660,600	690,700	691,400	600,700
Com_459_pos	Urea	CH4N2O	60.06	0.63	61.00	Others	20,050	50,810	50,790	46,100	53,730	43,300	71,860	73,920	70,650
Com_725_pos	Uridine	C9H12N2O6	244.20	0.91	245.10	Nucleotide And Its Derivates	939,900	397,100	270,000	646,100	676,200	573,100	600,100	462,500	546,600
Com_225_neg	Uridine 5'-diphosphate	C15H24N2Na2O18P2	466.14	0.57	463.00	Nucleotide And Its Derivates	59,870	61,290	71,330	35,630	30,590	33,050	42,220	36,700	38,560
Com_77_neg	Uridine 5'-diphospho-D-glucose	C15H24N2O17P2	566.10	0.68	565.10	Nucleotide And Its Derivates	145,200	227,700	330,800	46,280	65,760	86,610	60,830	34,520	44,870
Com_50_neg	Uridine 5'-monophosphate	C9H13N2O9P	324.04	0.80	323.00	Nucleotide And Its Derivates	511,000	177,900	165,000	217,600	238,700	186,600	157,500	117,500	147,100
Com_717_pos	Val-Ser	C8H16N2O4	204.22	0.86	205.12	Amino Acid And Its Derivatives	168,100	120,700	96,700	216,600	165,600	216,200	120,900	171,500	118,200
Com_17_neg	Vanillic acid	C8H8O4	168.15	4.18	167.04	Organic Acid And Its Derivatives	657,900	104,800	167,600	21,554	20,820	19,970	59,330	53,880	47,990
Com_659_pos	vasicine	C11H12N2O	188.23	2.45	189.10	Alkaloids	777,900	3,897,000	3,128,000	827,000	736,200	752,000	792,500	871,800	819,500
Com_521_pos	Viscumamide	C30H55N5O5	565.79	24.15	566.20	Amino Acid And Its Derivatives	76,660	84,430	75,440	78,580	66,130	72,180	117,400	86,110	77,660
Com_663_pos	Vitamin B2	C17H20N4O6	376.36	4.24	377.00	Terpenoids	749,100	188,800	186,000	141,100	151,600	154,100	206,700	222,100	201,000
Com_645_pos	Vitexin	C21H20O10	432.38	4.80	433.11	Flavonoids	218,500	31,040	323,300	116,900	89,790	102,700	163,200	183,500	199,900
Com_99_neg	Vitexin-2-O-rhamnoside	C27H30O14	578.52	7.79	577.16	Flavonoids	7,038,000	780,900	645,300	7,923,000	7,532,000	7,762,000	#########	#########	#########
Com_95_neg	Wogonin	C16H12O5	284.26	9.89	283.06	Flavonoids	7,637	30,930	11,505	180,600	154,500	168,200	52,120	44,600	37,820
Com_328_pos	Xanthine	C5H4N4O2	152.11	0.79	153.04	Nucleotide And Its Derivates	378,100	415,600	574,900	157,000	142,500	140,300	350,200	316,000	312,000
Com_96_neg	Xanthosine	C10H12N4O6	284.23	1.16	283.07	Nucleotide And Its Derivates	271,700	178,800	136,700	29,980	30,160	60,620	95,470	68,040	52,080
Com_56_neg	Xanthurenic acid	C10H7NO4	205.17	2.36	204.03	Organic Acid And Its Derivatives	61,250	25,910	16,080	14,790	10,670	16,430	685,500	612,800	380,100
Com_545_pos	Xanthurenic acid O-hexoside	C16H17NO9	367.31	1.59	368.10	Amino Acid And Its Derivatives	1,059,000	719,800	641,500	687,600	609,500	558,000	1,103,000	1,143,000	1,223,000
Com_227_neg	Ziyuglycoside II	C35H56O8	604.81	14.57	603.39	Others	10,690	7,982	7,086	8,794	7,846	8,419	2,417	2,508	2,773
Com_226_neg	α-Benzylsuccinic acid	C11H12O4	208.21	0.93	189.06	Organic Acid And Its Derivatives	77,870	46,460	46,390	43,260	68,830	44,600	75,810	67,670	77,170
Com_509_pos	α-Nicotinamide adenine dinucleotide	C21H27N7O14P2	663.43	0.72	664.12	Nucleotide And Its Derivates	8,371	174,900	172,700	74,420	59,500	40,230	36,360	38,550	29,480

Com_595_pos	β-Alanine	C3H7NO2	89.09	0.66	90.00	Amino Acid And Its Derivatives	515,300	152,000	206,800	385,500	546,400	571,100	812,600	368,500	546,700
Com_220_neg	β-D-glucopyranosyl-caffeic acid			0.63	341.10	Phenols	922,700	413,900	329,500	114,600	130,400	95,710	247,100	238,700	147,200
Com_106_neg	γ,γ-Dimethylallyl pyrophosphate	C5H12O7P2	246.09	0.71	245.00	Others	140,600	81,600	131,300	49,300	45,620	67,690	135,300	117,800	161,700
Com_466_pos	γ-Glu-Leu	C11H20N2O5	260.29	2.91	261.15	Amino Acid And Its Derivatives	141,200	375,600	198,700	26,560	21,790	24,060	267,800	297,600	284,400
Com_467_pos	Γ-Glutamate-Cysteine	C8H14N2O5S	250.27	0.77	251.07	Amino Acid And Its Derivatives	457,800	65,390	38,260	197,600	169,300	183,300	116,200	123,100	143,800
Com_468_pos	δ-Hexanolactone	C6H10O2	114.14	0.71	115.09	Others	438,200	277,100	231,700	700,400	627,200	595,800	412,300	473,400	437,400

[1] MW molecular weight. RT retention time. Q1 parent ion. QC quality control.
