# Supplementary figures and images for: Revealing of Intracellular Antioxidants in Dendrobium nobile by High Performance Liquid Chromatography-Tandem Mass Spectrometry
Source: Metabolites. 2023 May 28;13(6):702. doi: 10.3390/metabo13060702 (PMC10303210; doi:10.3390/metabo13060702)

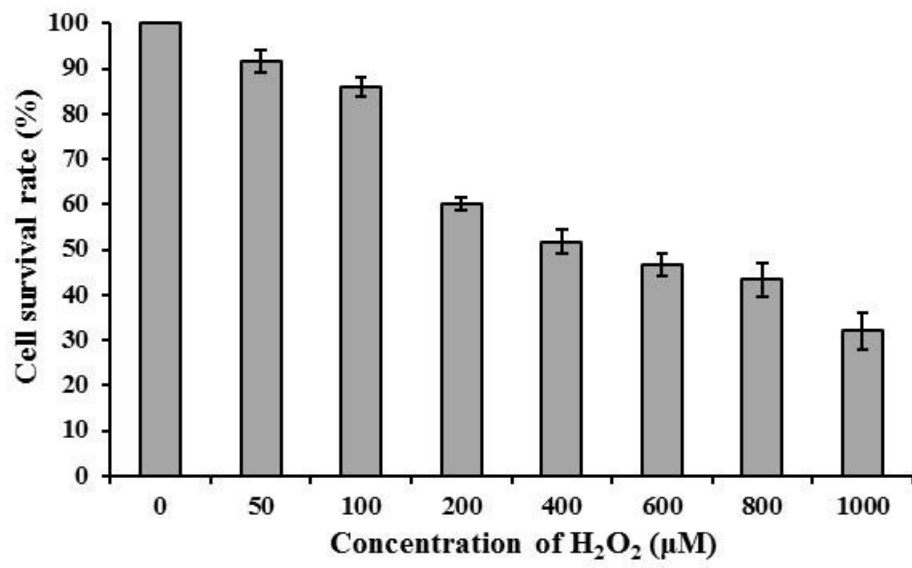

**Fig S1** The survival rates of H293T cells under different concentrations of H<sub>2</sub>O<sub>2</sub>.

Supplement: Supplementary file 1 [file metabolites-13-00702-s001.zip › Figure S1.pdf]
